# Structural descriptor for enhanced spin-splitting in 2D hybrid perovskites

**DOI:** 10.1038/s41467-021-25149-7

**Published:** 2021-08-17

**Authors:** Manoj K. Jana, Ruyi Song, Yi Xie, Rundong Zhao, Peter C. Sercel, Volker Blum, David B. Mitzi

**Affiliations:** 1 Thomas Lord Department of Mechanical Engineering and Materials Science, Duke University, Durham, NC USA; 2 Department of Chemistry, Duke University, Durham, NC USA; 3 University Program in Materials Science and Engineering, Duke University, Durham, NC USA; 4 Center for Hybrid Organic Inorganic Semiconductors for Energy, Golden, CO USA; 5 Department of Applied Physics and Materials Science, California Institute of Technology, Pasadena, CA USA; 6 Present Address: School of Physics, Beihang University, Beijing, China

**Keywords:** Two-dimensional materials, Spintronics, Electronic devices, Electronic structure

## Abstract

Two-dimensional (2D) hybrid metal halide perovskites have emerged as outstanding optoelectronic materials and are potential hosts of Rashba/Dresselhaus spin-splitting for spin-selective transport and spin-orbitronics. However, a quantitative microscopic understanding of what controls the spin-splitting magnitude is generally lacking. Through crystallographic and first-principles studies on a broad array of chiral and achiral 2D perovskites, we demonstrate that a specific bond angle disparity connected with asymmetric tilting distortions of the metal halide octahedra breaks local inversion symmetry and strongly correlates with computed spin-splitting. This distortion metric can serve as a crystallographic descriptor for rapid discovery of potential candidate materials with strong spin-splitting. Our work establishes that, rather than the global space group, local inorganic layer distortions induced via appropriate organic cations provide a key design objective to achieve strong spin-splitting in perovskites. New chiral perovskites reported here couple a sizeable spin-splitting with chiral degrees of freedom and offer a unique paradigm of potential interest for spintronics.

## Abstract (web summary)

Two-dimensional hybrid perovskites exhibiting Rashba/Dresselhaus spin-splitting can be potentially used for spin-selective transport and spin-orbitronics, yet the structural determinants of spin-splitting are not well-understood. Here, the authors reveal a specific inorganic layer distortion that correlates with bulk spin-splitting in these materials.

## Introduction

Two-dimensional (2D) metal halide perovskites (MHPs) are natural quantum well (QW) heterostructures of organic and inorganic sublattices with exceptional optoelectronic and photophysical properties^1–3^. They feature rich structural and compositional versatility owing to numerous possible templating organic cations^4,5^ that play a direct role in exciton dielectric confinement^6^, QW tuning^7–9^, singlet-to-triplet conversion^10^, high-temperature ferroelectricity^11^, nonlinear optical properties^12^, and chiral-optoelectronic functionality^13–16^. Additionally, a complex interplay of electrostatic requirements, steric effects, intermolecular interactions, and hydrogen-bonding associated with the organic cations engenders intricate distortions within the inorganic layers that have been previously correlated with exciton energy shifts^17,18^ and exciton self-trapping^19^. When such distortions break the inversion symmetry of the inorganic layer, Rashba/Dresselhaus spin-splitting can emerge due to spin–orbit coupling (SOC)^20–24^. Rashba/Dresselhaus spin-splitting is a relativistic quantum phenomenon in condensed matter that leads to important physical manifestations for emerging spin-orbitronic devices, including intrinsic spin-Hall effect^25^, gate-controlled spin precession^26^, (inverse) spin galvanic effects, photogalvanic effects^27^, and chiroptic effects^28^, that rely on SOC-mediated manipulation of the spin degrees of freedom by electrical, optical, or magnetic means^22,25^.

In 2D MHPs, specific conformational and packing characteristics of the organic cation may cause a noncentrosymmetric global space group, while a chiral cation always necessitates a chiral (Sohncke) global space group (Supplementary Fig. 1). Since strong SOC effects mainly arise in the inorganic component via heavy elements, merely relying on the global space groups without examining the inorganic layer distortions and the resulting local symmetry is, therefore, insufficient to provide a microscopic mechanism of spin-splitting in MHPs. A large Rashba/Dresselhaus spin-splitting ($\gg\!{kT}$) suppresses spin-flipping and is a critical requisite for room-temperature spin-based applications. There have been a few reports with computed spin-splitting values ranging from <<10 meV in some 2D lead-iodide-based MHPs^29,30^ to >200 meV in the chiral [S/R-1-1-NEA]_2_PbBr_4_^31^. Pb being the same heavy-metal constituent in these MHPs, SOC alone cannot justify the wide disparity in the predicted spin-splitting values. Despite a growing interest in spin-related properties of MHPs, a fundamental understanding of the symmetry breaking and the principles determining the spin-splitting magnitude are generally lacking, posing a major bottleneck in rational discovery of potential MHPs with sizable spin-splitting for prospective spin-based applications.

**Fig. 1:**
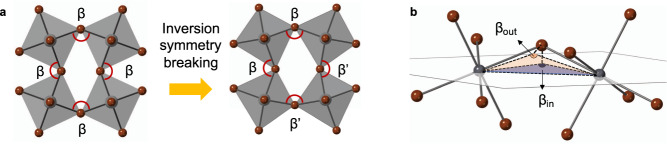
Symmetry breaking in 2D MHPs. **a** Schematic showing symmetric (left) and asymmetric (right) tilting of MX_6_ octahedra. Symmetric titling leads to a single M–X-M bond angle ($\beta$) and maintains inversion symmetry. Asymmetric tilting breaks the inversion symmetry by creating two M–X-M bond angles ($\beta$, $\beta {^\prime}$) with a bond angle disparity, $\Delta \beta \,=\,\beta$ − $\beta {^\prime}$. **b** Projections of in-plane (${\beta }_{{in}}$) and out-of-plane (${\beta }_{{out}}$) components of $\beta$.

From a materials discovery perspective, a key question is if there exists a simple structural parameter that controls the spin-splitting in MHPs and, consequently, their spin-related properties in applications. Remarkably, we here identify such a parameter from several possible modes of lattice distortions, providing a quantitative connection between the crystallographic structure of a 2D MHP and the spin-splitting exhibited in its conduction bands (CBs). Through crystallographic studies combined with density-functional theory (DFT), we establish that the computed spin-splitting mainly originates from and scales with in-plane asymmetric tilting of adjacent metal halide octahedra, represented by a specific projected bond angle difference $\Delta {\beta }_{{in}}$ (Fig. 1 and Supplementary Notes 1 and 2), as demonstrated for a broad array of MHPs, including five newly synthesized chiral MHPs. A large $\Delta {\beta }_{{in}}$ signifies inversion asymmetry within inorganic layers, and in turn, serves as a key local descriptor of spin-splitting, beyond the quantitatively incomplete condition based on noncentrosymmetric global space groups. Moreover, some of the chiral MHPs reported here present a unique combination of chiral degrees of freedom (e.g., enabling chiral induced spin selectivity, CISS^32,33^) and sizeable distortion-induced spin-splitting, which is of potential interest for spintronic devices.

## Results

### Inorganic layer distortions and asymmetry in 2D MHPs

**Fig. 2:**
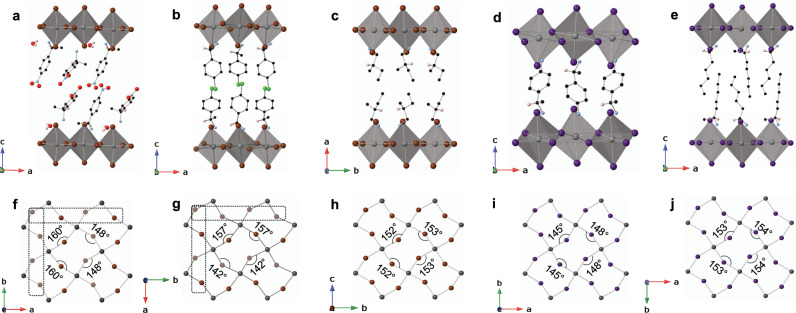
Structural characteristics of chiral MHPs. **a**–**e** Schematic single-crystal X-ray structures of [S-4-NO_2_-MBA]_2_PbBr_4_.H_2_O (**a**), [R-4-Cl-MBA]_2_PbBr_4_ (**b**), [S-2-Me-BuA]_2_PbBr_4_ (**c**), [S-4-NH_3_-MBA]PbI_4_ (**d**), and [S-MHA]_2_PbI_4_ (**e**). In each case, all H atoms except the one on the chiral carbon stereocenter are omitted for clarity. **f**–**j** In-plane views of perovskite layers corresponding to (**a**–**e**) showing the equatorial Pb–X–Pb bond angles ($\beta$). Axial X atoms are omitted for clarity. The two distinct equatorial Br atoms associated with widely disparate $\beta$ angles in (**f**, **g**) are distinguished by shaded and solid spheres. The dashed rectangles differentiate the individual rows comprised of the same type of equatorial Br atoms from the rows comprising alternating types of equatorial Br atoms, highlighting the local geometry fluctuation along one of the in-plane directions. In (**h**–**j**), the two unique equatorial X atoms are geometrically nominally equivalent with similar $\beta$ angles. Gray, brown, purple, red, green, blue, black, and pink spheres denote Pb, Br, I, O, Cl, N, C, and H atoms, respectively.

A core objective of this paper is to correlate specific structural distortions in 2D MHPs with sizeable Rashba/Dresselhaus spin-splitting. Importantly, it is not sufficient to merely include chiral spacer cations for the inorganic layers to become chiral (i.e., devoid of inversion and mirror symmetries). A detectable inversion asymmetry depends on the degree and nature of cation-induced distortions within the inorganic layers. To understand how a substantial inversion asymmetry manifests itself within the inorganic layers and how it is related to the use of a chiral spacer cation, we examine the experimentally determined structural properties of nine MHPs with noncentrosymmetric space groups, as well as of eight MHPs with chiral space groups, of which five are specifically synthesized and characterized for this work: [S-4-NO_2_-MBA]_2_PbBr_4_∙H_2_O, [R-4-Cl-MBA]_2_PbBr_4_, [S-2-Me-BuA]_2_PbBr_4_, [S-4-NH_3_-MBA]PbI_4_, and [S-MHA]_2_PbI_4_ (Fig. 2) (see “Methods” for details). Table 1 summarizes for all the above MHPs the metrics of interoctahedral tilting distortion leading to equatorial Pb–X–Pb bond angle $\beta$ < 180°, the disparity in adjacent $\beta$ angles ($\Delta \beta$) (see Fig. 1), as well as the intraoctahedron distortions: $\Delta d$ and ${\sigma }^{2}$. Here, $\Delta d$ is the bond length distortion defined as $\Delta d\,=\,\left(\frac{1}{6}\right)\Sigma {({d}_{i}\,-\,{d}_{0})}^{2}/{{d}_{0}}^{2}$ (${d}_{i}$ denotes the six Pb–X bond lengths and ${d}_{0}$ is the mean M–X bond length), and ${\sigma }^{2}$ is the bond angle variance defined as ${{{\sigma }}}^{2}\,=\,{\Sigma }_{i\,=\,1}^{12}{\left({\theta }_{i}\,-\,90\right)}^{2}/11$ (${\theta }_{i}$ denotes the individual *cis* X–Pb–X bond angles)^18^.

**Table 1:** Summary of global vs. local symmetries and various inorganic layer distortion metrics in 2D chiral MHPs, and noncentrosymmetric achiral MHPs.

2D HOIP	Global		Inorganic framework		Isolated inorganic layer		${{{{{\boldsymbol{\Delta }}}}}}{{{{{\boldsymbol{d}}}}}}$ (10^−5^)	${{{{{{\boldsymbol{\sigma }}}}}}}^{{{{{{\boldsymbol{2}}}}}}}$ (°^2^)	*β*, *β′* (°)	*∆β* (°)	PBE band structure
[C_9_DA]PbI_4_	Cc	C_s_	C2/c	C_2h_	P2_1_/c	C_2h_	3.45	4.33	153, 154	1	–
[4AMP]PbI_4_	Pc	C_s_	Pbam	D_2h_	Pbam	D_2h_	45.7	9.46	154, 155	1	Ref. ^39^
[S-MHA]_2_PbI_4_^a^	P2_1_2_1_2_1_	D_2_	P2_1_2_1_2_1_	D_2_	P4/mbm	D_4h_	10.5	10.78	153, 154	1	This work
[S-2-Me-BuA]_2_PbBr_4_^a^	P2_1_	C_2_	P2_1_/c	C_2h_	P2_1_/c	C_2h_	0.19	7.78	152, 153	1	This work
[4-Cl-BzA]_2_PbI_4_	P2_1_	C_2_	P2_1_/c	C_2h_	P2_1_/c	C_2h_	10	6.48	154, 155	1	Ref. ^30^
[4-Br-BzA]_2_PbI_4_	P2_1_	C_2_	P2_1_/c	C_2h_	P2_1_/c	C_2h_	8.64	8.39	153, 156	3	Ref. ^30^
[4AMPY]PbI_4_	Pn	C_s_	Pmmn	D_2h_	Pmmn	D_2h_	4.90	0.69	149, 152	3	–
[R-4-Cl-MBA]_2_PbI_4_	P1	C_1_	P-1	C_i_	P-1	C_i_	34.74	12.74	152, 155	3	This work
[S-4-NH_3_-MBA]PbI_4_^a^	P2_1_2_1_2_1_	D_2_	P2_1_2_1_2_1_	D_2_	P2_1_/c	C_2h_	38	14.13	145, 148	3	This work
[3AMPY]PbI_4_	Pn	C_s_	Pna2_1_	C_2v_	P2_1_/c	C_2h_	5.45	7.75	163, 168	5	–
[S-MBA]_2_PbI_4_	P2_1_2_1_2_1_	D_2_	Pnma	D_2h_	Pmc2_1_	C_2v_	56.44	20.11	151, 157	6	This work
[S-4-NO_2_-MBA]_2_PbBr_4_⋅H_2_O^a^	P2_1_	C_2_	P2_1_	C_2_	P2_1_	C_2_	129.4	34.04	148, 160	12	This work
[NMA]_2_PbBr_4_	Cmc2_1_	C_2v_	Cmc2_1_	C_2v_	Pmc2_1_	C_2v_	15.43	13.57	142, 154	12	This work
[PMA]_2_PbCl_4_	Cmc2_1_	C_2v_	Cmc2_1_	C_2v_	Pmc2_1_	C_2v_	4.22	21.42	142, 154	12	This work
[4AMP]PbBr_4_	Pca2_1_	C_2v_	Pca2_1_	C_2v_	Pca2_1_	C_2v_	105	40.83	146, 160	14	This work
[S-1-1-NEA]_2_PbBr_4_	P2_1_	C_2_	P2_1_	C_2_	P2_1_	C_2_	62	40.17	144, 158	14	This work
[R-4-Cl-MBA]_2_PbBr_4_^a^	P2_1_2_1_2_1_	D_2_	P2_1_2_1_2_1_	D_2_	P2_1_	C_2_	135.3	49.65	142, 157	15	This work

MHPs are sorted based on $\Delta \beta$ values along one of the in-plane directions (see Fig. 2f, g), which correlate strongly with the spin-splitting magnitude as shown in Fig. 5.

^a^New chiral MHPs reported in this study.

In all MHPs listed in Table 1, regardless of whether the spacer cation is chiral or achiral, there are two inequivalent $\beta$ angles in each MHP, leading to $\Delta \beta$ ≠ 0 (Figs. 1,  2f–j). We can group the different MHPs in Table 1 based on $\Delta \beta$ values. *High* $\varDelta \beta$*:* In [S-4-NO_2_-MBA]_2_PbBr_4_∙H_2_O and [R-4-Cl-MBA]_2_PbBr_4_, the same type of equatorial Br atom comprises the individual rows along one in-plane direction due to 2_1_ screw symmetry. However, two very different equatorial Br atoms alternate along the other in-plane direction, leading to $\Delta \beta$ as large as 12° and 15° in [S-4-NO_2_-MBA]_2_PbBr_4_∙H_2_O and [R-4-Cl-MBA]_2_PbBr_4_, respectively (Fig. 2f, g). A similar scenario with a large $\Delta \beta$ along one of the in-plane directions occurs in chiral [S/R-1-1-NEA]_2_PbBr_4_, as well as noncentrosymmetric achiral [NMA]_2_PbBr_4_, [PMA]_2_PbCl_4_, and [4AMP]PbBr_4_ (Table 1 and Supplementary Fig. 2). In all these MHPs, the fluctuating local geometry with $\Delta \beta$ as large as ~12°–15° along either in-plane direction breaks the inversion symmetry within the isolated inorganic layer (Table 1) as suggested by PLATON^34^ symmetry analysis (see Methods for details). *Low* $\varDelta \beta$*:* The rest of the chiral and achiral MHPs in Table 1 exhibit a typical situation encountered in known centrosymmetric MHPs with zero or very small $\Delta \beta$ (see Fig. 2h–j). Despite the global chiral space groups, the constituent isolated inorganic layers in these MHPs are nearly centrosymmetric from PLATON analysis due to symmetric disposition of $\beta$ angles (Table 1). The global space group alone is, therefore, insufficient as a criterion to predict inversion-asymmetry-induced Rashba/Dresselhaus SOC effects that primarily originate from the inorganic layers. The actual distortions and resulting local symmetry within the inorganic layer are crucial aspects. Specifically, a large $\Delta \beta$ points to a substantial inversion asymmetry in the inorganic layers as realized in specific chiral and noncentrosymmetric achiral MHPs, especially those comprising lead bromide or lead chloride perovskite layers (Table 1). Note that there is no clear correlation between $\Delta \beta$ and $\Delta d$ or ${{{\sigma }}}^{2}$ for the MHPs in Table 1 (Supplementary Fig. 5). More importantly, the values of $\Delta \beta$, $\Delta d$, and ${\sigma }^{2}$ found in lead-iodide MHPs are at least 2.5 times smaller than those found in most lead bromide, and lead chloride MHPs in Table 1 (Supplementary Figs. 5, 6), consistent with the fact that the inorganic layers in lead-iodide MHPs are typically nominally centrosymmetric (Table 1).

In contrast to Table 1, a survey of 56 2D MHPs with centrosymmetric global space groups reveals that they seldomly exhibit a substantial $\Delta \beta$ (Supplementary Table 1). In a few centrosymmetric cases, a relatively large $\Delta \beta$ does occur. However, in two of these cases (Supplementary Fig. 7b, c), equal $\beta$ angles are found on opposite sides of squares formed by Pb atoms in the structure so that an inversion center is retained. In contrast, the above chiral and noncentrosymmetric MHPs have unequal $\beta$ angles on opposite sides of squares defined by Pb. In the case of 2-fluoroethylammonium lead chloride ((FC_2_H_4_NH_3_)_2_PbCl_4_), $\Delta \beta$ = 11.7° also occurs across opposite sides of the Pb-defined squares (Supplementary Fig. 7a), leading to inversion asymmetric inorganic layers; in this case, an inversion center is found between the inorganic layers.

### Spin-splitting in 2D MHPs

**Fig. 3:**
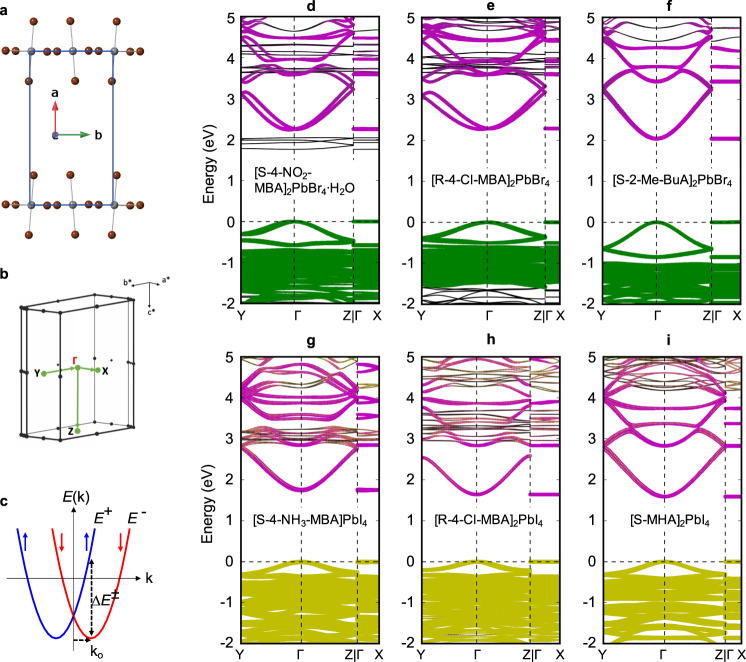
Spin-splitting in chiral MHPs. **a** A representative unit cell of a relaxed MHP (organic cations are omitted) and (**b**) the corresponding Brillouin zone in the reciprocal space used for band structure calculations. **c** Schematic representation of spin-polarized subbands separated in k-space along one dimension of a Rashba or Dresselhaus semiconductor. The ↑ and ↓ arrows denote, respectively, +1/2 and −1/2 like spin character. ${E}^{-}$ and ${E}^{+}$ denote the two spin subbands, and $\Delta {E}^{\pm }$ denotes the energy difference between them at a characteristic momentum offset, ${{{{{{\bf{k}}}}}}}_{0}$. **d**–**i** DFT-PBE+SOC band structures of chiral [S-4-NO_2_-MBA]_2_PbBr_4_⋅H_2_O (**d**), [R-4-Cl-MBA]_2_PbBr_4_ (**e**), [S-2-Me-BuA]_2_PbBr_4_ (**f**), [S-4-NH_3_-MBA]PbI_4_ (**g**), [R-4-Cl-MBA]_2_PbI_4_ (**h**), and [S-MHA]_2_PbI_4_ (**i**) along select k-paths. Relaxed atomic geometries were used for band structure calculations. Pb-, Br-, I- and organic-derived electronic states are highlighted in purple, green, yellow, and black colors, respectively. Note that the focus of these plots is the degree of SOC near the inorganic-derived conduction band edges, not the alignment of energy levels or the exact fundamental gap.

To investigate the impact of structural distortions on the spin characteristics, we have calculated electronic band structures for the new chiral MHPs, as well as select noncentrosymmetric achiral MHPs in Table 1 (Fig. 3 and Supplementary Figs. 8–13), using DFT and the all-electron electronic structure code FHI-aims^35^ (see Methods for details). We here use the semilocal level of DFT, specifically the Perdew–Burke–Ernzerhof (PBE) functional^36,37^. This choice is appropriate since, in contrast to fundamental gaps or energy level alignments between organic and inorganic components of the MHP, SOC itself is a large effect and already accurately captured at the level of semilocal DFT^37^. Regarding the underlying atomic geometries, band structures of two different structures for each material were considered: First, the experimental room-temperature structures, which average over different thermal motions; second, computationally optimized structures at the DFT-PBE level of theory, amended by the Tkatchenko–Scheffler (TS) van der Waals correction^38^, which correspond to static local minima of the potential energy surface without any averaging over atomic motions. For a consistent comparison, we have aligned the crystal axes for all the MHPs (relaxed and experimental) so that the layer-stacking direction points along the *a*-axis while the *b*- and *c*-axes define the two in-plane directions of the perovskite layer (Fig. 3a). Accordingly, the Γ-X, Γ-Y, and Γ-Z paths in the Brillouin zone coincide, respectively, with *a*-, *b*-, and *c*-directions of perovskite layer in both relaxed and experimental MHP structures (Fig. 3b). In all cases, the calculated frontier CBs and valence bands (VBs) are comprised of inorganic-derived states (Fig. 3d–f), except for [S-4-NO_2_-MBA]_2_PbBr_4_∙H_2_O, wherein the lowest CBs appear to be derived from the organic component (Fig. 3d). The latter band alignment would have to be validated with a level of theory beyond DFT-PBE but does not impact the conclusions for inorganic-derived bands in this work. Importantly, the organic- and inorganic-derived bands are computationally well separated and do not interfere with the analysis of SOC in the inorganic-derived bands.

Figure 3d–i shows the electronic band structures of five new chiral MHPs from Fig. 2, as well as previously reported [R-4-Cl-MBA]_2_PbI_4_ (all corresponding to relaxed geometries). The band structures of [S-4-NO_2_-MBA]_2_PbBr_4_∙H_2_O and [R-4-Cl-MBA]_2_PbBr_4_ exhibit a large spin-splitting in the inorganic-derived frontier CB mainly along the Γ-Y path (Fig. 3d, e). A similar CB spin-splitting occurs in [S-1-1-NEA]_2_PbBr_4_ (Supplementary Fig. 9), as well as in noncentrosymmetric achiral MHPs such as [4AMP]PbBr_4_, [NMA]_2_PbBr_4_, and [PMA]_2_PbCl_4_ (Supplementary Figs. 11–13). As mentioned before, the Γ-Y path coincides with the in-plane *b*-direction of the perovskite layer, along which the local geometry fluctuates due to a large $\Delta \beta$. The 2_1_-screw translational symmetry common to all the MHPs studied here is absent along the *b*-direction (Fig. 2f, g, and Supplementary Fig. 2). Note that, for [R-4-Cl-MBA]_2_PbBr_4_ with a *P*2_1_2_1_2_1_ space group, the 2_1_-screw symmetry along the *b*-direction is for Pb atoms rather than equatorial Br atoms. Along the Γ-Z path that coincides with the in-plane *c*-direction of the perovskite layer, the same type of equatorial halogen atoms and the associated $\beta$ angles propagate by 2_1_-screw translational symmetry (Fig. 2f, g). The lack of band dispersion and spin-splitting along the Γ-X path (i.e., along the layer-stacking direction) follows from the confinement and localization of inorganic-derived states due to organic cations acting as insulating barriers. A large $\Delta \beta$ along the *b*-direction renders the isolated inorganic layers inversion asymmetric (Table 1), thus leading to a strong CB spin-splitting. In contrast with the above MHPs, there is negligible spin-splitting in [S-2-Me-BuA]_2_PbBr_4_, [S-4-NH_3_-MBA]PbI_4_, [R-4-Cl-MBA]_2_PbI_4_, and [S-MHA]_2_PbI_4_ (Fig. 3f–i). Likewise, the noncentrosymmetric achiral lead-iodide MHPs in Table 1 exhibit negligible spin-splitting in their reported band structures^29,30,39^. In these cases, the isolated inorganic layers are nearly centrosymmetric with minimal $\Delta \beta$ (Table 1). A $\Delta \beta$ value of ~6° in [S-MBA]_2_PbI_4_ leads to marginally inversion asymmetric inorganic layers (Table 1), resulting in a CB splitting (Supplementary Fig. 10), which is still 2–4 times smaller compared with the above lead bromide or lead chloride MHPs.

In nonmagnetic solids, the combination of time-reversal and crystal inversion symmetries leads to a twofold spin degeneracy of energy bands. When the crystal inversion symmetry is broken, SOC lifts the spin degeneracy for a general **k**, except at special Kramer points and high-symmetry points, and modifies the dispersion relation of electron/hole bands near the Γ-point to assume the effective form^22,40–42^:

1 $${E}^{\pm }({{{{{\bf{k}}}}}})\,=\,\frac{{{\hslash }}^{2}{{{{k}}}}^{2}}{2m}\,\pm\, {\alpha }_{{{\mathrm{eff}}}}{{{{k}}}}$$

where ${E}^{+}$ and ${E}^{-}$ are the energies of spin-split subbands for the given direction in k-space, and ${\alpha }_{{{\mathrm{eff}}}}\,=\,\Delta {E}^{\pm }/2{{{{{k}}}}}_{0}$ (${{{{{k}}}}}_{0}$ is the characteristic momentum offset) is the effective spin-splitting coefficient (Fig. 3c). For 2D electron gases respecting C_nv_ point group symmetry, Rashba spin-splitting occurs along the in-plane wavevector ${{{{{{\bf{k}}}}}}}_{{{||}}}\,=\,({{{{{{\bf{k}}}}}}}_{{{{{{\bf{x}}}}}}},{{{{{{\bf{k}}}}}}}_{{{{{{\bf{y}}}}}}})$ owing to structural inversion asymmetry perpendicular to the 2D plane (${{{{{{\bf{k}}}}}}}_{{{{{{\boldsymbol{\perp }}}}}}}={{{{{{\bf{k}}}}}}}_{{{{{{\bf{z}}}}}}}$), i.e., along the stacking direction^22^. The high-symmetry C_n_ rotational axis is parallel to ${{{{{{\bf{k}}}}}}}_{{{{{{\bf{z}}}}}}}$, and the twofold spin degeneracy is thus maintained along ${{{{{{\bf{k}}}}}}}_{{{{{{\bf{z}}}}}}}$. In contrast, in the present 2D MHPs for which the local symmetry of the inorganic layer corresponds to either C_2_ or C_2v_ point groups (i.e., bulk inversion asymmetry, BIA), the high-symmetry C_2_ axis coincides with the in-plane Γ-Z path (${k}_{z}$ direction), while the CB spin-splitting occurs dominantly along the other in-plane Γ-Y path (${k}_{y}$ direction) due to significant $\Delta \beta$ (Fig. 3d, e). The much smaller but non-zero spin-splitting in the VB, on the other hand, can be understood from the fact that the VB states are comprised principally from 4p (5p) atomic orbitals of the lighter Br (I) atoms in contrast with the CB states which originate from the 6p orbitals of the heavier Pb atoms (Fig. 3d–i).

**Fig. 4:**
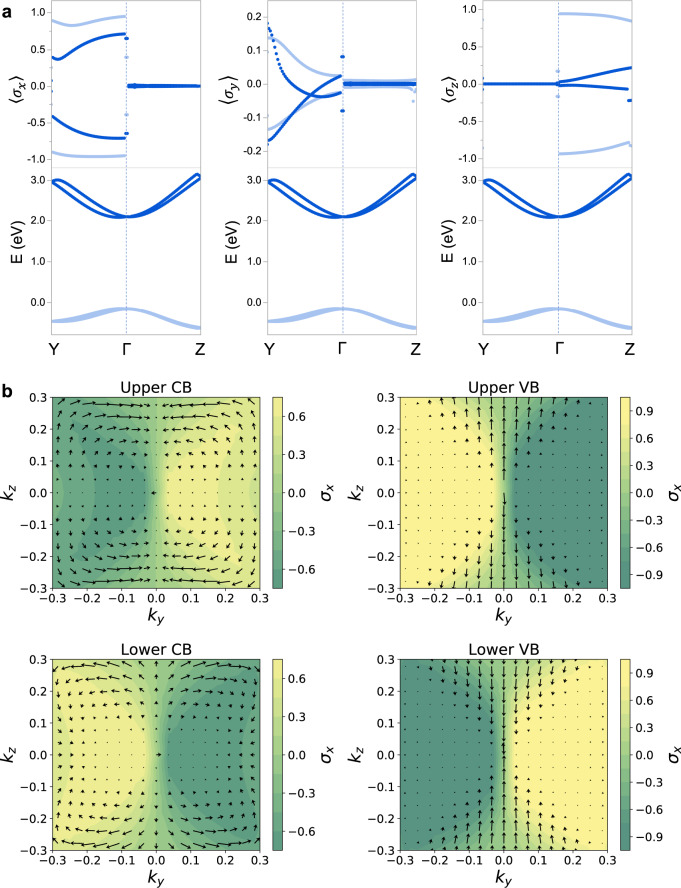
Spin textures in [S-4-NO_2_-MBA]_2_PbBr_4_∙H_2_O. **a** PBE + SOC band structures (bottom panels) and momentum-dependent spin polarization components, $\left\langle {\sigma }_{x}\right\rangle$, $\left\langle {\sigma }_{y}\right\rangle$, and $\left\langle {\sigma }_{z}\right\rangle$ (top panels), shown along the in-plane $\Gamma -Y$ and $\Gamma -Z$ paths. Here, $\left\langle {\sigma }_{y}\right\rangle$ and $\left\langle {\sigma }_{z}\right\rangle$ point along the two in-plane directions of the perovskite layer, while $\left\langle {\sigma }_{x}\right\rangle$ points along the out-of-plane stacking direction. Only frontier conduction (CB) and valence bands (VB) derived from the metal halide framework are shown here. **b** Spin polarization of CB and VB subbands projected onto the reciprocal 2D plane perpendicular to the stacking direction. The arrows represent the in-plane components while the color scheme indicates the magnitude of the out-of-plane component.

The spin polarization ($\left\langle \sigma \right\rangle$) values are calculated from the DFT spinor wave functions as the expectation values of the Pauli spin matrices (${\sigma }_{i}$)^31^. The DFT calculated $\left\langle \sigma \right\rangle$ values for [S-4-NO_2_-MBA]_2_PbBr_4_∙H_2_O with C_2_ point group, as an example, reveal opposite signs of spin polarization for the upper and lower spin-split frontier CBs (derived from the lead bromide framework) along the $\Gamma -Y$ path. Moreover, the out-of-plane $\langle {\sigma }_{x}\rangle$ component is dominant, with the in-plane $\,\langle {\sigma }_{z}\rangle$ and $\langle {\sigma }_{y}\rangle$ components being much smaller along the $\Gamma -Y$ path (Fig. 4a). The spin polarization mapped onto the reciprocal 2D plane of the perovskite layer is essentially characteristic of the Dresselhaus-type spin-texture arising from BIA and captures the spin polarization anisotropy in the spin-split CBs (Fig. 4b). This can be understood from the theory of invariants in conjunction with the strongly anisotropic character of 2D MHPs (see Supplementary Notes 3 and 4). For C_2_ point group, the BIA related Hamiltonian ${H}_{{BIA}}\,$is written near $\Gamma$ to linear order in in-plane wavevector components ${k}_{y}$ and ${k}_{z}$ as:

2 $${H}_{{BIA}}({k}_{y},{k}_{z})\,=\,{\alpha }_{{yy}}{J}_{y}{k}_{y}\,+\,{\alpha }_{{zz}}{J}_{z}{k}_{z}\,+\,{\alpha }_{{xy}}{J}_{x}{k}_{y}$$

where, ${J}_{i}$ denote Pauli operators representing the components of the total angular momentum (note that spin is not a good quantum number due to strong SOC) and ${\alpha }_{{ij}}$ denote band specific SOC coefficients. Upon diagonalizing ${H}_{{BIA}}$, we find the energy correction to the band dispersion as:

3 $${{E}_{{BIA}}}^{\pm }\,=\,\pm \sqrt{{\alpha }_{{xy}}^{2}{k}_{y}^{2}\,+\,{\alpha }_{{yy}}^{2}{k}_{y}^{2}\,+\,{\alpha }_{{zz}}^{2}{k}_{z}^{2}\,}$$

Along the $\Gamma -Y$ and $\Gamma -Z$ paths, this leads to a band splitting of the form in Eq. (1) with the respective effective SOC coefficients of ${\alpha }_{{{\mathrm{eff}}}}\,=\,\sqrt{{\alpha }_{{xy}}^{2}\,+\,{\alpha }_{{yy}}^{2}}\,$and ${\alpha }_{{{\mathrm{eff}}}}\,=\,{\alpha }_{{zz}}$. This band splitting is equivalent to Zeeman splitting under an effective spin–orbit magnetic field, ${{{\mathbf{B}}}}_{{{\mathbf{eff}}}}$^25,28^

4 $${{{\mathbf{B}}}}_{{{\mathbf{eff}}}}\,=\,\frac{1}{{\mu }_{b}}\left\{{\alpha }_{{xy}}{k}_{y}\hat{x}\,+\,{\alpha }_{{yy}}{k}_{y}\hat{y}\,+\,{\alpha }_{{zz}}{k}_{z}\hat{z}\right\}$$

where ${\mu }_{b}$ is the Bohr magneton. Analysis shows that, if ${{\mathbf{{B}}}_{{eff}}}$ is predominantly along one principal axis, the spin polarization is directed parallel or anti-parallel to ${{\mathbf{{B}}}_{{eff}}}$ with a magnitude proportional to the effective g-factor of the Bloch function.

The effective g-factor along a given direction *i* is defined as ${\gamma }_{i}\,=\,\left\langle {\sigma }_{i}\right\rangle /\left\langle {J}_{i}\right\rangle$, where $\left\langle {\sigma }_{i}\right\rangle$ and $\left\langle {J}_{i}\right\rangle$ are the expectation values of the spin and total angular momentum components, respectively. For the spin-split CBs, the effective g-factor is anisotropic owing to associated $P$-type Bloch functions, in conjunction with a strong anisotropy between the in-plane and out-of-plane directions of the lead halide framework. The latter can be described within multiband K.P theory in terms of a crystal field effect that causes mixing of ${P}_{1/ 2}$ and ${P}_{3/ 2}$ CB states. Analysis using tetragonal crystal field parameters (${\sin }\theta$ = 0.2–0.32) previously determined from experiments on a related 2D lead bromide perovskite^43,44^ reveals that the computed effective g-factors are dominant in the out-of-plane direction (${\gamma }_{x}\,=\,-{{\cos }}\,2\theta$), but much smaller in magnitude in the in-plane directions (${\gamma }_{\parallel }\,=\,-{{{\sin }}}^{2}\theta$), explaining why $\langle {\sigma }_{x}\rangle$ >> $\langle {\sigma }_{y}\rangle$ and $\langle {\sigma }_{z}\rangle$ (Fig. 4). For a more detailed discussion, including for the VB, see Supplementary Note 4. ${{\mathbf{{B}}}_{{eff}}}$ has only a *z*-component along ${k}_{z}$, while it has both *x*- and *y*-components along ${k}_{y}$ (Eq. (4)). The dominant CB spin-splitting along the $\Gamma -Y$ path suggests a dominant *x*-component of ${{\mathbf{{B}}}_{{eff}}}$. For a system with C_2v_ point group symmetry with C_2_ axis pointing along ${k}_{z}$, spin-splitting is exclusively along ${k}_{y}$ with *x*-directed ${{\mathbf{{B}}}_{{eff}}}$, while ${k}_{z}$-related terms are strictly forbidden by symmetry (Supplementary Table 7). Therefore, while the local inversion asymmetry (i.e., owing to a large $\Delta \beta$) induces spin-splitting, the global symmetry governs the specifics of k-dependent spin-splitting and spin polarizations. To further exemplify this point, (FC_2_H_4_NH_3_)_2_PbCl_4_ (P*nma* global space group) crystallizes with a center of inversion in-between the two inorganic layers comprising the unit cell^45^. Each inorganic layer is noncentrosymmetric (nominal P*mc*2_1_ layer group) owing to a large $\Delta \beta$. The local inversion asymmetry of the inorganic layer leads to CB splitting, but the global inversion symmetry leads to a zero net spin polarization of the resulting 2-fold degenerate upper and lower CBs (Supplementary Note 5).

**Fig. 5:**
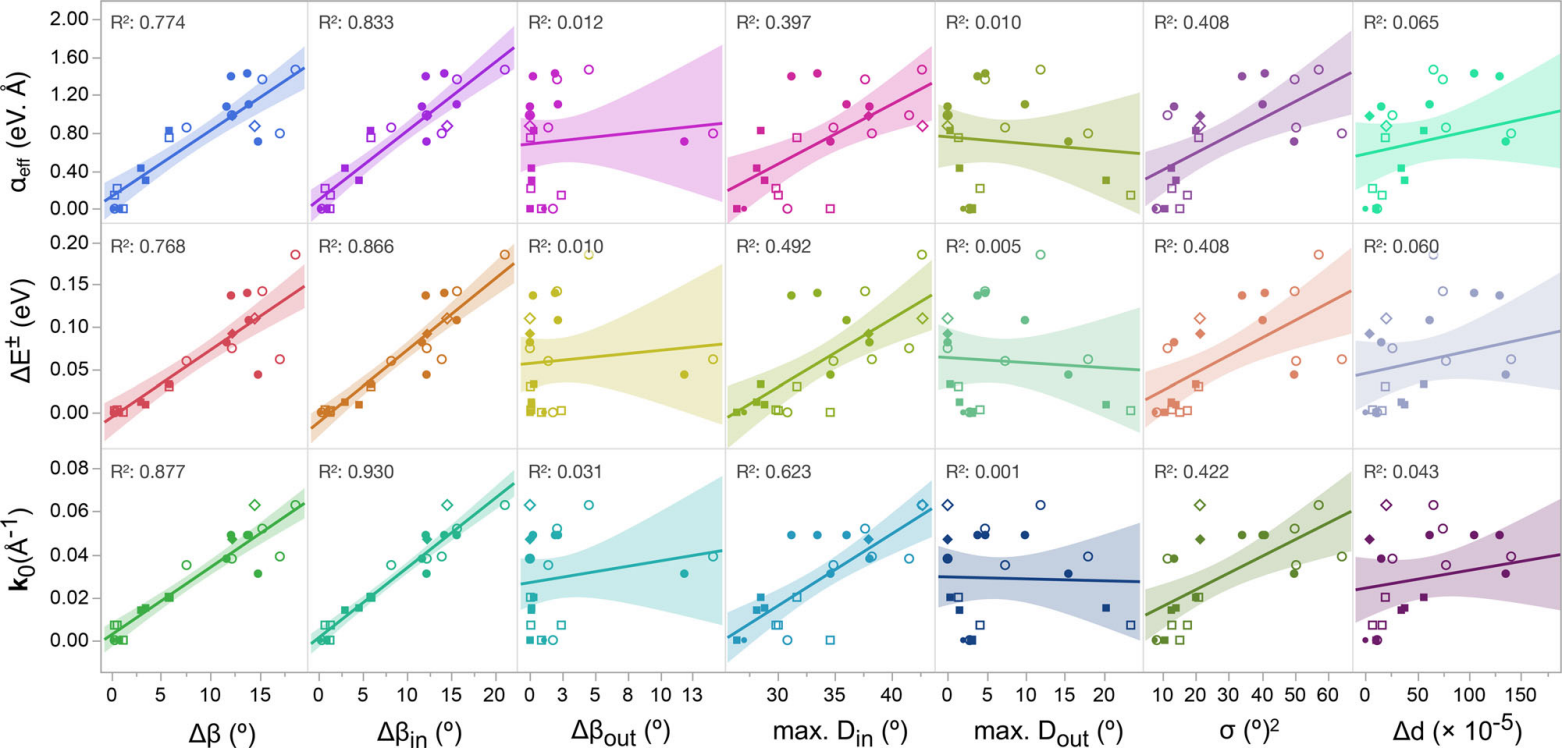
Structural correlations of spin-splitting in 2D MHPs. Correlations between various inorganic layer distortions and predicted spin-splitting parameters ($\Delta {E}^{\pm },{{{{{{\bf{k}}}}}}}_{0}$, and $\alpha_{{{\mathrm{eff}}}}$) in select chiral and achiral MHPs with experimental and with computationally relaxed geometries (Table 1). Data points corresponding to X = Cl, Br, and I are denoted with diamonds, circles, and squares, respectively. The filled and open symbols denote, respectively, the experimental and relaxed MHP atomic configurations. The solid lines are fits to a linear regression model, and the corresponding R^2^ values and 95% confidence intervals (shaded regions) are shown in each subplot. $\Delta \beta$, $\Delta {\beta }_{{in}}$, and $\Delta {\beta }_{{out}}$ denote the asymmetric interoctahedral distortions, whereas $\Delta d$ and ${\sigma }^{2}$ denote the intraoctahedral distortions (see the main text for details). “max. ${D}_{{in}}$” and “max. ${D}_{{out}}$” denote, respectively, the maximum in-plane (${D}_{{in}}\,=\,180^\circ \,-\,{\beta }_{{in}}$) and out-of-plane (${D}_{{out}}\,=\,180^\circ \,-\,{\beta }_{{out}}$) distortion values corresponding to smaller ${\beta }_{{in}}$ and ${\beta }_{{out}}$ angles within the inorganic layer.

To further establish a possible quantitative relationship between spin-splitting and structural characteristics, we plot $\Delta {E}^{\pm }$, ${{{{{\bf{k}}}}}}_{{{{{0}}}}}$, and ${\alpha }_{{{\mathrm{eff}}}}$ estimated for the series of relaxed and unrelaxed MHPs studied here (Fig. 3, Supplementary Figs. 8–13, and Supplementary Table 2) as a function of various distortion metrics (Fig. 5). Remarkably, all three parameters strongly correlate with $\Delta \beta$. Upon decomposing $\beta$ into in-plane (${\beta }_{{in}}$) and out-of-plane (${\beta }_{{out}}$) components (see Supplementary Note 1), the in-plane disparity ($\Delta {\beta }_{{in}}$) values are found to correlate with the spin-splitting parameters most strongly, while there is barely a correlation with the out-of-plane disparity ($\Delta {\beta }_{{out}}$) values. The latter apparent lack of correlation is plausibly because the $\Delta {\beta }_{{out}}$ values are clustered in a low-distortion regime except for [R-4-Cl-MBA]_2_PbBr_4_, which exhibits the largest $\Delta {\beta }_{{out}}$ values (~12° and 14°) found in the series (Fig. 5). Secondary weaker correlations are found with the maximum in-plane distortion (${D}_{{in}}\,=\,180\,-\,{\beta }_{{in}}$) and ${\sigma }^{2}$, since they both are angular distortions related to $\Delta {\beta }_{{in}}$ (Fig. 5). Other distortion parameters correlate less clearly or not at all with computed spin-splitting.

**Fig. 6:**
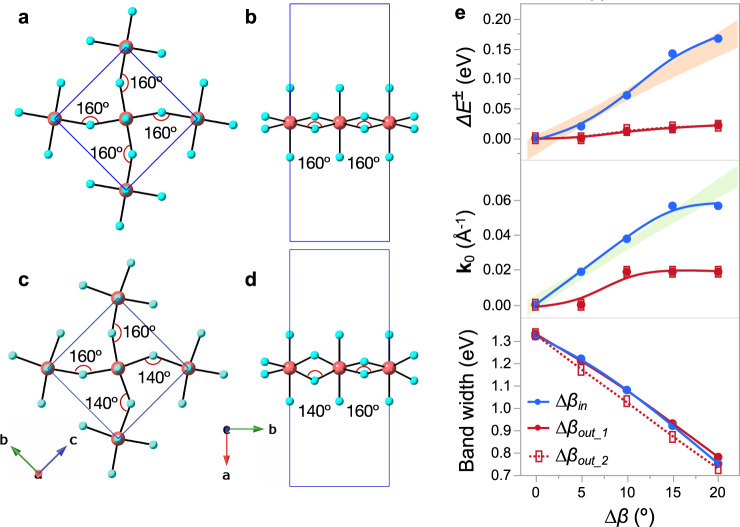
First principles simulations on 2D Cs_2_PbBr_4_ models. **a**, **b** Representative (√2 × √2) − R45° Cs_2_PbBr_4_ models with symmetrical in-plane (**a**) and out-of-plane (**b**) tilting of adjacent PbBr_6_ octahedra, i.e., with $\Delta \beta \,=\,0$. **c**, **d** Representative (√2 × √2) − R45° Cs_2_PbBr_4_ models with asymmetrical in-plane (**c**) and out-of-plane (**d**) tilting of adjacent PbBr_6_ octahedra with $\Delta {\beta }_{{in},{out}}\,=\,20^\circ$. **e** Plots showing the evolution of lowest conduction subband energy width, ${{{{{{\bf{k}}}}}}}_{0}$, and $\Delta {E}^{\pm }$ along the spin-splitting k-path as a function of $\Delta {\beta }_{{in}}$ and $\Delta {\beta }_{{out}}$ in the series of Cs_2_PbBr_4_ models. See Supplementary Fig. 16 and Supplementary Note 2 for details. The legends $\Delta {\beta }_{{out\_}1}$ and $\Delta {\beta }_{{out\_}2}$ in (**e**) correspond to the models with and without formal dipole on the Pb site, respectively. The shaded regions in (**e**) are 95% confidence intervals of fitting as a function of ${\Delta \beta }_{{in}}$ in Fig. 5.

To make the empirical correlation between spin-splitting and $\Delta \beta$ unambiguous, we have performed simulations on idealized 2D Cs_2_PbBr_4_ models using a $(\sqrt{2}\times \sqrt{2})\,-\,R45^\circ$ (per Wood’s notation^46,47^) perovskite lattice, by systematically varying exclusively either $\Delta {\beta }_{{in}}$ or $\Delta {\beta }_{{out}}$ from 0° to 20° along the *b*-direction (Fig. 6, Supplementary Fig. 16, and Supplementary Table 3). Idealized Cs_2_PbX_4_ models have also been used in the past for conceptual studies of structure-property relationships in 2D perovskites^48^. The intraoctahedral distortions ($\Delta d$ and ${\sigma }^{2}$) induced by $\Delta {\beta }_{{in}}$ or $\Delta {\beta }_{{out}}$ are relatively small in the present Cs_2_PbBr_4_ models as compared with the experimental MHPs and are similar between both sets of models, thereby allowing us to isolate and compare the dominant effects of $\Delta {\beta }_{{in}}$ versus $\Delta {\beta }_{{out}}$. The calculated band structures reveal that, while the lowest CB subband energy width along the spin-splitting k-path decreases very similarly with $\Delta {\beta }_{{in}}$ and $\Delta {\beta }_{{out}}$, ${{{\bf{k}}}}_{0}$ and $\Delta {E}^{\pm }$ increase steeply with $\Delta {\beta }_{{in}}$, but less significantly with $\Delta {\beta }_{{out}}$ (for details, see Supplementary Note 2). Both $\Delta {\beta }_{{in}}$ and $\Delta {\beta }_{{out}}$ create an inversion asymmetric local Br environment around Pb, meeting the conditions for the creation of spin-splitting in principle. According to our findings, the quantitative difference of spin-splittings caused by $\Delta {\beta }_{{in}}$ vs. $\Delta {\beta }_{{out}}$ is an intrinsic consequence of the distortion direction.

In Supplementary Tables 4–6, we further investigated a possible correlation between spin-splitting strength and the presence of a formal local dipole (often invoked in model description of spin-splitting) in the structure. Whereas $\Delta {\beta }_{{in}}$ necessarily generates a formal dipole on the Pb site, $\Delta {\beta }_{{out}}$ can be introduced with or without a resulting formal dipole on the Pb site in the idealized Cs_2_PbBr_4_ models (Supplementary Fig. 16 and Supplementary Tables 4–6). Consistently, $\Delta {\beta }_{{in}}$ models exhibit a noncentrosymmetric polar P*mc*2_1_ space group, whereas the $\Delta {\beta }_{{out}}$ models with and without a formal dipole exhibit noncentrosymmetric polar P*ma*2 and nonpolar P222_1_ space groups, respectively (Supplementary Table 3). We find that the spin-splitting associated with $\Delta {\beta }_{{out}}$ does not change significantly regardless of whether a formal local dipole is present in the selected geometry (Fig. 6e and Supplementary Fig. 20). Notably, the strong correlation of spin-splitting parameters with $\Delta {\beta }_{{in}}$ overlaps with the confidence intervals of the fitting shown in Fig. 5, implying that the empirical correlation in observed MHPs is quantitative. This direct structure-property correlation enables a fast route to an informed discovery/screening of promising spin-selective MHPs, simply based on crystal structure information that can be readily accessed in perovskite databases^49^.

## Discussion

Zhang et al. put forth important symmetry criteria based on global space groups and site point groups to observe either conventional or compensated Dresselhaus and/or Rashba effects in nonmagnetic inorganic compounds^50^. In 2D MHPs, however, noncentrosymmetric global space groups often arise from the molecular organic sublattice and do not necessarily imply strong spin-splitting. As the SOC effects are principally derived from the inorganic sublattice, the local distortions and the ensuing local asymmetry within the inorganic layers must underlie and control the spin-splitting in 2D MHPs. Our study establishes a generic local structural descriptor ($\Delta {\beta }_{{in}}$), which signifies the local inversion asymmetry and quantitatively correlates with the DFT-predicted spin-splitting in a broad array of MHPs. Using this single descriptor, one can rapidly screen promising spin-splitting candidates from ever-growing perovskite libraries, with DFT or appropriately parametrized tight binding approaches^51,52^ available for *a posteriori* validation if desired.

Note that low-frequency, high-amplitude optical phonons are known to arise from the lead halide framework below 100 cm^−1^ (<<*k*T at room temperature)^53,54^. The room-temperature experimental X-ray structures, therefore, represent a time average of all possible distortions induced by these soft phonons under thermal equilibrium. Nevertheless, the key structural attribute strongly correlating with the spin-splitting is still $\Delta {\beta }_{{in}}$ (Fig. 5 and Supplementary Table 2). This distortion prevails in the thermal time average structures, apparently connected to symmetry-breaking local minimum-energy structures on the Born–Oppenheimer surface, as corroborated by DFT (Supplementary Table 2). While instantaneous structural fluctuations due to phonon modes may quantitatively increase or decrease the spin-splitting of the adiabatic electronic band structure at any given time, the average spin-splitting is expected to retain a clearly defined trend, imparted by the thermal average structure.

Finally, in the discussed MHP systems, the predicted anisotropic spin polarization of the CB is reminiscent of a persistent spin texture^25,55^, which is posited to enable longer spin lifetimes. For chiral systems, the spin polarization is opposite for opposite organic cation chirality^31^, and the ensuing chiral degrees of freedom coupled with a large spin-splitting predicted for some of the current chiral MHPs represent a promising avenue for spin manipulation in prospective spintronic devices.

## Methods

### Materials

(S)-4-nitro-α-methylbenzylamine hydrochloride [“S-4-NO_2_-MBA∙HCl”, 97%] (also called (S)-α-methyl-4-nitrobenzylamine hydrochloride); (S)-(+)-1-methylhexyamine [“S-MHA”, 99%] (also called (S)-(+)-1-aminoheptane); (S)-(−)-2-methylbutylamine [“S-2-Me-BuA”, 95%]; aq. HI (57 wt. % in H_2_O, distilled, stabilized, 99.95%), aq. HBr (48 wt.% in H_2_O, ≥99.99%) and aq. H_3_PO_2_ (50 wt.% in H_2_O) were purchased from Sigma Aldrich. (R)-4-chloro-α-methylbenzylamine [“R-4-Cl-MBA”, 97%] was purchased from Fischer Scientific.

### Synthesis

Singlecrystals of all the chiral MHPs studied here, except for [S-4-NO_2_-MBA]_2_PbBr_4_·H_2_O and [S-4-NH_3_-MBA]PbI_4_, were grown by slowly cooling over 60 h a hot (95 °C) aq. HI or aq. HBr solution of PbI_2_ or PbBr_2_ and chiral amine in a 1:2 molar ratio in a sealed vial with N_2_. In a typical synthesis, 0.122 mmol of PbI_2_ or PbBr_2_ and 0.244 mmol of neat chiral amine were used. For growing single-crystals of [S-4-NO_2_-MBA]_2_PbBr_4_·H_2_O, a solution of 0.122 mmol of PbBr_2_ and 0.244 mmol of S-4-NO_2_-MBA∙HCl in 0.5 ml aq. HBr and 1.7 ml methanol was slowly evaporated at room temperature in the ambient atmosphere. For growing single-crystals of [S-4-NH_3_-MBA]PbI_4_, a hot solution of 0.245 mmol of PbI_2,_ and 0.49 mmol of S-4-NO_2_-MBA∙HCl in 0.2 ml of aq. HI and 0.1 ml of aq. H_3_PO_2_ was slowly cooled from 85 °C to room temperature over 5 h. The as-obtained single-crystals were filtered, washed thoroughly with diethyl ether, and vacuum-dried.

### Characterization

Single-crystal X-ray diffraction was carried out at room temperature on a Rigaku XtaLAB Synergy-S diffractometer, using Mo-K$\alpha$ radiation ($\lambda$ = 0.710 Å) and X-ray tube operating at 50 kV and 30 mA. Structure solutions were obtained by SHELXS direct methods and refined using the SHELXL least-squares method within the Olex^2^ package. Symmetry analysis for just the inorganic framework/layers (i.e., after excluding the organic cations from the unit cell) was analyzed post refinement using PLATON’s ADDSYM tool^31^. Default values of angle (0.3° for metrical lattice symmetry) and distance (0.25 Å for coinciding atoms for inversion, translational, and rotational symmetry elements) criteria were used in PLATON’s symmetry analysis.

### Computational methods

The all-electron electronic structure code FHI-aims^35^ was used to carry out the DFT calculations. All calculations are based on numeric atom-centered orbital (NAO) basis sets. Calculations were carried out both for unrelaxed experimental geometries, as well as with relaxed geometries (i.e., local minima of the Born–Oppenheimer potential energy surface). Crystal axes for all the MHPs (relaxed and experimental geometries) have been aligned for consistent comparison so that the layer-stacking direction points along the *a*-axis while the *b*- and *c*-axes define the two in-plane directions of the perovskite layer. Relaxed geometries were obtained with the semilocal PBE functional^36^ plus the TS pairwise dispersion scheme for van der Waals (vdW) interactions^38^. FHI-aims “tight” numerical defaults and k-space grid (2 × 4 × 4) were employed (except for [4AMP]PbBr_4_, whose k-space grid was set to (3 × 2 × 5) due to its specific unit cell). DFT-PBE+TS has proven to give a precise description of complex MHPs in the authors’ past work^5,7,8,31,32,56^, with typical deviations between experimental and computational lattice parameters of ~1% or less and with bond angle deviations of a few degrees. Electronic band structures suitable for analysis of the impact of SOC were obtained at the PBE level of theory, with a well-benchmarked second-variational non-self-consistent SOC approach^37^, and employing FHI-aims “tight” numerical defaults and k-space grid (2 × 4 × 4). Spin-texture calculations were carried out as shown in ref. ^31^. For more general band structure characteristics, such as the fundamental gap or energy level alignments between the organic and inorganic components, higher-level methods such as *GW*^57^ or at least hybrid density-functionals^5,7,8,31,56,58,59^ would be required, as was also done in past work by the authors^5,7,8,31,32,56^. However, we here focus exclusively on the SOC effects on the inorganic-related frontier bands in various MHPs, rather than the level-alignment of the organic and inorganic band edges. SOC is a large effect that is qualitatively well captured already at the PBE level of theory, as shown in ref. ^37^. We therefore rely here on the computationally more affordable semilocal DFT-PBE approach as a useful tool for such SO splittings in semiconductors with sufficiently large gaps.

## Supplementary information

Supplementary Information

## Data Availability

Additional data supporting the findings of this work are provided in Supplementary Information. The single-crystal X-ray structures of the new chiral MHPs, namely, [R-4-Cl-MBA]_2_PbBr_4_, [S-1-Me-HA]_2_PbI_4_, [S-4-NO_2_-MBA]_2_PbBr_4_∙H_2_O, [S-2-Me-BuA]_2_PbBr_4_, and [S-4-NH_3_-MBA]PbI_4_ have been deposited in The Cambridge Crystallographic Data Center (CCDC) database under deposition numbers 2095482-2095486. These data can be obtained free of charge via https://www.ccdc.cam.ac.uk/structures/ and also from the Hybrid^3^ perovskite database via https://materials.hybrid3.duke.edu/materials/search using the search terms: “R-4-Cl-MBA2PbBr4”, “S-1-Me-HA2PbI4”, “S-4-NO2-MBA2PbBr4.H2O”, “S-2-Me-BuA2PbBr4”, and “S-4-NH3-MBAPbI_4_”. Atomic coordinates for the experimental and relaxed structures of MHPs and Cs_2_PbBr_4_ models used for band structure calculations in this study have been provided in Supplementary Note 6. Relevant DFT band structure data is available in the NOMAD repository (10.17172/NOMAD/2021.07.17-1). Other relevant data can be obtained from the corresponding authors upon reasonable request.
