# Supplementary material for: Structural descriptor for enhanced spin-splitting in 2D hybrid perovskites
Source: Nat Commun. 2021 Aug 17;12:4982. doi: 10.1038/s41467-021-25149-7 (PMC8371112; doi:10.1038/s41467-021-25149-7)
Supplement: Supplementary file 1 — Supplementary Information [file 41467_2021_25149_MOESM1_ESM.pdf]

Structural descriptor for enhanced spin-splitting in 2D hybrid perovskites

Manoj K. Jana,[1] Ruyi Song,[2] Yi Xie,[1,3] Rundong Zhao,[1,6] Peter C. Sercel,[4,5] Volker Blum[1,2]* and David B. Mitzi[1,2]*

[1]Thomas Lord Department of Mechanical Engineering and Materials Science, Duke University, Durham, NC 27708, USA.

[2]Department of Chemistry, Duke University, Durham, NC 27708, USA.

[3]University Program in Materials Science and Engineering, Duke University, Durham, NC 27708, USA.

[4]Center for Hybrid Organic Inorganic Semiconductors for Energy, Golden, CO, 80401 USA

[5]Department of Applied Physics and Materials Science, California Institute of Technology, Pasadena, CA, USA.

[6]Current affiliation: School of Physics, Beihang University, Beijing 100191, China

**e-mail: david.mitzi@duke.edu; volker.blum@duke.edu*

Contents

Supplementary Note 1: Structural distortions in 2D MHPs S4

Supplementary Note 2: First-principles simulations of 2D Cs_2PbBr_4 models S22

Supplementary Note 3: Analysis of spin-dependent Hamiltonian associated with bulk inversion asymmetry S35

Supplementary Note 4: Analysis of spin dependent splitting and spin polarization for [S-4-NO_2-MBA]$_2PbBr_4\cdot H_2O$ S37

Supplementary Note 5: Conduction band splitting and spin polarization for $(FC_2H_4NH_3)_2PbCl_4$ with a centrosymmetric P*nma* global space group. S45

Supplementary Note 6: Input geometry files S46

Supplementary References S147

Noncentrosymmetric		Centrosymmetric	
PG	SG	PG	SG
C_1	P1	C_i	P-1
C_2	$P2_1$, C2	C_{2h}	$P2_1/a$, $P2_1/b$, $P2_1/c$,
C_s	Pn, Pc, Cn, Cm, Cc, Ic		$P2_1/m$, $P2_1/n$, C2/c, C2/m
C_{2v}	$Pca2_1$, $Cmc2_1$	D_{2h}	Pbca, Pnma, Cmca
D_2	$P2_12_12_1$	D_{4h}	$P4_2/ncm$

Supplementary Figure 1 | Crystal space groups (SGs) and point groups (PGs) observed frequently among known 2D MHPs. 2D chiral MHPs crystallize in Sohncke groups denoted in grey.

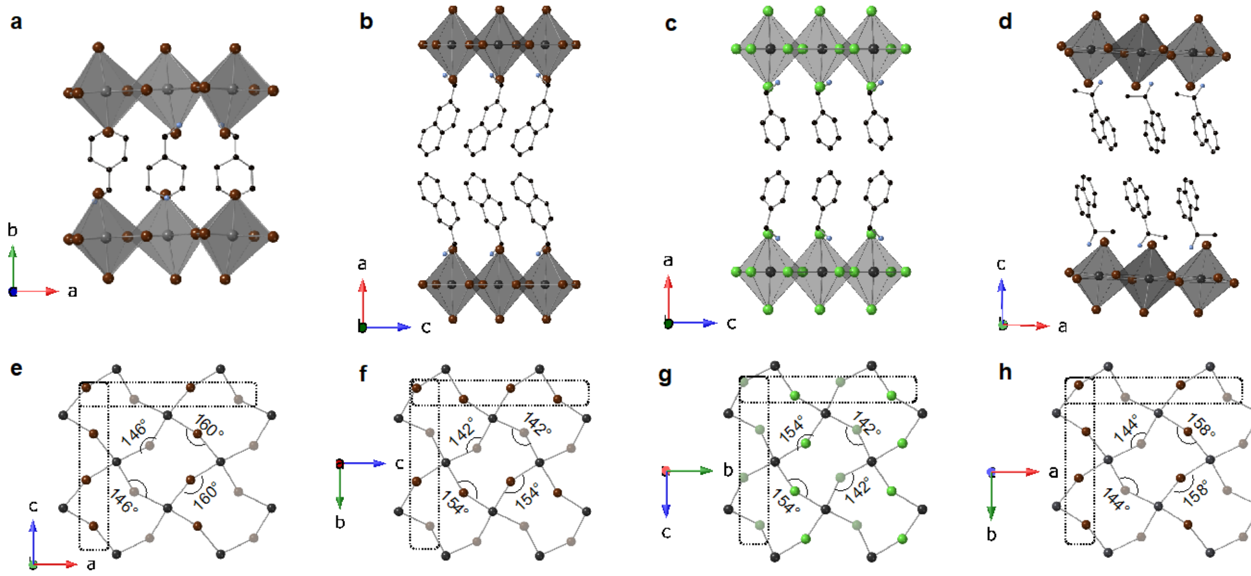

Supplementary Figure 2 | a-d, Schematic single-crystal X-ray structures of (**a**) [4-AMP]$PbBr_4$, (4AMP: 4-(aminomethyl)piperidine), (**b**) $[NMA]_2PbBr_4$ (NMA: 1-(2-naphthyl)methanammonium), (**c**) $[PMA]_2PbCl_4$ (PMA: phenylmethylammonium), (**d**) [S-1-1-$NEA]_2PbBr_4$ (S-1-1-NEA : S-(−)-1-(1-naphthyl)ethylammonium). In each case, all H atoms are omitted for visual clarity. **e-h**, In-plane views of perovskite layers corresponding to panels (**a-d**) showing the equatorial Pb-X-Pb bond angles (β). Axial X atoms are omitted for clarity. The two distinct equatorial X atoms associated with widely disparate β angles are distinguished by shaded and solid spheres. The dashed rectangles differentiate the individual rows comprised of the same type of equatorial X atoms from the rows comprising alternating types of equatorial X atoms, highlighting the local geometry fluctuation along one of the in-plane directions. Grey, brown, purple, green, blue, black, and pink spheres denote Pb, Br, I, Cl, N, C, and H atoms, respectively.

Supplementary Note 1: Structural distortions in 2D MHPs

In 3D AMX_3 perovskite structures (where A=$CH_3NH_3^+$, $CH(NH_2)NH_2^+$, Cs^+, Rb^+; M=Pb^{2+}, Sn^{2+}, Ge^{2+}; X=Cl^-, Br^-, I^-), the corner-sharing MX_6 octahedra create cuboctahedral voids that host the A-site cations. The maximum allowed radius of the A-site cation is 2.60 Å in the largest possible scenario where M=Pb^{2+} and X=I^-, according to the Goldschmidt tolerance criterion for a perfect cubic perovskite.[1,2] With larger cations, the 3D perovskite structure collapses to low-dimensional variants. ⟨100⟩-oriented 2D MHPs with general formula $A'_2A_{n-1}B_nX_{3n+1}$ are conceptually obtained by excising the parent 3D AMX_3 every n^{th} plane along ⟨100⟩-direction and incorporating the larger A' spacer cations in-between the resulting slabs.[2] Here, we focus on the single-layer (n=1) members of 2D MHPs (M=Pb^{2+}) wherein each anionic perovskite layer of corner-sharing $[MX_4]^{2-}$ octahedra alternates with a bilayer (monolayer) of A'^+ (A'^{2+}) organic cations, coupled with specific hydrogen bonding interactions between the organic ammonium head groups and the nearest halogen atoms.

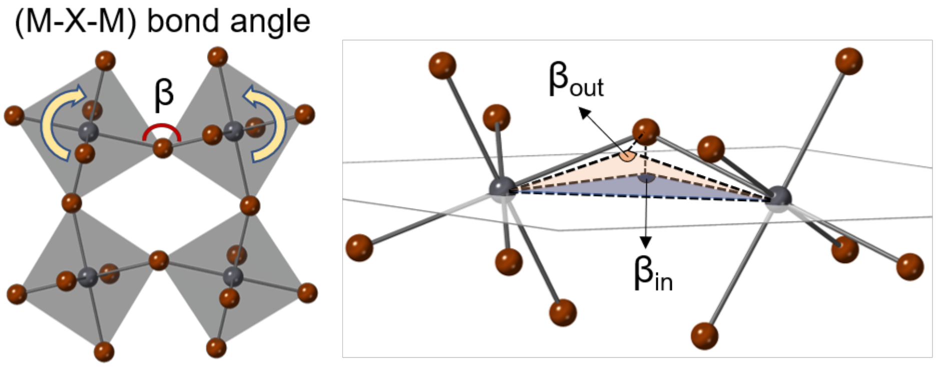

Supplementary Figure 3 | Schematic (left) showing a tilting distortion of corner-sharing MX_6 octahedra in ⟨100⟩-oriented MHPs, that leads to an equatorial (bridging) M-X-M bond angle (β) of < 180º. Schematic (right) showing the in-plane (β_{in}) and out-of-plane (β_{out}) components of β, which are determined by projecting the equatorial X atom onto a horizontal plane (blue) formed by coplanar M atoms, and onto a plane (yellow) perpendicular to the horizontal plane, respectively.

a) Interoctahedral distortions: The allowed cross-sectional area of the organic cation in 2D MHPs is given by the maximum area enclosed by the four axial halogens of neighboring corner-sharing MX_6 octahedra (~ 40 Å^2, for example, in 2D lead iodide perovskites[1,2]). As the cross-sectional area of the organic cation increases, the MX_6 octahedra will tilt to accommodate the

larger cation and allow for an optimal packing of organic cations to meet the charge-density requirements, and such tilting of octahedra causes equatorial (bridging) M-X-M bond angles (β) to decrease from the ideal (undistorted structure) 180° value (Supplementary Figure 3). The in-plane tilting component (β_{in}) is determined by projecting the equatorial X atom onto the horizontal plane consisting of M atoms. The out-of-plane tilting component (β_{out}) is found by projecting the equatorial X atom onto a plane normal to the horizontal plane.[3]

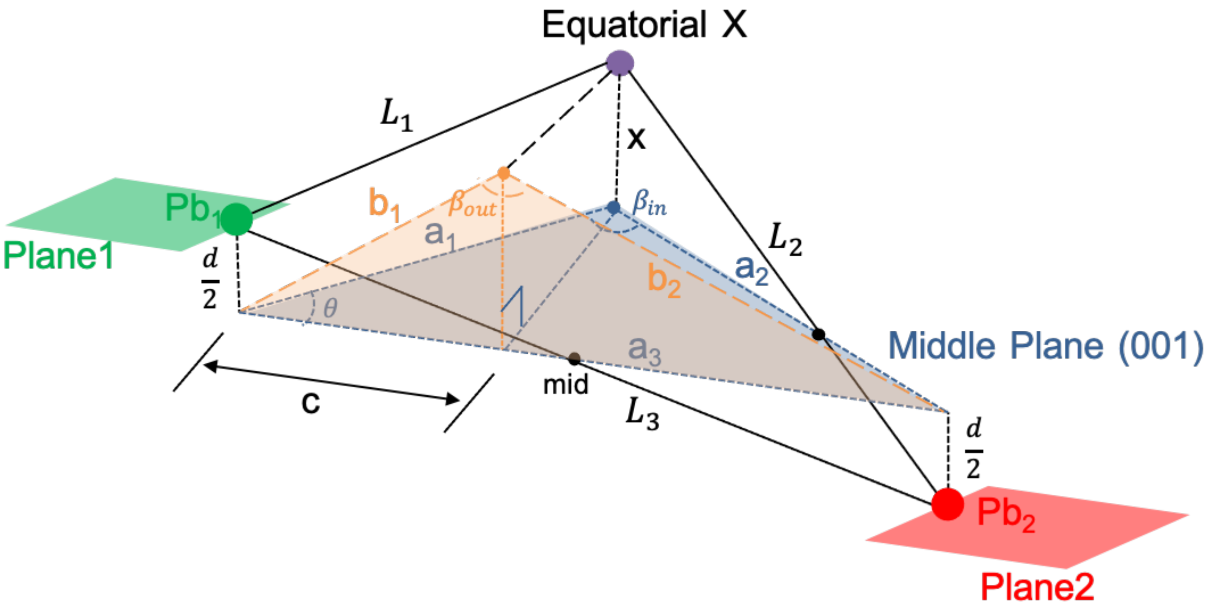

Supplementary Figure 4 | Schematic showing the displacement of adjacent Pb atoms (denoted as Pb_1 and Pb_2) with respect to (100) plane (blue), and the various parameters used for calculating the in-plane (β_{in}) and out-of-plane (β_{in}) components of octahedral tilting distortions.

In some of the lead perovskites studied here, the adjacent Pb atoms are not strictly coplanar, but are slightly displaced by the same distance in opposite directions with respect to the horizontal crystallographic (100) plane. In this case, to accurately determine β_{in} and β_{out}, a triangle is formed within the (100) plane by projecting the two adjacent Pb atoms (denoted as Pb_1 and Pb_2) and the equatorial X atom onto the (100) plane as shown in Supplementary Figure 4. a_1, a_2, and a_3 denote the three lengths of the as-formed triangle, which are mathematically expressed as:

$$a_1 = \sqrt{{L_1}^2 - (x - \frac{d}{2})^2}$$

$$a_2 = \sqrt{{L_2}^2 - (x + \frac{d}{2})^2}$$

$$a_3 = 2 \times \sqrt{(\frac{L_3}{2})^2 - (\frac{d}{2})^2} = \sqrt{{L_3}^2 - d^2}$$

where L_1, L_2, and L_3 denote the distances connecting Pb_1, Pb_2 and X, d is the vertical distance between Pb_1 and Pb_2, and x is the perpendicular distance between equatorial X and the (100) plane (Supplementary Figure 4). Using a_1, a_2 and a_3, β_{in} can be calculated as follows:

$$\beta_{in} = \arccos\left(\frac{\sqrt{{L_1}^2 - (x - \frac{d}{2})^2}^2 + \sqrt{{L_2}^2 - (x + \frac{d}{2})^2}^2 - \sqrt{{L_3}^2 - d^2}^2}{2\sqrt{{L_1}^2 - (x - \frac{d}{2})^2}\sqrt{{L_2}^2 - (x + \frac{d}{2})^2}}\right)$$

$$\beta_{in} = \arccos\left(\frac{{L_1}^2 + {L_2}^2 - {L_3}^2 - 2x^2 + \frac{d^2}{2}}{2\sqrt{{L_1}^2 - (x - \frac{d}{2})^2}\sqrt{{L_2}^2 - (x + \frac{d}{2})^2}}\right)$$

β_{out} can be calculated by projecting X onto a plane (yellow) perpendicular to (100) and forming a triangle with the (100)-plane projected points of Pb_1 and Pb_2. b_1, b_2, and a_3 are the three lengths of this triangle sides (Supplementary Figure 4), which can be derived as follows:

$$\theta = \arccos\left(\frac{\sqrt{{L_1}^2 - (x - \frac{d}{2})^2}^2 + \sqrt{{L_3}^2 - d^2}^2 - \sqrt{{L_2}^2 - (x + \frac{d}{2})^2}^2}{2\sqrt{{L_1}^2 - (x - \frac{d}{2})^2}\sqrt{{L_3}^2 - d^2}}\right)$$

$$\theta = \arccos\left(\frac{{L_1}^2 + {L_3}^2 - {L_2}^2 + 2dx - d^2}{2\sqrt{{L_1}^2 - (x - \frac{d}{2})^2}\sqrt{{L_3}^2 - d^2}}\right)$$

$$c = a_1 \times \cos\theta$$

$$c = \sqrt{{L_1}^2 - (x - \frac{d}{2})^2} \times \frac{{L_1}^2 + {L_3}^2 - {L_2}^2 + 2dx - d^2}{2\sqrt{{L_1}^2 - (x - \frac{d}{2})^2}\sqrt{{L_3}^2 - d^2}}$$

$$c = \frac{{L_1}^2 + {L_3}^2 - {L_2}^2 + 2dx - d^2}{2\sqrt{{L_3}^2 - d^2}}$$

$$b_1 = \sqrt{c^2 + x^2}$$

$$b_1 = \sqrt{\left(\frac{{L_1}^2 + {L_3}^2 - {L_2}^2 + 2dx - d^2}{2\sqrt{{L_3}^2 - d^2}}\right)^2 + x^2}$$

$$b_2 = \sqrt{(a_3 - c)^2 + x^2}$$

$$b_2 = \sqrt{\left(a_3 - \frac{{L_1}^2 + {L_3}^2 - {L_2}^2 + 2dx - d^2}{2\sqrt{{L_3}^2 - d^2}}\right)^2 + x^2}$$

Using b_1 and b_2, β_{out} can be calculated as follows:

$$\beta_{out} = \arccos\left(\frac{\sqrt{\left(\frac{{L_1}^2 + {L_3}^2 - {L_2}^2 + 2dx - d^2}{2\sqrt{{L_3}^2 - d^2}}\right)^2 + x^2}^{\,2} + \sqrt{\left(a_3 - \frac{{L_1}^2 + {L_3}^2 - {L_2}^2 + 2dx - d^2}{2\sqrt{{L_3}^2 - d^2}}\right)^2 + x^2}^{\,2} - {a_3}^2}{2\sqrt{\left(\frac{{L_1}^2 + {L_3}^2 - {L_2}^2 + 2dx - d^2}{2\sqrt{{L_3}^2 - d^2}}\right)^2 + x^2}\sqrt{\left(a_3 - \frac{{L_1}^2 + {L_3}^2 - {L_2}^2 + 2dx - d^2}{2\sqrt{{L_3}^2 - d^2}}\right)^2 + x^2}}\right)$$

$$\beta_{out} = \arccos\left(\frac{2(c^2 + x^2 - a_3 c)}{2\sqrt{c^2 + x^2}\sqrt{(a_3 - c)^2 + x^2}}\right)$$

where

$$a_3 = \sqrt{{L_3}^2 - d^2}$$

$$c = \frac{{L_1}^2 + {L_3}^2 - {L_2}^2 + 2dx - d^2}{2\sqrt{{L_3}^2 - d^2}}$$

Note that when the adjacent Pb atoms are coplanar (i.e., d =0), the picture is simplified to the one shown in Supplementary Figure 3 (right panel). For the MHPs studied here, d is either equal or very close to zero.

b) Intraoctahedral distortions: Intraoctahedral distortion within a single MX_6 octahedron, on the other hand, is quantified using metrics such as bond length distortion, $\Delta d = \left(\frac{1}{6}\right)\Sigma(d_i - d_0)^2/{d_0}^2$ (where d_i denotes the six M-X bond lengths and d_0 is the mean M-X bond length), and bond angle variance, $\sigma^2 = \Sigma_{i=1}^{12}(\theta_i - 90)^2/11$ (where θ_i denotes the individual *cis* X-M-X bond angles).[4]

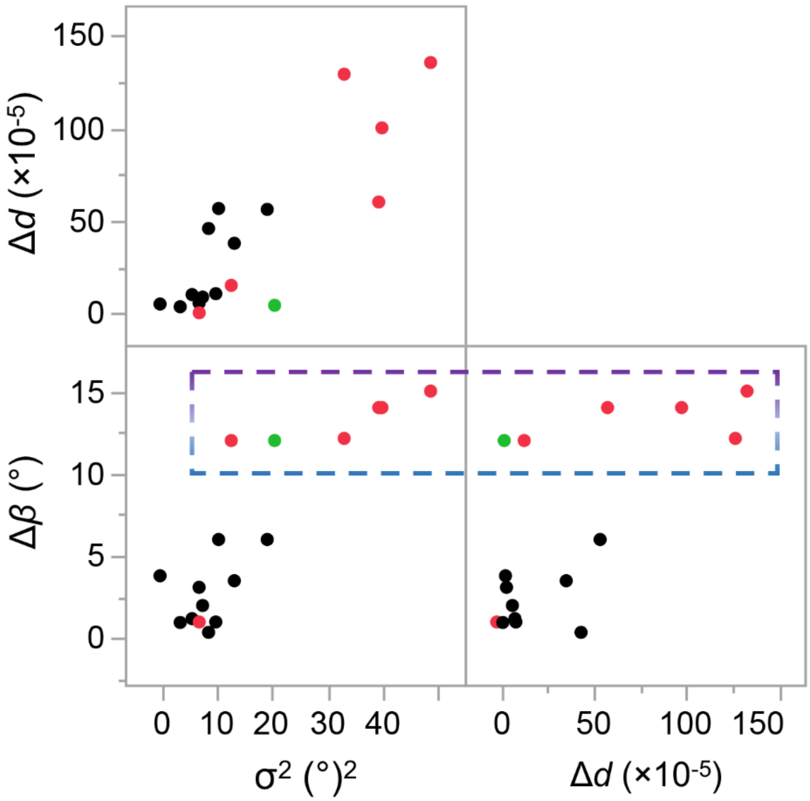

Supplementary Figure 5 | Scatterplot matrix of intraoctahedral distortions (Δd and σ^2) and interoctahedral tilting distortion ($\Delta\beta$), corresponding to X-ray single-crystal structures of chiral and noncentrosymmetric MHPs listed in Table 1 in the main text. Black, red, and green data points denote, respectively, lead iodide, lead bromide, and lead chloride MHPs. The dashed rectangle encompasses MHPs (based on X = Br, Cl) with a large $\Delta\beta$ (> 11º), all of which also exhibit a sizeable spin-splitting (see Supplementary Figures 8-13).

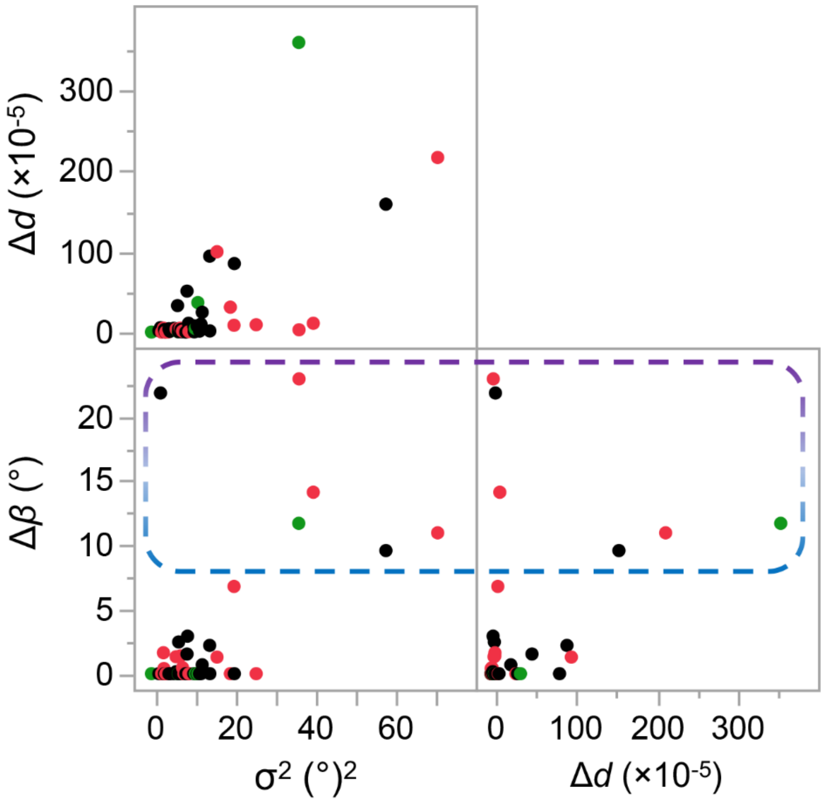

Supplementary Figure 6 | Scatterplot matrix of intraoctahedral distortions (Δd and σ^2) and interoctahedral tilting distortion ($\Delta\beta$), corresponding to X-ray single-crystal structures of > 70 MHPs with centrosymmetric global space groups. Black, red, and green data points denote, respectively, lead iodide, lead bromide, and lead chloride MHPs. The dashed rectangle encompasses MHPs with a large $\Delta\beta$ (> 10º). See **Supplementary Table 1** for the list of MHPs, the corresponding values of distortions, and space groups.

Supplementary Table 1 | Survey of inter- and intra-octahedral distortions in known 2D ⟨100⟩-oriented $[PbX_4]^{2-}$ based perovskites with centrosymmetric global space groups. Crystal structure data were obtained at ambient temperatures unless otherwise mentioned in parentheses.

Organic spacer cation	X	Space group	Intraoctahderal distortions[a]		Interoctahedral distortions		Ref.
			Δd ($\times 10^{-5}$)	σ^2 $(°)^2$	β, β' (°)	$\Delta\beta$ (°)	
2-naphthylmethylammonium (NMA)	Cl	*Pbam*	0.12	0.03	148.02	0	5
**2-fluoroethylammonium (173K)** [b]	Cl	*Pnma*	360	36.94	177.60<br>165.90	11.70	6
cyclopropylammonium (173K)	Cl	$P2_1/c$	0.55	6.73	146.71	0	7
cyclobutylammonium (173K)	Cl	$P2_1/c$	4.35	11.02	148.23	0	7
cyclopentylammonium (173K)	Cl	*Cmca*	36.70	11.66	154.81	0	7
3-(2-ammonioethyl)anilinium (AEA)	Br	$P2_1/c$	8.52	20.72	144.33<br>151.12	6.79	3
1, 4-butanediammonium (BDA)	Br	*P-1*	4.81	7.27	148.41<br>149.79	1.38	3
**histammonium (HIS) (100K)** [c]	Br	$P2_1/c$	2.92	37.01	173.26<br>150.32	22.94	3
2-methyl-1,5-pentanediammonium (MPenDA)	Br	*C2/c*	0.04	2.58	147.40	0	3
1,8-diaminooctane (OdA)	Br	$P2_1/c$	0.07	3.60	148.21	0	3
n-butylammonium (BA)	Br	*Pbca*	0.67	9.42	154.82	0	3
phenethylammonium (PEA)	Br	*P-1*	99.90	16.46	150.82<br>152.11	1.29	8
**3-(dimethylamino)-1-propylamine (DMAPA)** [c]	Br	$P2_1/c$	11	40.58	170.90<br>156.78	14.12	9
1-(1-naphthyl)ethylammonium (Rac-NEA)	Br	$P2_1/c$	31	19.80	152.53	0	10
N^1-methylpropane-1,3-diammonium (NMPDA) (100K)	Br	$P2_1/c$	4.92	3.03	164.27<br>162.64	1.63	11
cyclopropylammonium (173K)	Br	$P2_1/c$	0.16	4.24	146.29	0	7

cyclobutylammonium (173K)	Br	$P2_1/c$	2.10	8.46	148.60	0	7
cyclopentylammonium (173K)	Br	$P2_1/c$	9.20	26.27	153.60	0	7
3,4-dichlorobutan-1-ammonium ($BEA-Cl_2$) (100K)	Br	*P*-1	1.26	7.81	145.41<br>145.88	0.47	12
3,4-dibromobutan-1-ammonium ($BEA-Br_2$) (100K)	Br	*P*-1	3.87	6.21	149.12<br>147.80	1.32	12
but-3-en-1-ammonium (BEA)	Br	$P2_1/c$	0.29	3.20	151.01	0	13
but-3-yn-1-ammonium (BYA) (100K)	Br	$P2_1/c$	1.30	8.17	145.91	0	13
(E)-3,4-diiodobut-3-en-1-ammonium ($BYA-I_2$) (100K)	Br	*P*-1	0.28	3.16	146.44<br>146.04	0.40	12
**N,N-dimethylphenylene-p-diammonium (DPDA) (100K)** [c]	Br	$P2_1/n$	217	71.73	176.88<br>165.92	10.96	14
benzylammonium (BzA)	I	*Pbca*	1.28	12.08	158.43	0	15
4-iodobenzylammonium (4-I-BzA) (100K)	I	$P2_1/c$	3.07	10.81	156.11	0	16
4-fluorobenzylammonium (4-F-BzA) (106K)	I	$P2_1/n$	24.70	12.75	158<br>158.69	0.69	16
2-(1-cyclohexenyl)ethylammonium (173K)	I	*P*-1	94.4	14.62	148.72<br>150.92	2.20	17
1,4-bis-(ammoniomethyl)cyclohexane (173K)	I	$P2_1/c$	3.42	7.45	153.14	0	18
2-ethanolammonium (173K)	I	$P2_1/c$	4.77	2.37	159.10	0	19
3-propanolammonium (173K)	I	$P2_1/c$	33	6.57	163.68	0	19
2-bromoethylammonium (173K)	I	*Pnma*	51	8.94	177.23<br>175.70	1.53	19
2-iodoethylammonium (173K)	I	$P2_1/c$	4.16	4.41	147.25	0	19
3-iodopropylammonium (173K)	I	$P2_1/c$	2.59	12.17	148.76	0	19
4-iodobutylammonium (173K)	I	$P2_1/c$	0.36	10.77	147.02	0	19
5-iodopentylammonium (173K)	I	$P2_1/c$	1.64	8.99	154.36	0	19
6-iodohexylammonium (173K)	I	*Pbca*	10	12.43	160.98	0	19
5,5‘-bis(ammoniumethylsulfanyl)-2,2‘-bithiophene (BAEST)	I	*P*-1	4.23	6.85	149.40<br>151.87	2.47	20

1-methylbutylammonium (1-Me-ba)	I	$P2_1/c$	7.63	11.46	153.90	0	21
1-methylhexylammonium (1-Me-ha)	I	$P2_1/c$	7.87	10.63	153.87	0	21
**1-methylpropylammonium (1-Me-pa)** [c]	I	$P4_2/ncm$	5.58	2.3	180<br>158.16	21.84	21
2-ethylhexylammonium (2-Et-ha)	I	$P2_1/c$	1.06	4.67	153.98	0	21
cyclopropylammonium (173K)	I	$P2_1/c$	1.79	1.89	147.16	0	22
cyclobutylammonium (173K)	I	$P2_1/c$	0.52	6.82	147.27	0	22
cyclopentylammonium (173K)	I	$P2_1/c$	4.72	5.61	154.57	0	22
cyclohexylammonium (173K)	I	*Pbca*	11	9.33	154.79	0	22
dodecylammonium (DA)	I	$P2_1/a$	85.20	20.80	150.67	0	23
1,6-diaminohexane (HdA) (200K)	I	$P2_1/c$	3.27	3.30	148.31	0	24
1,8-diaminooctane (OdA) (200K)	I	$P2_1/c$	0.42	7.68	147.45	0	24
benzodiimidazolium (Bdi)	I	$C2/m$	0.02	8.63	180	0	25
naphthalene-O-propylammonium	I	$P2_1/c$	1.49	14.67	146.87	0	26
naphthalene-O-hexylammonium	I	$P2_1/c$	2.65	9.07	146.14<br>149.06<br>148.60	2.92	27
MeO-naphthalene-O- ethylammonium	I	*Pbca*	0.06	4.79	155.56	0	27
MeO-naphthalene-O-hexylammonium	I	*P*-1	2.14	6.20	154.46<br>154.31	0.15	27
perylene-O-ethylammonium	I	$P2_1/c$	0.15	3.47	155.23	0	26
**N,N-dimethylphenylene-p-diammonium (DPDA) (100K)** [c]	I	$P2_1/n$	159	58.77	176.64<br>167.06	9.58	14

[a] Average values are reported.
[b] In this MHP, $\Delta\beta$ = 11.7° along one in-plane direction and $\Delta\beta$ = 0º along the other in-plane direction similar to chiral MHPs in Fig. 2f, g in the main text; the isolated inorganic layers are nominally noncentrosymmetric (see **Supplementary Figure 7a**)
[c] These MHPs exhibit $\Delta\beta > 9°$; however, equal β angles are found on opposite sides of the squares formed by Pb atoms in the structure so that an inversion center is retained (see **Supplementary Figures 7b and c**).

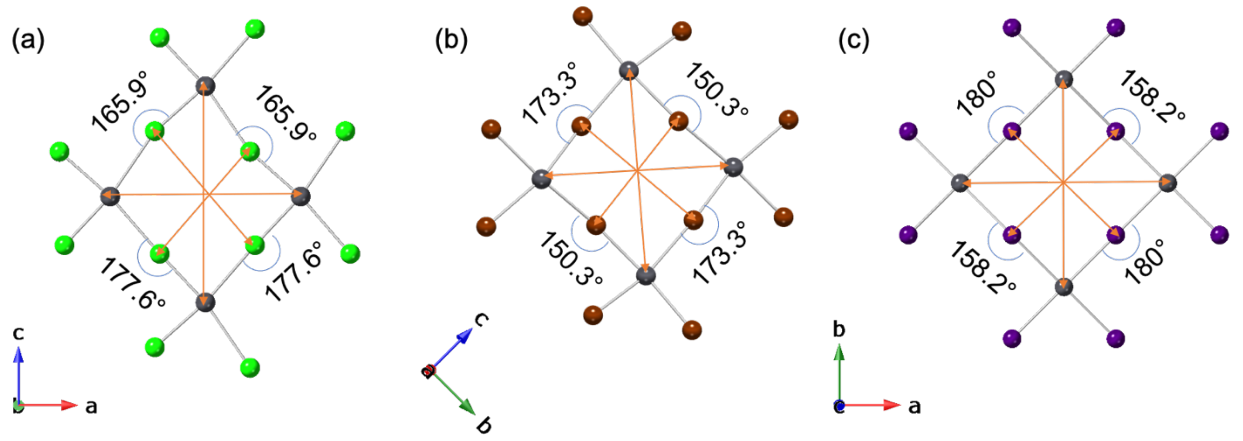

Supplementary Figure 7 | a-c, In-plane views of perovskite layers in (**a**) 2-fluoroethylammonium lead chloride (centrosymmetric, *Pnma*), (**b**) histammonium lead bromide (centrosymmetric, $P2_1/c$) and (**c**) 1-methylpropylammoium lead iodide (centrosymmetric, $P4_2/ncm$). The equatorial Pb-X-Pb bond angles, β, are shown in each panel. The layers shown in **(b)** and **(c)** possess inversion symmetry. The layer shown in **(a)** does not have inversion symmetry; however, there is an inversion center between adjacent inorganic layers in this structure.

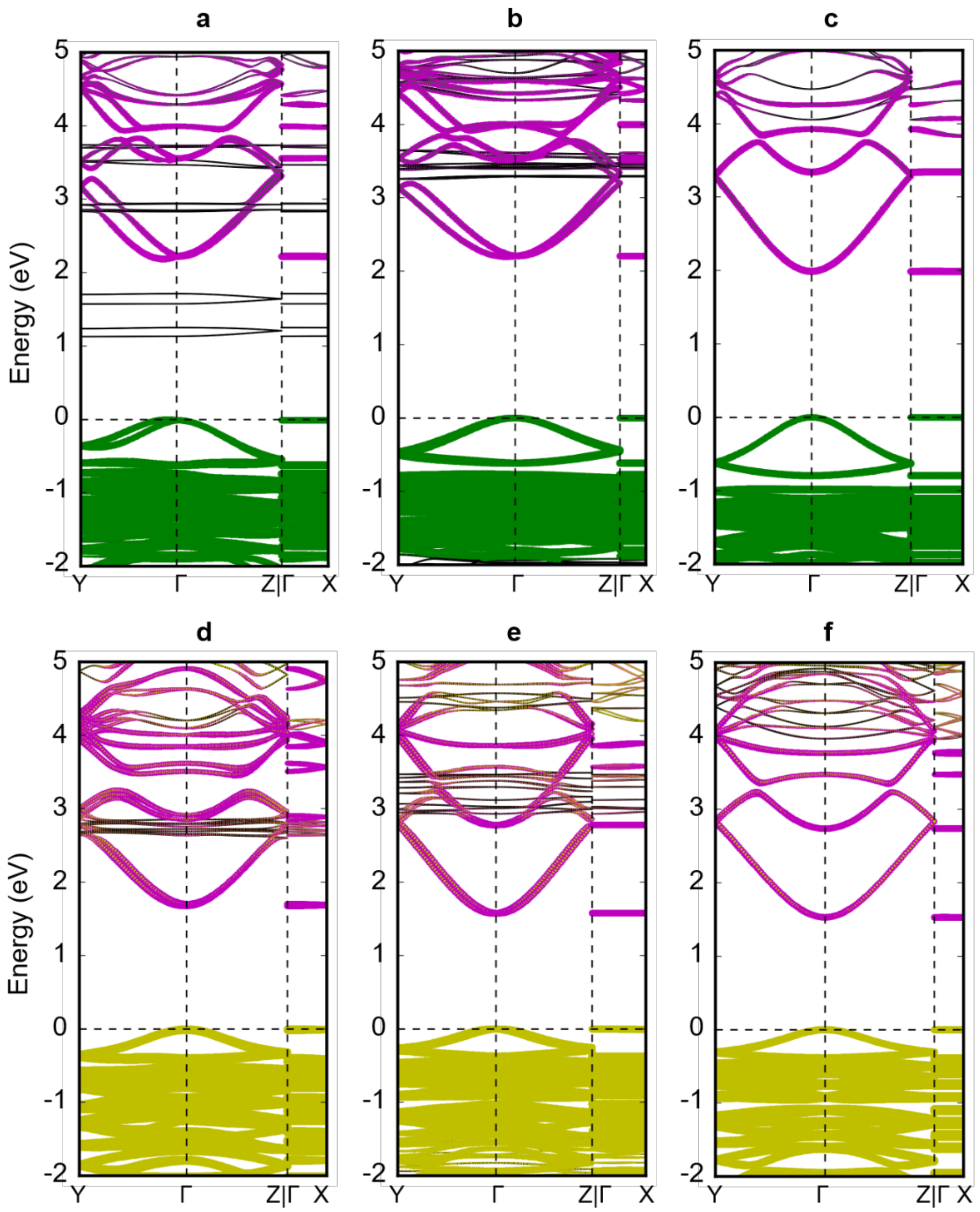

Supplementary Figure 8 | **a-f,** DFT-PBE+SOC electronic band structures of chiral [S-4-NO_2-MBA]$_2$PbBr_4·H_2O (**a**), [R-4-Cl-MBA]$_2$PbBr_4 (**b**), [S-2-Me-BuA]$_2$PbBr_4 (**c**), [S-4-NH_3-MBA] PbI_4 (**d**), [R-4-Cl-MBA]$_2$PbI_4 (**e**), and [S-MHA]$_2$PbI_4 (**f**) along the k-paths shown in Figure **3b** of the main text. Experimental atomic coordinates were used for band structure calculations. Pb-, Br-, I- and organic-derived electronic states are highlighted in purple, green, yellow, and black colors, respectively. Note that the focus of these plots is the degree of SOC near the inorganic-derived conduction band edges (which is known to be captured faithfully already at the DFT-PBE+SOC

level of theory), not the alignment of energy levels or the exact fundamental gap. In preparing **Supplementary Figures 8-13**, attention was paid to relative energy band structure shifts between experimentally derived and computationally optimized geometries. The observed band structure differences can be traced to seemingly small changes in geometry between experiment and computation. For example, small experimental (XRD) uncertainties in bond lengths associated with light-element aromatic rings can translate into noticeable changes of the molecular electronic structure and also shifts with respect to the inorganic-derived bands. The key point of this paper, which is the correlation between observed Rashba-Dresselhaus spin splitting and inorganic layer geometry, remains unaffected by these differences.

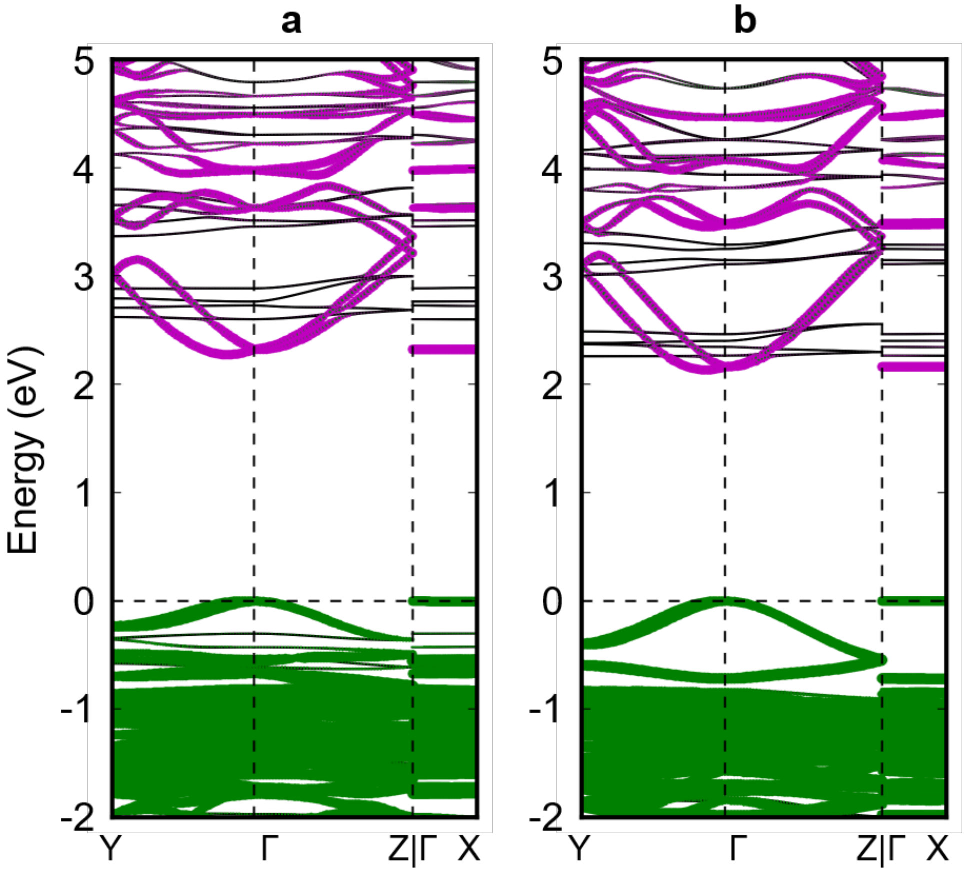

Supplementary Figure 9 | **a,b,** DFT-PBE+SOC electronic band structures of chiral [S-1-1-NEA]$_2$PbBr$_4$ using computationally relaxed (**a**) and experimental (**b**) atomic coordinates. Pb-, Br-, and organic-derived electronic states are highlighted in purple, green, and black colors, respectively. Note that the focus of these plots is the degree of SOC near the inorganic-derived conduction band edges (which is known to be captured faithfully already at the DFT-PBE+SOC level of theory), not the alignment of energy levels or the exact fundamental gap. See caption of **Supplementary Figure 8** for further comments regarding observed band structure changes related to experimental and computational geometries.

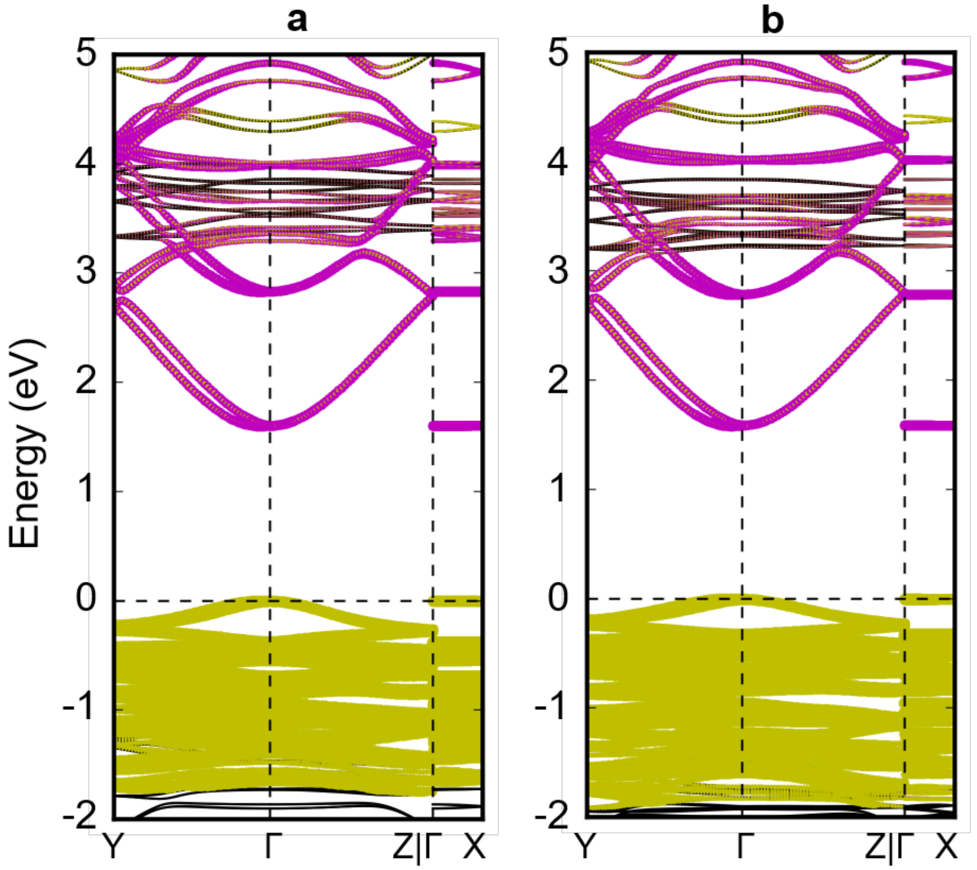

Supplementary Figure 10 | **a,b,** DFT-PBE+SOC electronic band structures of chiral [S-MBA]$_2$PbI$_4$ using computationally relaxed (**a**) and experimental (**b**) atomic coordinates. Pb-, I-, and organic-derived electronic states are highlighted in purple, yellow, and black colors, respectively. Note that the focus of these plots is the degree of SOC near the inorganic-derived conduction band edges, not the alignment of energy levels or the exact fundamental gap. See caption of **Supplementary Figure 8** for further comments regarding observed band structure changes related to experimental and computational geometries.

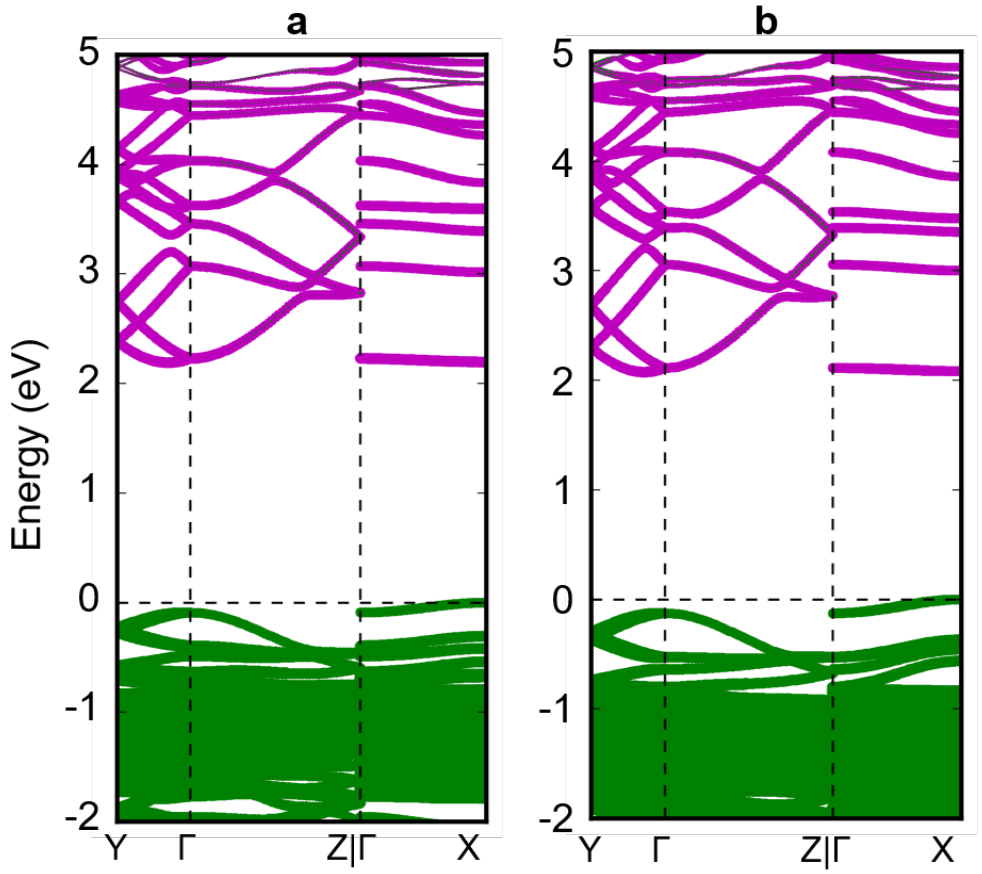

Supplementary Figure 11 | a,b, DFT-PBE+SOC electronic band structures of achiral $[4AMP]PbBr_4$ using computationally relaxed (**a**) and experimental (**b**) atomic coordinates. Pb-, Br-, and organic-derived electronic states are highlighted in purple, green, and black colors, respectively. Note that the focus of these plots is the degree of SOC near the inorganic-derived conduction band edges, not the alignment of energy levels or the exact fundamental gap. See caption of **Supplementary Figure 8** for further comments regarding observed band structure changes related to experimental and computational geometries.

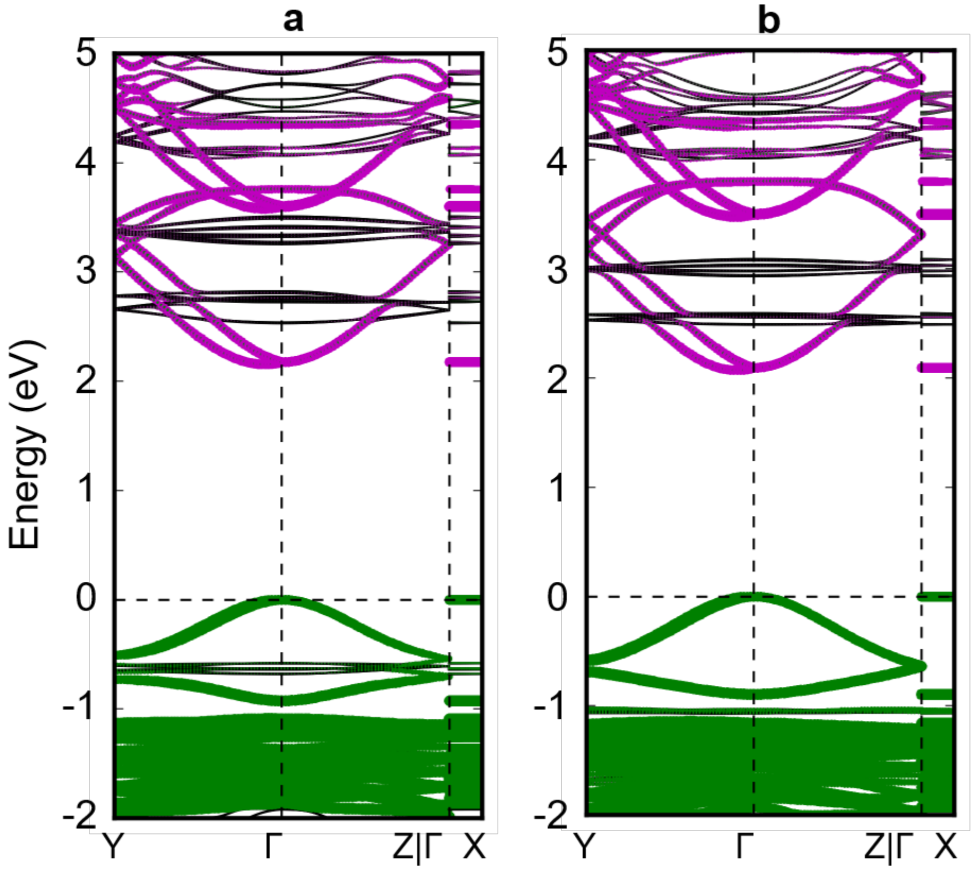

Supplementary Figure 12 | a,b, DFT-PBE+SOC electronic band structures of achiral $[NMA]_2PbBr_4$ using computationally relaxed (**a**) and experimental (**b**) atomic coordinates. Pb-, Br-, and organic-derived electronic states are highlighted in purple, green, and black colors, respectively. Note that the focus of these plots is the degree of SOC near the inorganic-derived conduction band edges, not the alignment of energy levels or the exact fundamental gap. See caption of **Supplementary Figure 8** for further comments regarding observed band structure changes related to experimental and computational geometries.

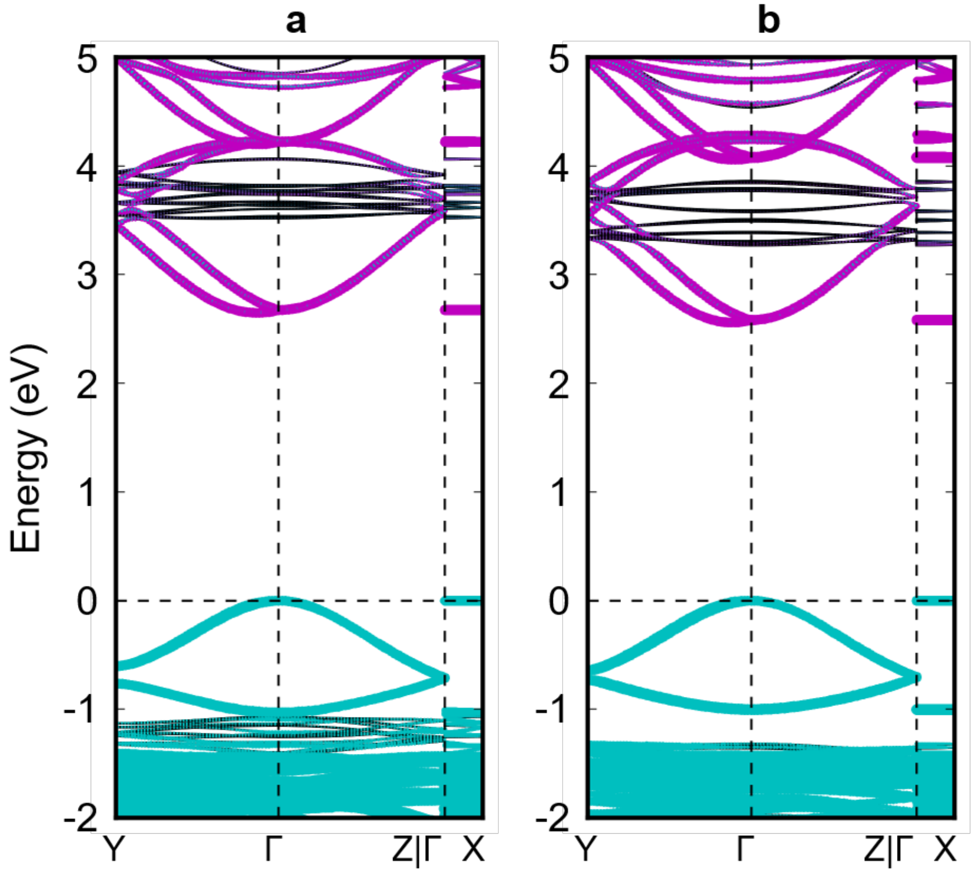

Supplementary Figure 13 | a,b, DFT-PBE+SOC electronic band structures of achiral $[PMA]_2PbCl_4$ using computationally relaxed (**a**) and experimental (**b**) atomic coordinates. Pb-, Cl-, and organic-derived electronic states are highlighted in purple, blue, and black colors, respectively. Note that the focus of these plots is the degree of SOC near the inorganic-derived conduction band edges, not the alignment of energy levels or the exact fundamental gap. See caption of **Supplementary Figure 8** for further comments regarding observed band structure changes related to experimental and computational geometries.

Supplementary Table 2 | Metrics of intra- and inter-octahedral distortions and computed spin-splitting parameters of select chiral and achiral 2D MHPs corresponding to Fig. 5 in the main text.

**Experimental geometries**	σ^2 (°²)	Δd (10^{-5})	$\Delta\beta$ (°)	$\Delta\beta_{in}$ (°)	$\Delta\beta_{out}$ (°)	Max. D_{in} (°)	Max. D_{out} (°)	$\Delta E^{\pm}$ (eV)	k_0 (Å^{-1})	α (eV.Å)
[R-4-Cl-MBA]$_2$PbBr$_4$	49.64	135.3	14.862	12.186	11.857	34.637	15.461	0.044	0.031	0.709
[S-1-1-NEA]$_2$PbBr$_4$	40.17	62	13.922	15.602	2.15	36.044	9.9	0.108	0.049	1.102
[S-4-NO$_2$-MBA]$_2$PbBr$_4$·H$_2$O	34	129.4	12.127	12.097	0.232	31.226	3.833	0.137	0.049	1.398
[S-2-Me-BuA]$_2$PbBr$_4$	7.77	0.191	0.237	0.173	1.084	27.093	1.988	0	0	0
[S-MBA]$_2$PbI$_4$	20.1	56.44	5.857	5.852	0.29	28.55	0.32	0.033	0.02	0.825
[S-4-NH$_3$-MBA]PbI$_4$	14.13	38	3.482	4.544	0.129	28.883	20.292	0.009	0.015	0.3
[R-4-Cl-MBA]$_2$PbI$_4$	12.74	34.74	3	2.952	0.1125	28.195	1.527	0.012	0.014	0.429
[S-MHA]$_2$PbI$_4$	10.77	10.48	0.826	0.91	0.01	26.46	3.148	0	0	0
[NMA]$_2$PbBr$_4$	13.57	15.44	11.67	11.67	0	38.044	0	0.082	0.038	1.079
[PMA]$_2$PbCl$_4$	21.42	4.22	12.26	12.26	0	37.971	0	0.092	0.047	0.979
[4AMP]PbBr$_4$	40.83	104.9	13.77	14.198	1.929	33.497	4.816	0.14	0.049	1.429

**Relaxed geometries**	σ^2 (°²)	Δd (10^{-5})	$\Delta\beta$ (°)	$\Delta\beta_{in}$ (°)	$\Delta\beta_{out}$ (°)	Max. D_{in} (°)	Max. D_{out} (°)	$\Delta E^{\pm}$ (eV)	k_0 (Å^{-1})	α (eV.Å)
[R-4-Cl-MBA]$_2$PbBr$_4$	64	140.5	17.074	13.911	14.066	38.258	17.986	0.062	0.039	0.795
[S-1-1-NEA]$_2$PbBr$_4$	57.03	65.72	18.648	21.056	4.526	42.632	11.899	0.185	0.063	1.468
[S-4-NO$_2$-MBA]$_2$PbBr$_4$·H$_2$O	50.24	77.82	7.624	8.192	1.407	34.897	7.362	0.06	0.035	0.857
[S-2-Me-BuA]$_2$PbBr$_4$	8.36	11.46	0.353	0.338	1.769	30.879	2.833	0	0	0
[S-MBA]$_2$PbI$_4$	20.96	19.37	5.867	5.901	0.04	31.709	1.386	0.03	0.02	0.75
[S-4-NH$_3$-MBA]PbI$_4$	17.59	16.31	0.329	1.309	2.412	30.085	23.446	0.002	0.007	0.143
[R-4-Cl-MBA]$_2$PbI$_4$	15.3	187.88	1.221	1.262	0.8875	34.622	3.059	0	0	0
[S-MHA]$_2$PbI$_4$	12.91	7.1	0.609	0.704	0.07	29.876	4.157	0.003	0.007	0.214
[NMA]$_2$PbBr$_4$	11.61	26.24	12.216	12.216	0	41.51	0	0.075	0.038	0.987
[PMA]$_2$PbCl$_4$	21.32	20.3	14.527	14.527	0	42.68	0	0.11	0.063	0.873
[4AMP]PbBr$_4$	49.81	74.81	15.3	15.64	2.086	37.663	4.792	0.142	0.052	1.365

Supplementary Note 2: First-principles simulations of 2D Cs_2PbBr_4 models

For a broader investigation of the structural origin and evolution of spin-splitting in 2D MHPs, we have systematically constructed multiple series of idealized 2D Cs_2PbBr_4 perovskite models starting from an undistorted (1 × 1) perovskite square lattice (Supplementary Figure 14a). In all the models, there is a single $[PbBr_4]^{2-}$ layer spanning the a-b plane within the unit cell, and rows of Cs^+ cations lie on either side of the $[PbBr_4]^{2-}$ layer along the c-direction. In the undistorted (1 × 1) lattice, Pb-Br bond lengths are fixed to 3 Å (based on the average value found in $CsPbBr_3$), while equatorial Pb-Br-Pb (β) and axial Br-Pb-Br bond angles are 180º. Interoctahedral tilting distortions represent a good starting point for the study, as multiple previous reports emphasize their dominant role in affecting band dispersions and bandgaps for 2D MHPs. Generating a pure interoctahedral tilting distortion while maintaining all Pb-Br bond lengths equal to 3 Å (i.e., $\Delta d = 0$ and $\sigma^2 = 0$) necessitates a ($\sqrt{2} \times \sqrt{2}$)-R45° perovskite lattice (using Wood's notation[28]) with respect to the underlying lattice of squares formed by the Pb-sites (Supplementary Figure 14b), which is indeed frequently observed within 2D MHPs. Two individual sets of ($\sqrt{2} \times \sqrt{2}$)-R45° models have been constructed (Supplementary Figure 15) by tilting the adjacent $PbBr_6$ octahedra symmetrically (i.e., to the same degree) exclusively along in-plane or out-of-plane directions of the perovskite layer, giving rise to β_{in} or β_{out} values that increase from 140º (largest distortion) to 180º (zero distortion). In each case, symmetric tilting engenders a single β_{in} or β_{out} value within the perovskite lattice.

Based on earlier structural characterization of chiral NEA_2PbBr_4,[10] we have created additional sets of ($\sqrt{2} \times \sqrt{2}$)-R45° models by introducing asymmetric tilting for adjacent $PbBr_6$ octahedra exclusively along in-plane or out-of-plane directions (Supplementary Figure 16). In each case, two disparate β angles (140º≤β≤160º) are thus generated within the perovskite lattice, with in-plane or out-of-plane disparity between adjacent bond angles ($\Delta\beta_{in}$ or $\Delta\beta_{out}$) ranging from 5º to 20º in each set of models (Supplementary Figure 16). Whereas the undistorted (1 × 1) and ($\sqrt{2} \times \sqrt{2}$)-R45° models (Supplementary Figure 14) both correspond to the P4/mmm space group (a = b ≠ c), asymmetrically distorted ($\sqrt{2} \times \sqrt{2}$)-R45° Cs_2PbBr_4 models constructed with $\Delta\beta_{in}$ or $\Delta\beta_{out}$, exhibit a reduced orthorhombic symmetry (a ≠ b ≠ c). The conventional space groups and point groups for the asymmetrically distorted models are listed in Supplementary Table 3. $\Delta\beta_{in}$

always creates a local formal dipole about Pb sites leading to a noncentrosymmetric polar Pmc2$_1$ space group. On the other hand, $\Delta\beta_{out}$ can be introduced with and without a resulting local formal dipole about Pb sites leading to noncentrosymmetric polar Pma2 and nonpolar P222$_1$ space groups, respectively (Supplementary Figure 16b,c). See Supplementary Tables 4-6 for the formal dipole analysis for representative models.

Note that $\Delta\beta_{in}$ or $\Delta\beta_{out}$ are always accompanied by non-zero intraoctahedral distortions, Δd and σ^2, whose values are either similar or much smaller compared with those found in experimental MHPs (see Table 1 in the main text). Importantly, the corresponding values of Δd and σ^2 are similar between the sets of models with pure $\Delta\beta_{in}$ and pure $\Delta\beta_{out}$, thereby allowing us to isolate and compare the dominant effects of $\Delta\beta_{in}$ versus $\Delta\beta_{out}$ on the associated electronic band structures.

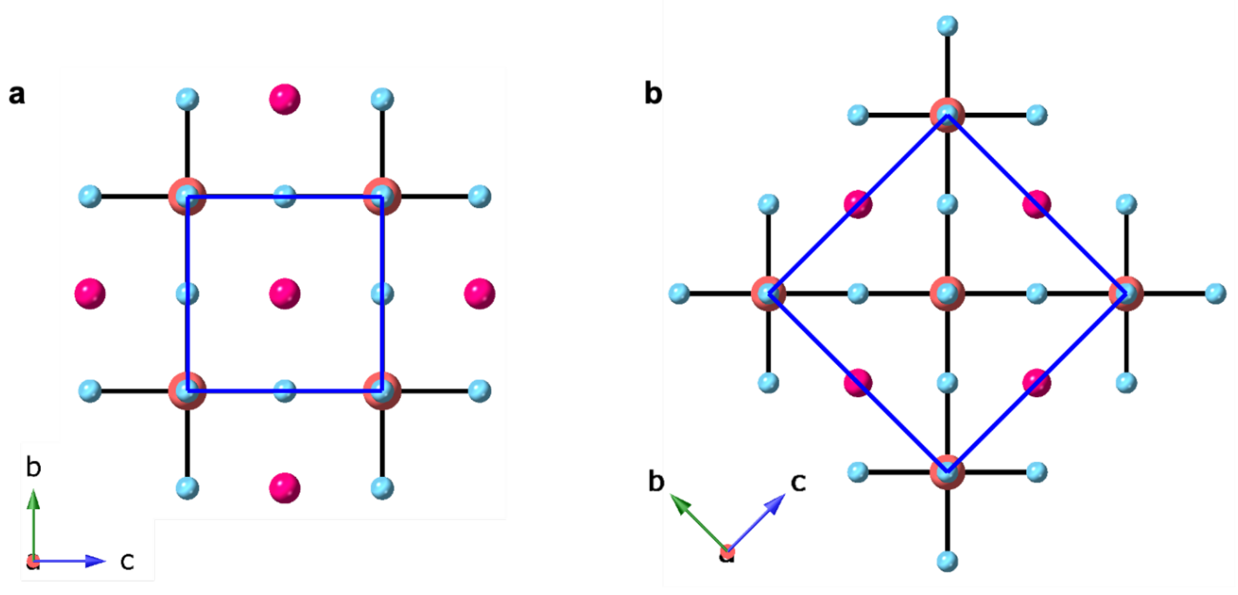

Supplementary Figure 14 | **a**, Undistorted (1×1) perovskite square lattice. **b**, Undistorted $(\sqrt{2} \times \sqrt{2})$-R45° perovskite lattice (using Wood's notation[28,29]). Cs, Pb, and Br atoms are denoted by pink, orange and cyan spheres, respectively.

From the calculated DFT-PBE+SOC band structures, the constructed Cs_2PbBr_4 models involving only one β_{in} or β_{out} value in the $[PbBr_4]^{2-}$ layer lack band spin-splitting (Supplementary Figures 17 and 18). With decreasing β_{in} or β_{out}, the energy width of the doubly degenerate lowest conduction band, which is estimated as the energy difference between the Y and Γ points ($E_Y - E_\Gamma$), decreases significantly and the bandgap increases. This is consistent with earlier theoretical and experimental reports on bandgap correlations with β in 2D MHPs (Supplementary Figures 17 and 18).[3,4,30] Interoctahedral distortions leading to $\beta < 180°$ weaken the antibonding interactions between M- and X-derived orbitals at the band edges, leading to reduced band dispersions and increased bandgaps.[30] In contrast, Cs_2PbBr_4 models with disparate β_{in} or β_{out} values (i.e. $\Delta\beta_{in}$ or $\Delta\beta_{out} \neq 0$) in the $[PbBr_4]^{2-}$ layer exhibit spin-splitting mainly in the conduction bands (CBs) along one of the in-plane directions of the $[PbBr_4]^{2-}$ layer, i.e., $\Gamma - Y$ or $\Gamma - Z$ path in the reciprocal space (Supplementary Figures 19 and 20). The energy width of the singly degenerate lowest conduction subband along the $\Gamma - Y(Z)$ path ($E_{Y(Z)} - E_\Gamma$) is very similar for the corresponding models with the same values of pure $\Delta\beta_{in}$ and pure $\Delta\beta_{out}$ and decreases monotonically with increasing $\Delta\beta_{in}$ or $\Delta\beta_{out}$ (Fig. 6 in main text). Interestingly, the characteristic momentum offset ($\mathbf{k_0}$) increases more prominently with $\Delta\beta_{in}$ rather than $\Delta\beta_{out}$ (Fig. 6 in main text). As the CB spin-splitting magnitude ($\Delta E^{\pm}$) relates to both band dispersion and $\mathbf{k_0}$, $\Delta E^{\pm}$ increases steeply with $\Delta\beta_{in}$, but less significantly with $\Delta\beta_{out}$ (Fig. 6 in main text). This is an intrinsic difference between $\Delta\beta_{in}$ and $\Delta\beta_{out}$ and not, for example, related to the presence of formal local dipole moments (see Supplementary Tables 4-6 and Supplementary Figure 20). The above analysis decoupling the effects of $\Delta\beta_{in}$ and $\Delta\beta_{out}$ is noteworthy, as experimental MHPs exhibiting disparate β angles will not necessarily show strong spin-splitting if the disparity is dominantly due to the β_{out} component, whereas a dominant disparity due to the β_{in} component will yield a sizeable spin-splitting.

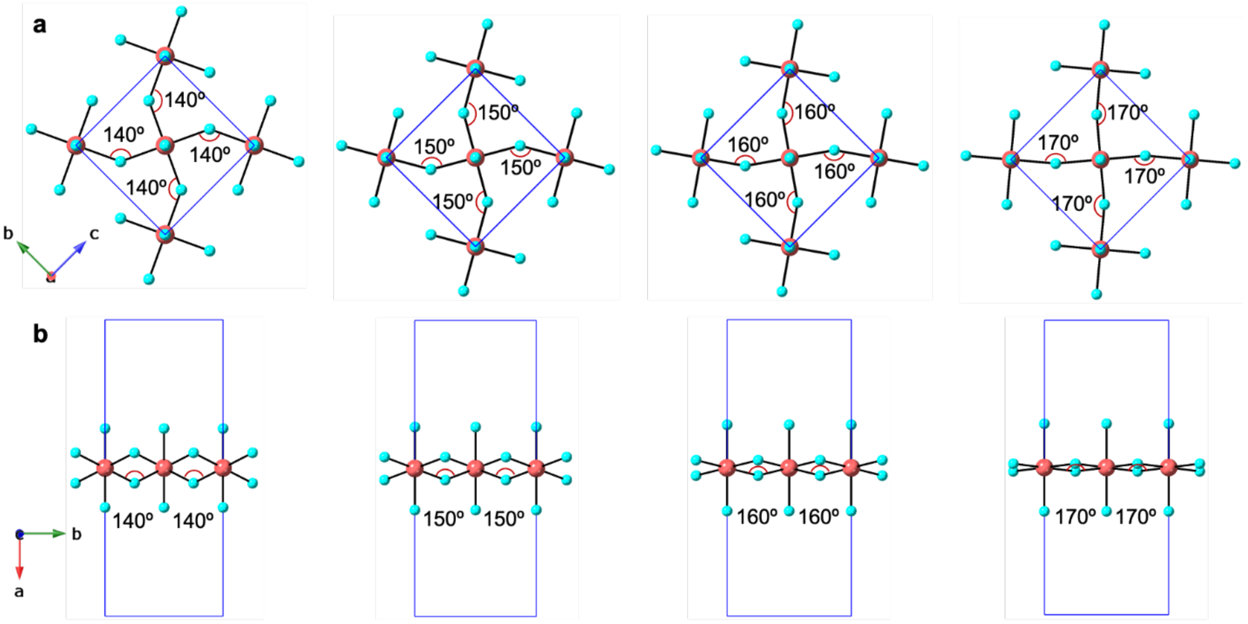

Supplementary Figure 15 | a,b, Series of (√2 × √2)-R45° Cs_2PbBr_4 models wherein the adjacent $PbBr_6$ octahedra are symmetrically tilted purely in-plane (**a**) and purely out-of-plane (**b**), giving rise to a zero disparity in the adjacent Pb-Br-Pb bond angles (i.e. $\Delta\beta_{in}$ or $\Delta\beta_{out} = 0$) across (**a**) and (**b**). Pb and Br atoms are denoted by orange and cyan spheres, respectively. Cs atoms have been omitted in the drawings for visual clarity but are included in computations based on the models.

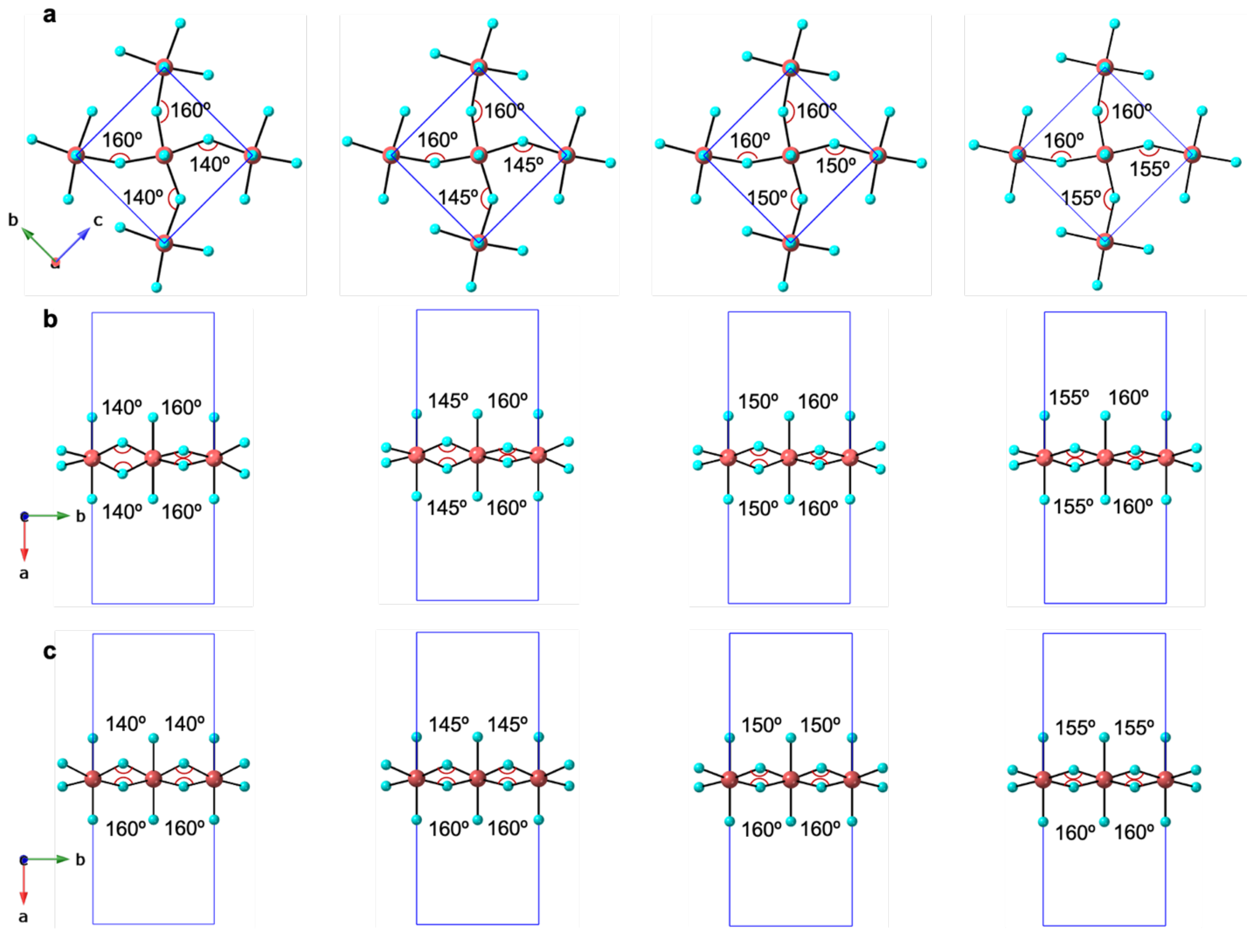

Supplementary Figure 16 | a-c, Series of ($\sqrt{2} \times \sqrt{2}$)-R45° Cs_2PbBr_4 models wherein the adjacent $PbBr_6$ octahedra are asymmetrically tilted purely in-plane (**a**) and purely out-of-plane (**b,c**), giving rise to an increasing Pb-Br-Pb bond angle disparity in the adjacent bond angles (i.e. $\Delta\beta_{in}$ or $\Delta\beta_{out}$) across (**a**) and (**b,c**). Note that the models in (**b**) are different from those in (**c**) in that the former do not exhibit a formal dipole on the Pb site whereas the latter do exhibit a formal dipole on the Pb site. For details, refer to **Supplementary Tables 5 and 6**. Pb and Br atoms are denoted by orange and cyan spheres, respectively. Cs atoms are omitted for visual clarity but are included in computations based on the models.

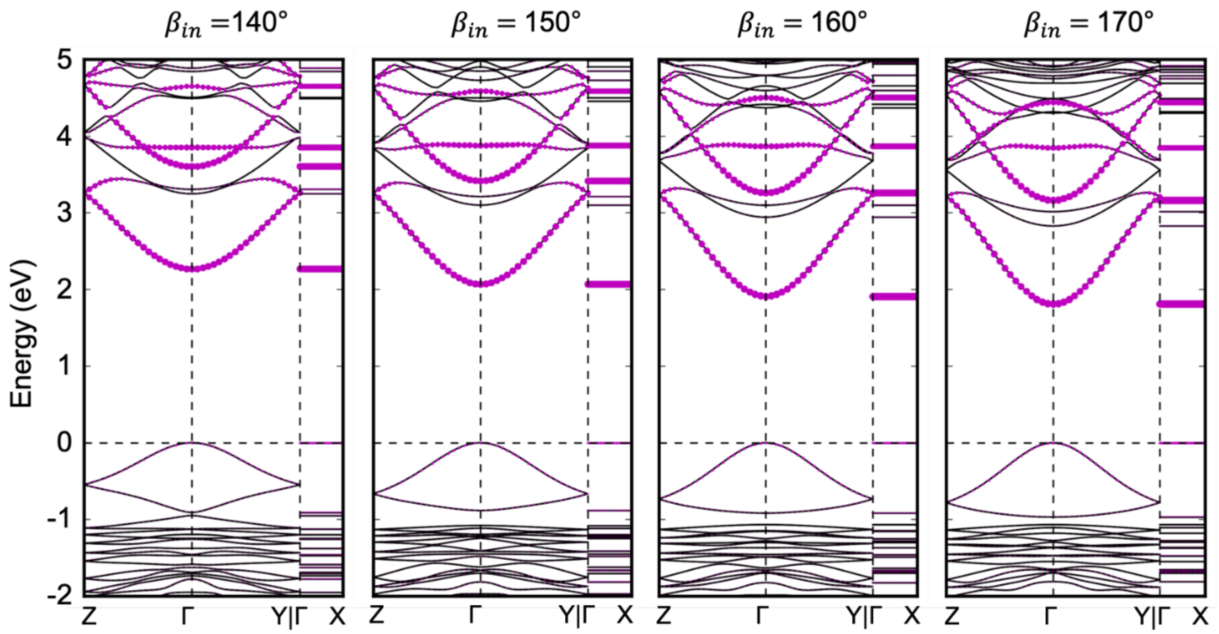

Supplementary Figure 17 | DFT-PBE+SOC band structures for the series of $(\sqrt{2}\times\sqrt{2})-$ R45° Cs_2PbBr_4 models with purely in-plane symmetrical tilting of adjacent $PbBr_6$ octahedra.

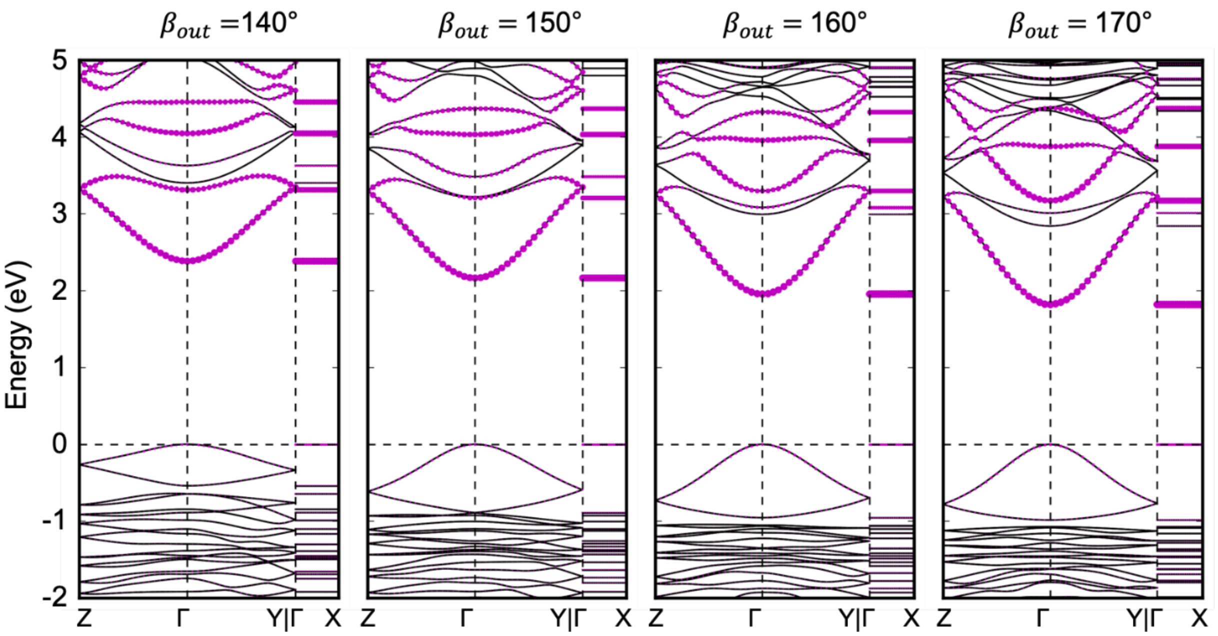

Supplementary Figure 18 | DFT-PBE+SOC band structures for the series of $(\sqrt{2}\times\sqrt{2})-$ R45° Cs_2PbBr_4 models with purely out-of-plane symmetrical tilting of adjacent $PbBr_6$ octahedra.

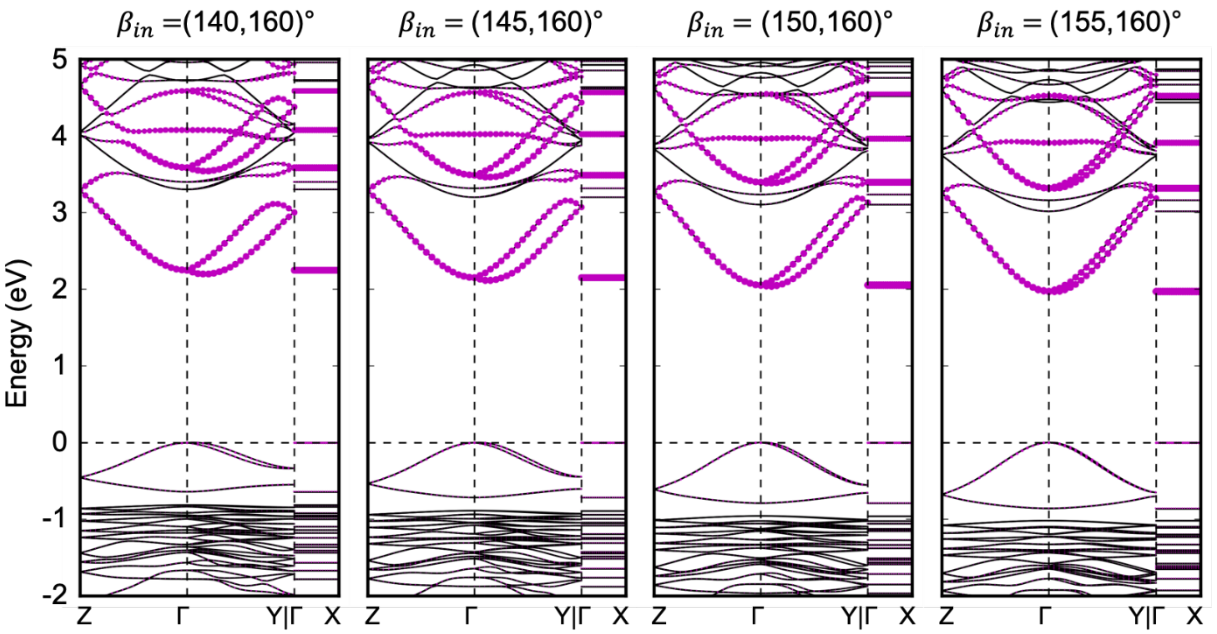

Supplementary Figure 19 | DFT-PBE+SOC band structures for the series of $(\sqrt{2} \times \sqrt{2}) -$ R45° Cs_2PbBr_4 models with purely in-plane asymmetrical tilting of adjacent $PbBr_6$ octahedra. Note that the conduction band splitting increases with $\Delta\beta_{in}$.

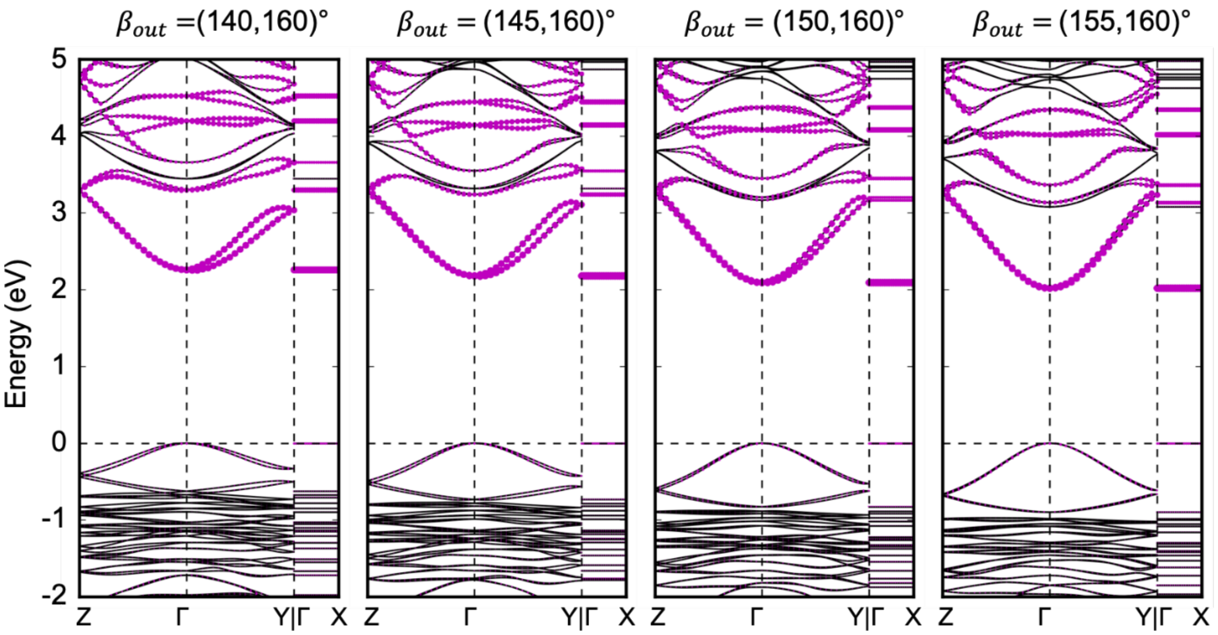

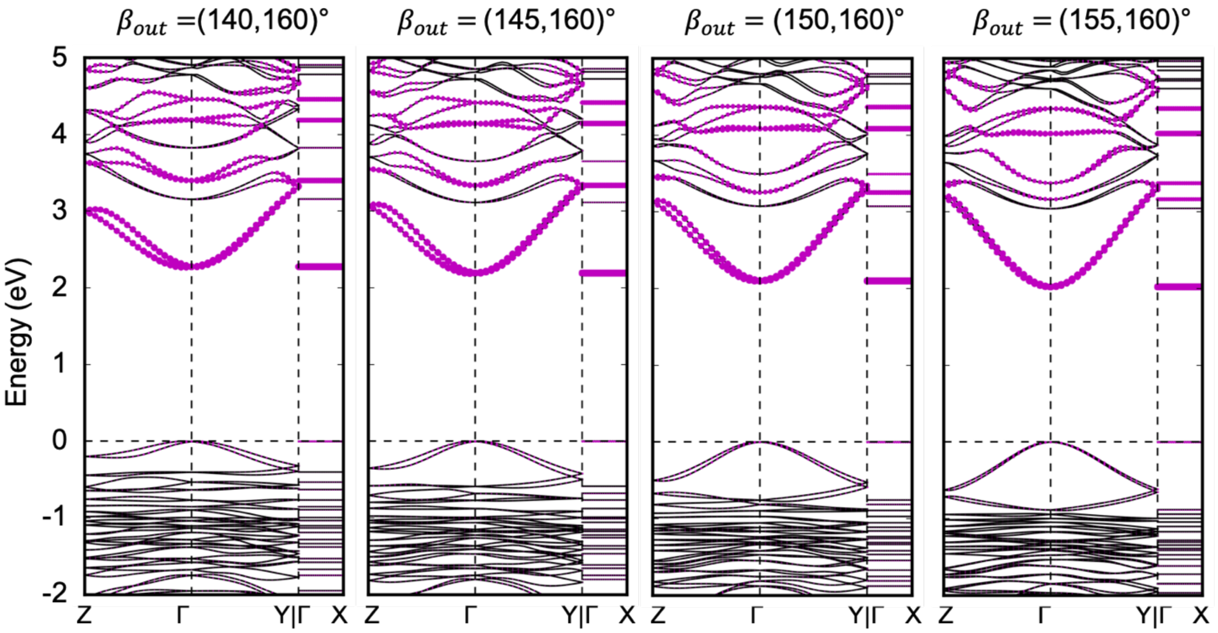

Supplementary Figure 20 | DFT-PBE+SOC band structures for the series of $(\sqrt{2} \times \sqrt{2}) -$ R45° Cs_2PbBr_4 models with purely out-of-plane asymmetrical tilting of adjacent $PbBr_6$ octahedra. Top and bottom rows correspond to models shown in **Supplementary Figure 16b** and **16c**, respectively. Note that the conduction band splitting increases with $\Delta\beta_{out}$, albeit not as significantly as in the case of $\Delta\beta_{in}$.

Supplementary Table 3 | List of space groups and point groups (as determined by PLATON) for the $(\sqrt{2} \times \sqrt{2})$-R45° Cs_2PbBr_4 models constructed with pure $\Delta\beta_{in}$ (top block) and pure $\Delta\beta_{out}$ with (middle block) and without (bottom block) a resulting local formal dipole. For the qualitative analysis of local formal dipoles in representative models, refer to Supplementary Tables 4-6.

$(\sqrt{2} \times \sqrt{2})$-R45° Cs_2PbBr_4 $(\beta_{in}, \beta'_{in})$	Space group	Point group
(140°, 160°)	P$mc2_1$	C_{2V}
(145°, 160°)	P$mc2_1$	C_{2V}
(150°, 160°)	P$mc2_1$	C_{2V}
(155°, 160°)	P$mc2_1$	C_{2V}

$(\sqrt{2} \times \sqrt{2})$-R45° Cs_2PbBr_4 $(\beta_{out}, \beta'_{out})$	Space group	Point group
(140°, 160°)	P$ma2$	C_{2V}
(145°, 160°)	P$ma2$	C_{2V}
(150°, 160°)	P$ma2$	C_{2V}
(155°, 160°)	P$ma2$	C_{2V}

$(\sqrt{2} \times \sqrt{2})$-R45° Cs_2PbBr_4 $(\beta_{out}, \beta'_{out})$	Space group	Point group
(140°, 160°)	$P222_1$	D_2
(145°, 160°)	$P222_1$	D_2
(150°, 160°)	$P222_1$	D_2
(155°, 160°)	$P222_1$	D_2

Supplementary Table 4 | A qualitative analysis of formal local dipole moments in the representative Cs_2PbBr_4 model with $\Delta\beta_{in}$= 20° (see Fig. 6c in the main text). The coordinates of local dipole moment associated with a given Pb site (set as origin) are calculated from the coordinates of the surrounding six Br atoms using the general formula: $\sum q_{Br}\,(\vec{r}_{Br} - \vec{r}_{Pb})$ (here, q_{Br} is taken as -1 e where e is the elementary charge). The unit cell has a C_{2V} point group symmetry with lattice parameters a = 20Å (out-of-plane) and $b = c$ =8.3564Å (in-plane); see Supplementary Note 2 for details. Atomic positions in the table are given as fractional coordinates that must be multiplied by each lattice vector to obtain Cartesian coordinates.

**Atom**	**Fractional coordinates**			**Fractional coordinates with respect to Pb**		
	fa	*fb*	*fc*	*fa-fa(Pb)*	*fb-fb(Pb)*	*fc-fc(Pb)*
Pb	0.5	0.5	0.5	0	0	0
Br	0.5	0.159	0.341	0	-0.341	-0.159
Br	0.5	0.341	0.841	0	-0.159	0.341
Br	0.5	0.7941	0.7059	0	0.2941	0.2059
Br	0.5	0.7059	0.2059	0	0.2059	-0.2941
Br	0.35	0.5	0.5	-0.15	0	0
Br	0.65	0.5	0.5	0.15	0	0
			**local dipole about Pb**	**0**	**0**	**-0.0938** x **8.3564 eÅ**
Pb	0.5	0	0	0	0	0
Br	0.5	0.159	0.341	0	0.159	0.341
Br	0.5	0.341	-0.159	0	0.341	-0.159
Br	0.5	-0.2059	-0.2941	0	-0.2059	-0.2941
Br	0.5	-0.2941	0.2059	0	-0.2941	0.2059
Br	0.35	0	0	-0.15	0	0
Br	0.65	0	0	0.15	0	0
			**local dipole about Pb**	**0**	**0**	**-0.0938** x **8.3564 eÅ**

Supplementary Table 5 | A qualitative analysis of formal local dipole moments in the representative Cs_2PbBr_4 model with $\Delta\beta_{out}$= 20º (see Fig. 6d in the main text, as well as Supplementary Figure 16b). The coordinates of local dipole moment associated with a given Pb site (set as origin) are calculated from the coordinates of the surrounding six Br atoms using the general formula: $\sum q_{Br}\,(\vec{r}_{Br} - \vec{r}_{Pb})$ (here, q_{Br} is taken as -1 e where e is the elementary charge). The unit cell has D_2 point group symmetry with lattice parameters a = 20 Å (out-of-plane) and $b = c$ =8.3564 Å (in-plane); see Supplementary Note 2 for details. Atomic positions in the table are given as fractional coordinates that must be multiplied by each lattice vector to obtain Cartesian coordinates.

**Atom**	**Fractional coordinates**			**Fractional coordinates with respect to Pb**		
	fa	*fb*	*fc*	*fa-fa(Pb)*	*fb-fb(Pb)*	*fc-fc(Pb)*
Pb	0.5	0.5	0.5	0	0	0
Br	0.5538	0.25	0.75	0.0538	-0.25	0.25
Br	0.4462	0.25	0.25	-0.0538	-0.25	-0.25
Br	0.474	0.75	0.25	-0.026	0.25	-0.25
Br	0.526	0.75	0.75	0.026	0.25	0.25
Br	0.3597	0.5	0.6272	-0.1403	0	0.1272
Br	0.6403	0.5	0.3728	0.1403	0	-0.1272
			**local dipole about Pb**	**0**	**0**	**0**
Pb	0.5	0	0	0	0	0
Br	0.474	-0.25	0.25	-0.026	-0.25	0.25
Br	0.526	-0.25	-0.25	0.026	-0.25	-0.25
Br	0.5538	0.25	-0.25	0.0538	0.25	-0.25
Br	0.4462	0.25	0.25	-0.0538	0.25	0.25
Br	0.3597	0	-0.1272	-0.1403	0	-0.1272
Br	0.6403	0	0.1272	0.1403	0	0.1272
			**local dipole about Pb**	**0**	**0**	**0**

Supplementary Table 6 | A qualitative analysis of formal local dipole moments in the representative Cs_2PbBr_4 model exhibiting $\Delta\beta_{out}$= 20° as constructed in Supplementary Figure 16c. The local dipole moment associated with a given Pb site (set as the origin) is calculated from the coordinates of the surrounding six Br atoms using the general formula: $\sum q_{Br}\,(\vec{r}_{Br} - \vec{r}_{Pb})$ (q_{Br} is taken as -1 e, where e is the elementary charge). The unit cell has C_{2v} point group symmetry with lattice parameters a = 20Å (out-of-plane) and $b = c$ = 8.35637Å (in plane); see Supplementary Note 2 for details. Atomic positions in the table are given as fractional coordinates that must be multiplied by each lattice vector in order to obtain Cartesian coordinates.

**Atom**	**Fractional coordinates**			**Fractional coordinates with respect to Pb**		
	fa	*fb*	*fc*	*fa-fa(Pb)*	*fb-fb(Pb)*	*fc-fc(Pb)*
Pb	0.5	0.5	0.5	0	0	0
Br	0.44623	0.25	0.25	-0.05377	-0.25	-0.25
Br	0.52605	0.75	0.75	0.02605	0.25	0.25
Br	0.52605	0.25	0.75	0.02605	-0.25	0.25
Br	0.44623	0.75	0.25000	-0.05377	0.25000	-0.25000
Br	0.64026	0.50	0.37279	0.14026	0	-0.12721
Br	0.35974	0.50	0.62721	-0.14026	0	0.12721
			**local dipole about Pb**	**0.0554x20** eÅ	**0**	**0**
Pb	0.5	0	0	0	0	0
Br	0.44623	0.25	0.25	-0.05377	0.25	0.25
Br	0.52605	-0.25	-0.25	0.02605	-0.25	-0.25
Br	0.52605	0.25	-0.25	0.02605	0.25	-0.25
Br	0.44623	-0.25	0.25	-0.05377	-0.25	0.25
Br	0.64026	0.00000	0.12721	0.14026	0	0.12721
Br	0.35974	0.00000	-0.12721	-0.14026	0	-0.12721
			**local dipole about Pb**	**0.0554x20** eÅ	**0**	**0**

Supplementary Note 3: Analysis of spin-dependent Hamiltonian associated with bulk inversion asymmetry

In this section, we use symmetry theory to analyse the spin-dependent Hamiltonian associated with bulk inversion asymmetry (BIA) in MHPs. The dispersion of the lowest conduction bands and the highest valence bands associated with the metal halide framework can be written as:

$$H(\mathbf{k}) = H_0(\mathbf{k}) + H_{BIA}(\mathbf{k}), \quad (1)$$

where, for example, within an isotropic parabolic band approximation the in-plane dispersion of the conduction and valence bands are given respectively by $H_0(\mathbf{k}) = \pm\frac{\hbar^2\mathbf{k}^2}{2m}$. The angular momentum-dependent Hamiltonian associated with BIA can be written to linear order in wave vector $\mathbf{k}$ and the angular momentum $\mathbf{J}$ in general form as:

$$H_{BIA}(\mathbf{k}) = \sum_{i,j} \alpha_{ij}\, J_i\, k_j, \quad (2)$$

where α_{ij} are phenomenological spin splitting coefficients specific to a given band, J_i are Pauli operators representing the components of the carrier angular momentum, k_j are the components of the carrier wave vector, and the summation is taken over components i, j running over x, y, z. We define coordinates as in the main text (Fig. 3a), i.e. x, y, z are taken respectively along the crystallographic a, b and c directions, where a is the stacking direction and c coincides with a 2-fold rotation or screw axis.

Neumann's principle requires that the BIA Hamiltonian in equation 2 must be invariant under the operations of the crystal point group. We therefore consider the action of rotations and mirror reflections on the terms in equation 2. The 2D MHPs considered in this study have orthorhombic, monoclinic or lower point symmetries (see Table 1 in the main text). We note that wavevector $\mathbf{k}$ (a polar vector) and angular momentum $\mathbf{J}$ (an axial vector) transform in the same manner under rotation. Designating the operator for *n*-fold rotation about an axis u as C_n^u, the polar vector components k_i and axial vector components J_i must transform under 2-fold rotations as,

$$C_2^i\, k_i \rightarrow k_i; \qquad C_2^i\, k_j \rightarrow -k_j\ (j \neq i); \quad (3)$$
$$C_2^i\, J_i \rightarrow J_i; \qquad C_2^i\, J_j \rightarrow -J_j (j \neq i).$$

Mirror reflections act in opposite fashion on the polar and axial vectors. We denote the operator for mirror reflection as M_u, where u is the direction perpendicular to the mirror plane. We have,

$$M_i\, k_i \rightarrow \ -\ k_i; \qquad M_i\, k_j \rightarrow \ +k_j\, (j \neq i); \tag{4}$$

$$M_i\ J_i \rightarrow \ +\ J_i; \qquad M_i\ J_j \rightarrow \ -J_j (j \neq i)\,.$$

Using these relations, we can directly establish which terms are permitted in Hamiltonian H_{BIA} for any point group. These results are tabulated for point groups D_2, C_2, and C_{2v}, which are particularly relevant to this study, in Supplementary Table 7.

Supplementary Table 7 | Angular-momentum-dependent BIA Hamiltonian for point groups relevant to 2D MHPs under study (see Table 1 in the main text). The C_n point groups are designated as C_n^u, where u is the axis of the n-fold rotation. BIA Hamiltonians are given for dispersion in 2D within the plane of the metal halide sheets.

Point group	Principal axis	Notes	BIA Hamiltonian, (2D; **k** in y-z plane)
D_2	-	Inversion asymmetry cannot be described as a vector	$\alpha_{yy}\, J_y\, k_y + \alpha_{zz}\, J_z\, k_z$
C_{2v}^z	Z<br>(C_2 axis)	D_{2h} with inversion asymmetry $\hat{n} = \hat{z}$	$\alpha_{xy}\, J_x\, k_y$
C_2^z	Z (C_2 axis)	C_{2h}^z with inversion asymmetry $\hat{n} = \hat{z}$	$\alpha_{yy}\, J_y\, k_y +$ $\alpha_{zz}\, J_z\, k_z\ +\ \alpha_{xy}\, J_x\, k_y$

Supplementary Note 4: Analysis of spin dependent splitting and spin polarization for [S-4-NO_2-MBA]$_2$PbBr_4·H_2O

In this section, we analyse the spin-splitting and spin polarization for the specific compound, [S-4-NO_2-MBA]$_2$PbBr_4·H_2O (Fig. 4) to illustrate the compatibility of the analysis presented in the main text with global symmetry considerations. This compound has a C_2 point group symmetry and exhibits a dominant out-of-plane spin polarization $\langle\sigma_x\rangle$ component aligned along the stacking a-direction, and relatively much smaller in-plane $\langle\sigma_z\rangle$ and $\langle\sigma_y\rangle$ components for the inorganic-derived frontier conduction band with substantial spin-splitting (see Fig. 4).

The dispersion of the lowest (highest) conduction (valence) bands associated with the metal halide framework can be written according to equation 1 with the angular-momentum-dependent BIA Hamiltonian given by equation 2. Referring to Supplementary Table 7 for the specific case of C_2 point symmetry with the 2-fold axis pointing along the z-direction (denoted as C_2^z), the only symmetry-allowed terms associated with the in-plane wavevector components k_y and k_z are:

$$H_{BIA}(k_y, k_z) = \alpha_{yy} J_y k_y + \alpha_{zz} J_z k_z + \alpha_{xy} J_x k_y \qquad (5)$$

This can be represented in terms of an effective Zeeman Hamiltonian involving the effective k-dependent magnetic field,

$$\mathbf{B_{eff}} = \frac{1}{\mu_b}\{\alpha_{xy} k_y \hat{x} + \alpha_{yy} k_y \hat{y} + \alpha_{zz} k_z \hat{z}\}, \qquad (6)$$

where μ_b is the Bohr magneton. With this expression, spin-splitting can be conceptually understood as resulting from an effective Zeeman splitting under $\mathbf{B_{eff}}$. We can thus understand the qualitative features of the k-dependent spin polarizations $\langle\sigma_x\rangle$, $\langle\sigma_z\rangle$, and $\langle\sigma_y\rangle$ shown in Fig. 4a as follows. Spin-splitting along the $\Gamma - Y$ path (k_y direction) is associated with the terms $\alpha_{xy} k_y\hat{x}$ and $\alpha_{yy} k_y \hat{y}$, which can only result in $\langle\sigma_x\rangle$ and $\langle\sigma_y\rangle$ components, respectively. Spin-splitting along the $\Gamma - Z$ path (k_z direction) results from the term $\alpha_{zz} k_z \hat{z}$, which can only produce $\langle\sigma_z\rangle$ component as shown in Fig. 4a.

We can quantitatively understand the relative magnitudes of the spin polarizations in the out-of-plane versus in-plane directions of the metal halide framework in Fig. 4a. For the BIA Hamiltonian in equation 5, the eigenstates near Γ are found by diagonalizing the matrix,

$$\widetilde{H}_{BIA}(k_y, k_z) = \begin{pmatrix} \alpha_{xy}k_y & \alpha_{yy}k_y - i\alpha_{zz}k_z \\ \alpha_{yy}k_y + i\,\alpha_{zz}k_z & -\alpha_{xy}k_y \end{pmatrix}. \qquad (7)$$

The eigenvalues are,

$$E_{BIA}{}^{\pm} = \pm\sqrt{\alpha_{xy}^2 k_y^2 + \alpha_{yy}^2 k_y^2 + \alpha_{zz}^2 k_z^2} \tag{8}$$

Along the $\Gamma - Y$ and $\Gamma - Z$ paths, this leads to spin-dependent splitting $\Delta E^{\pm} = 2\alpha_{eff}\ k$; along the $\Gamma - Y$ path, $k_z = 0$ so that $\alpha_{eff} = \sqrt{\alpha_{xy}^2 + \alpha_{yy}^2}$, while along the $\Gamma - Z$ path, $k_y = 0$ so that $\alpha_{eff} = \alpha_{zz}$. Linear spin-dependent splitting of both the conduction and valence bands is observed in the DFT calculations (Supplementary Figure 21). A linear fit to the splitting yields the effective splitting parameters α_{eff} given in Supplementary Table 8. Note that the individual terms α_{xy} and α_{yy} cannot be determined based on the splitting, ΔE, alone, but can be determined in conjunction with analysis of the spin polarization (see next section). The effective masses in Supplementary Table 8 were determined by quadratic fit to the average of the two spin-split sub-bands whose energies are given by equation 1 in the main text.

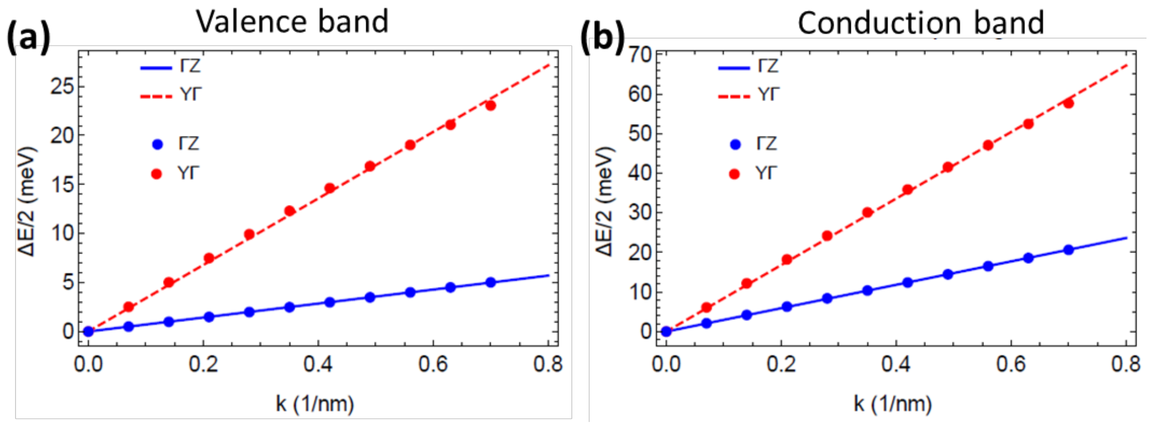

Supplementary Figure 21 | a,b, Spin-splitting calculated using DFT in [S-4-NO_2-MBA]$_2$$PbBr_4$·$H_2O$. Linear-in-k splitting $\Delta E/2 = (E^+ - E^-)/2$ along different paths near the Γ-point calculated in DFT for the inorganic-derived uppermost valence bands, (**a**), and the lowest conduction bands, (**b**). The slopes are listed in Supplementary Table 8.

Diagonalization of the matrix in equation 7 yields Bloch function eigenvectors, $\psi_+,\ \psi_-$, in a basis of the zone-center Bloch functions, $u^{c,v}_{\frac{1}{2},\pm\frac{1}{2}}$, for the given valence (v) or conduction (c) band as,

$$\psi_+^{v,c} = \frac{1}{\sqrt{N}}\left(A u^{c,v}_{\frac{1}{2}\frac{1}{2}} + B\ u^{c,v}_{\frac{1}{2},-\frac{1}{2}}\right); \quad \psi_-^{v,c} = \frac{1}{\sqrt{N}}\left(-B^* u^{c,v}_{\frac{1}{2}\frac{1}{2}} + A\ u^{c,v}_{\frac{1}{2},-\frac{1}{2}}\right). \tag{9}$$

Here, the terms A and B, and the normalization factor, N, are given by the following expressions, with distinct coefficients $\alpha_{i,j}$ for the conduction and valence bands:

$$A = A(k_y, k_z) = \alpha_{xy}k_y + \sqrt{\alpha_{xy}^2 k_y^2 + \alpha_{yy}^2 k_y^2 + \alpha_{zz}^2 k_z^2} \tag{10}$$

$$B = B(k_y, k_z) = (\alpha_{yy}k_y + i\,\alpha_{zz}k_z) \tag{11}$$

$$N = N(k_y, k_z) = |A(k_y, k_z)|^2 + |B(k_y, k_z)|^2 \tag{12}$$

With these expressions, we calculate the spin polarization along a given direction i as a function of the in-plane wavevector components for the valence or conduction bands as follows:

$$\langle\sigma_i\rangle_+^{v,c} = \frac{\langle\psi_+^{v,c}|\sigma_i|\psi_+^{v,c}\rangle}{\langle\psi_+^{v,c}|\psi_+^{v,c}\rangle}; \quad \langle\sigma_i\rangle_-^{v,c} = \frac{\langle\psi_-^{v,c}|\sigma_i|\psi_-^{v,c}\rangle}{\langle\psi_-^{v,c}|\psi_-^{v,c}\rangle}. \tag{13}$$

To analyse the magnitude of the spin polarization, the spin-dependent Bloch functions, $u^{c,v}_{\frac{1}{2},\pm\frac{1}{2}}$, must be specified for the conduction and valence bands.

Supplementary Table 8 | Valence and conduction band parameters near the Γ point for [S-4-NO_2-MBA]$_2PbBr_4 \cdot H_2O$. The effective spin-splitting coefficients α_{eff} were determined by linear fit to the k-dependent splitting $\Delta E = E^+ - E^-$ near Γ calculated using DFT (see Fig. 4 and Supplementary Figure 21) while the effective masses were obtained from a quadratic fit to the average $\bar{E} = (E_1 + E_2)/2$ near Γ. For each band and direction in k-space, the "Rashba energy" $E_R = \frac{{\alpha_{eff}}^2 m}{2\hbar^2}$ and the magnitude of "Rashba wave vector" $k_0 = \frac{m\alpha}{\hbar^2}$ are given. The parameter $\Delta E = 4E_R$ represents the splitting at k_0. Along the $Y - \Gamma$ path, $k_z = 0$ so that $\alpha_{eff} = \sqrt{\alpha_{xy}^2 + \alpha_{yy}^2}$, while along the $\Gamma - Z$ path, $k_y = 0$ so that $\alpha_{eff} = \alpha_{zz}$ (see equation 8).

**Band/Direction**	Effective mass	α_{eff} (eV.Å)	k_0 (1/nm)	E_R (meV)	$\Delta E = 4E_R$ (meV)
Valence band/ $\Gamma - Z$	0.392	α_{zz} =0.07	0.03	0.13	0.5
Valence band/ $Y - \Gamma$	0.497	$\sqrt{\alpha_{xy}^2 + \alpha_{yy}^2}$ =0.34	0.22	3.8	15
Conduction band/ $\Gamma - Z$	0.282	α_{zz} =0.292	0.11	1.6	6.5
Conduction band/ $Y - \Gamma$	0.312	$\sqrt{\alpha_{xy}^2 + \alpha_{yy}^2}$ =0.846	0.34	14.4	57.7

A) Spin polarization in the valence bands

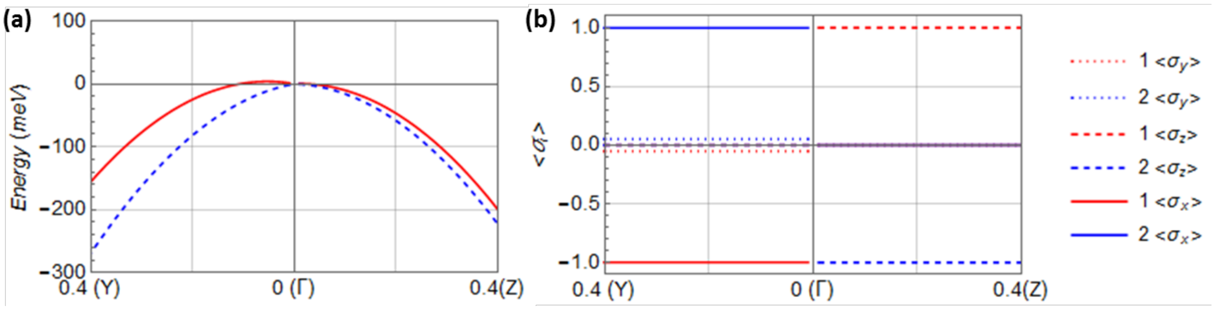

Supplementary Figure 22 | a,b, Spin-splitting and spin polarization for the uppermost valence band in $[S\text{-}4\text{-}NO_2\text{-}MBA]_2PbBr_4\cdot H_2O$ calculated using K.P theory. **a**, Energy versus wavevector along the in-plane $\Gamma - Y$ and $\Gamma - Z$ directions using the average in-plane effective mass from Supplementary Table 8. **b**, Spin polarization components $\langle\sigma_i\rangle$ for $i = x,y,z$. The labels 1 and 2 in the legend denote the upper (red line) and lower (blue line) valence sub-bands, respectively. The values of the individual spin-splitting coefficients were chosen to approximate the spin polarizations calculated in DFT, i.e. $\alpha_{xy} = -0.36$ eV.Å; $\alpha_{zz} = +0.07$ eV.Å; and $\alpha_{yy} = -0.01$ eV.Å, which is consistent with the α_{eff} values given in Supplementary Table 8.

For the MHPs of interest, the valence band Bloch functions near the Γ point can be represented in K.P theory as 2-fold degenerate $J = 1/2$ states with S orbital symmetry with respect to the transformation properties under the operations of the point group:[31,32]

$$u^v_{\frac{1}{2},\frac{1}{2}} = S\ \uparrow,\quad u^v_{\frac{1}{2},-\frac{1}{2}} = S\ \downarrow. \tag{14}$$

In this expression, the symbol S denotes an orbital function that is invariant under the operations of the point group while ↑ (↓) represent spin up (spin down) spin states with the quantization axis taken as the stacking direction $\hat{x}$. Using the Bloch functions in equation 9 and evaluating the spin polarization with equation 13, we find that the x, y, z components of the spin polarization in the valence band are proportional to the relevant components of the effective magnetic field:

$$\langle\sigma_x\rangle^v_{\pm} = \pm\alpha_{xy}k_y\ \frac{2\,A(k_y,k_z)}{N(k_y,k_z)}; \tag{15}$$

$$\langle\sigma_y\rangle^v_{\pm} = \pm\alpha_{yy}k_y\ \frac{2\,A(k_y,k_z)}{N(k_y,k_z)}; \tag{16}$$

$$\langle \sigma_z \rangle_{\pm}^{v} = \pm \alpha_{zz} k_z \frac{2\,A(k_y, k_z)}{N(k_y, k_z)}. \tag{17}$$

With these expressions, we can understand why the dominant spin polarization along the $\Gamma - Y$ path has a magnitude near unity (see Fig. 4a). As described in the main text, for this direction, the dominant spin-splitting term is $\alpha_{xy} k_y$; in the limit that α_{yy} is negligible, $\langle \sigma_x \rangle_{\pm} \rightarrow \pm 1$. Along the $\Gamma - Z$ path, $k_y = 0$ leading to $\langle \sigma_z \rangle_{\pm} \rightarrow \pm 1$ (see Fig. 4a). In Supplementary Figure 22a, we show the spin-dependent valence band dispersion of [S-4-NO_2-MBA]$_2$$PbBr_4$·$H_2O$ calculated in a parabolic band approximation using the parameters in Supplementary Table 8. The corresponding spin polarization components are calculated using equations 15-17 (Supplementary Figure 22b), with specific spin splitting parameters chosen to match the results from DFT calculations in Fig. 4a, compatible with the effective parameters in Supplementary Table 8.

B) Spin polarization in the conduction bands

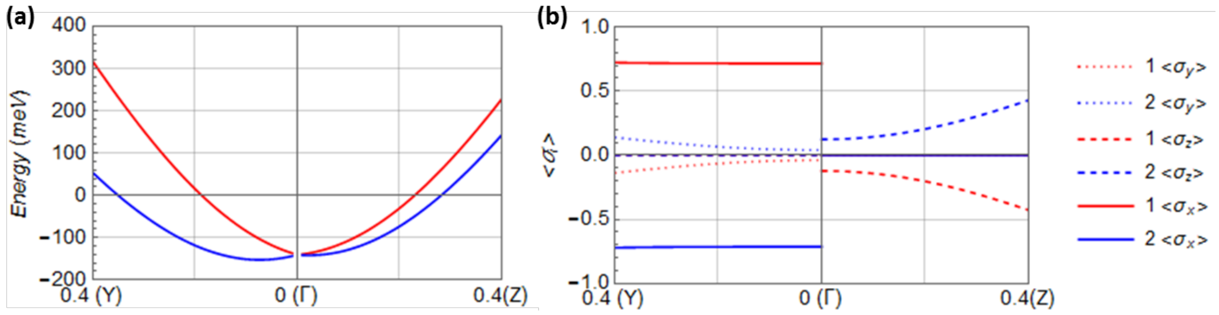

Supplementary Figure 23 | a,b, Spin-splitting and spin-polarization of the inorganic-derived lowest conduction band in [S-4-NO_2-MBA]$_2$$PbBr_4$·$H_2O$ calculated using K.P theory. **a,** Conduction band dispersion (near Γ) along the $\Gamma - Y$ and $\Gamma - Z$. **b,** Spin polarization components $\langle \sigma_i \rangle$ for *i* = *x,y,z*. The labels 1 and 2 in the legend denote the upper (red line) and lower (blue line) conduction sub-bands, respectively. The values of the individual spin-splitting coefficients were chosen to match the DFT-calculated spin polarizations near Γ, i.e., $\alpha_{xy} = -0.86$ eV-Å; $\alpha_{yy} = \alpha_{zz} = 0.29$ eV-Å. The use crystal field parameter $\sin\theta = 0.35$. Effective mass parameters are $m = 1/\gamma_1 = 0.30$; $\gamma_2 = 0.84$.

For the lowest conduction bands associated with the metal halide framework, we can perform calculations similar to those described in the last section for the valence bands. In this case, the

calculation is complicated by the fact that the Bloch functions at the Γ point have an odd parity corresponding to an overall P orbital symmetry. In 3D cubic perovskites, the band edge Bloch functions are represented by the 2-fold degenerate total angular momentum $J = 1/2$ states with P orbital symmetry, corresponding to orbital angular momentum $l = 1$:[32]

$$u^c_{\frac{1}{2},\frac{1}{2}} = \frac{-1}{\sqrt{3}}\{X\uparrow + (Y + i\,Z)\downarrow\}; \quad u^c_{\frac{1}{2},-\frac{1}{2}} = \frac{1}{\sqrt{3}}\{-(Y - i\,Z)\uparrow + X\downarrow\}\,. \tag{18}$$

Here, the quantization axis is taken along the stacking (x) direction as before, and the symbols X, Y, Z denote orbital functions that transform like *x, y, z* under rotations. For 2D MHPs however, the anisotropy between the stacking direction and the in-plane directions results in mixing of the $J = 1/2$ and $J = 3/2$ states.[33,34] Neglecting anisotropy in-plane, the result is that the Bloch functions at the Γ point are modified but can be represented as eigenstates of J_x: [33,34]

$$u^c_{1/2} = -\sin\theta\,X\uparrow - \cos\theta\,\frac{Y + iZ}{\sqrt{2}}\downarrow$$

$$u^c_{-1/2} = -\cos\theta\,\frac{Y - i\,Z}{\sqrt{2}}\uparrow + \sin\theta\,X\downarrow\,. \tag{19}$$

In these expressions, the phase angle θ is given by: [33,34]

$$\tan 2\theta = \frac{2\sqrt{2}\,\Delta_{SO}}{\Delta_{SO} - 3\delta}, \qquad \theta \leq \frac{\pi}{2}. \tag{20}$$

Here, Δ_{SO} is the spin-orbit split-off parameter separating the $J = 1/2$ and $J = 3/2$ conduction band states at Γ while δ is the crystal field reflecting the anisotropy between the stacking direction, x, and the $y - z$ plane. Using these expressions, we find that components of the spin polarization close to the Γ point are proportional to the corresponding components of the effective magnetic field as in the case of valence bands, but with the addition of a "g-factor", $\gamma_i(\theta)$, for each component, i:

$$\langle\sigma_x\rangle_\pm = \pm\alpha_{xy}k_y\,\frac{2\,A(k_y, k_z)}{N(k_y, k_z)}\,\gamma_x(\theta); \tag{21}$$

$$\langle\sigma_y\rangle_\pm = \pm\alpha_{yy}k_y\,\frac{2\,A(k_y, k_z)}{N(k_y, k_z)}\,\gamma_y(\theta); \tag{22}$$

$$\langle\sigma_z\rangle_\pm = \pm\alpha_{zz}k_z\,\frac{2\,A(k_y, k_z)}{N(k_y, k_z)}\,\gamma_z(\theta). \tag{23}$$

In these expressions, the new terms $\gamma_i(\theta)$ are given at the Γ point by,

$$\gamma_x(\theta) = -\cos 2\theta; \qquad \gamma_y(\theta) = \gamma_z(\theta) = -\sin^2\theta \tag{24}$$

The corresponding terms do not appear in equations 15-17 for the valence bands, for which the γ_i-factors are isotropic and equal to +1. The negative sign here is a consequence of the fact that, for the conduction band $P_{1/2}$ states, the spin and the angular momentum are anti-parallel. In fact, for cubic symmetry, all conduction band terms γ_i are equal with the values $\gamma_x = \gamma_y = \gamma_z = -1/3$. In 2D MHPs, the $\gamma_i(\theta)$ factors are highly anisotropic, being dependent on the crystal-field-dependent phase angle θ. For example, with a negative crystal field corresponding to $\sin\theta = 0.35$, $\gamma_y = \gamma_z = -0.12$, while $\gamma_x = -0.76$. The large asymmetry can be understood by examining the conduction band Bloch functions in the limit of large negative crystal field splitting $|\delta| \gg |\Delta_{so}|$. In this limit, $\theta \to 0$, leading to,

$$u^c_{\frac{1}{2}} \to -\frac{(Y + i\,Z)}{\sqrt{2}}\downarrow; \quad u^c_{-\frac{1}{2}} \to -\frac{(Y - i\,Z)}{\sqrt{2}}\uparrow \quad (\delta \gg \Delta_{so}). \tag{25}$$

The corresponding γ-factors in this limit are $\gamma_x = -1$ and $\gamma_y = \gamma_z = 0$.

Away from the Γ point, the γ_i exhibit k-dependence which can be modelled within a 6-band K.P framework using an analogue of the Luttinger Hamiltonian[35] adapted for 2D MHPs and which will be described in detail in a later publication. In Supplementary Figure 23a, we show the spin-splitting of the lowest inorganic-derived conduction band in $[S\text{-}4\text{-}NO_2\text{-}MBA]_2PbBr_4{\cdot}H_2O$ as calculated using a 6-band K.P model using parameters compatible with the frontier band effective masses and the effective spin splitting coefficients determined by fitting to the DFT band structure near the Γ point (Supplementary Table 8). The crystal field is adjusted to yield $\sin\theta = 0.35$ in order to match the DFT-calculated spin polarization near Γ in the *x*-direction. For comparison, the crystal field parameter in other 2D lead bromide MHPs has been measured in the range $\sin\theta = 0.2 - 0.32$.[34,36] In Supplementary Figure 23b**,** the spin polarization components are shown along the $\Gamma - Y$ and $\Gamma - Z$ lines. These plots are in qualitative agreement with the results calculated near Γ using DFT in Fig. 4a. The σ_x spin polarization $\sim \pm 0.7$ in the $\Gamma - Y$ path near Γ is primarily determined by the effect of the crystal field, while the smaller in-plane spin polarization components reflect a combination of the smaller parameters α_{yy} and α_{zz} and smaller effective γ-factors for the in-plane directions.

One feature that is amiss in the model shown in Supplementary Figure 23 is the relative magnitude of the in-plane y and z spin polarization components at the Γ point. Additionally, away

from the Γ point, the K.P model reflected in Supplementary Figures 23 does not match the behaviour of DFT-calculated in-plane spin polarization along the $\Gamma - Y$ path (see Fig. 4a), which exhibits a sign change of the $\langle\sigma_y\rangle_\pm$ polarization away from Γ. These features can be qualitatively captured by extending the model to include anisotropy in the plane of the lead-halide sheets. Analysis of this situation will be published elsewhere.

Supplementary Note 5: Conduction band splitting and spin polarization for $(FC_2H_4NH_3)_2PbCl_4$ with a centrosymmetric P*nma* global space group.

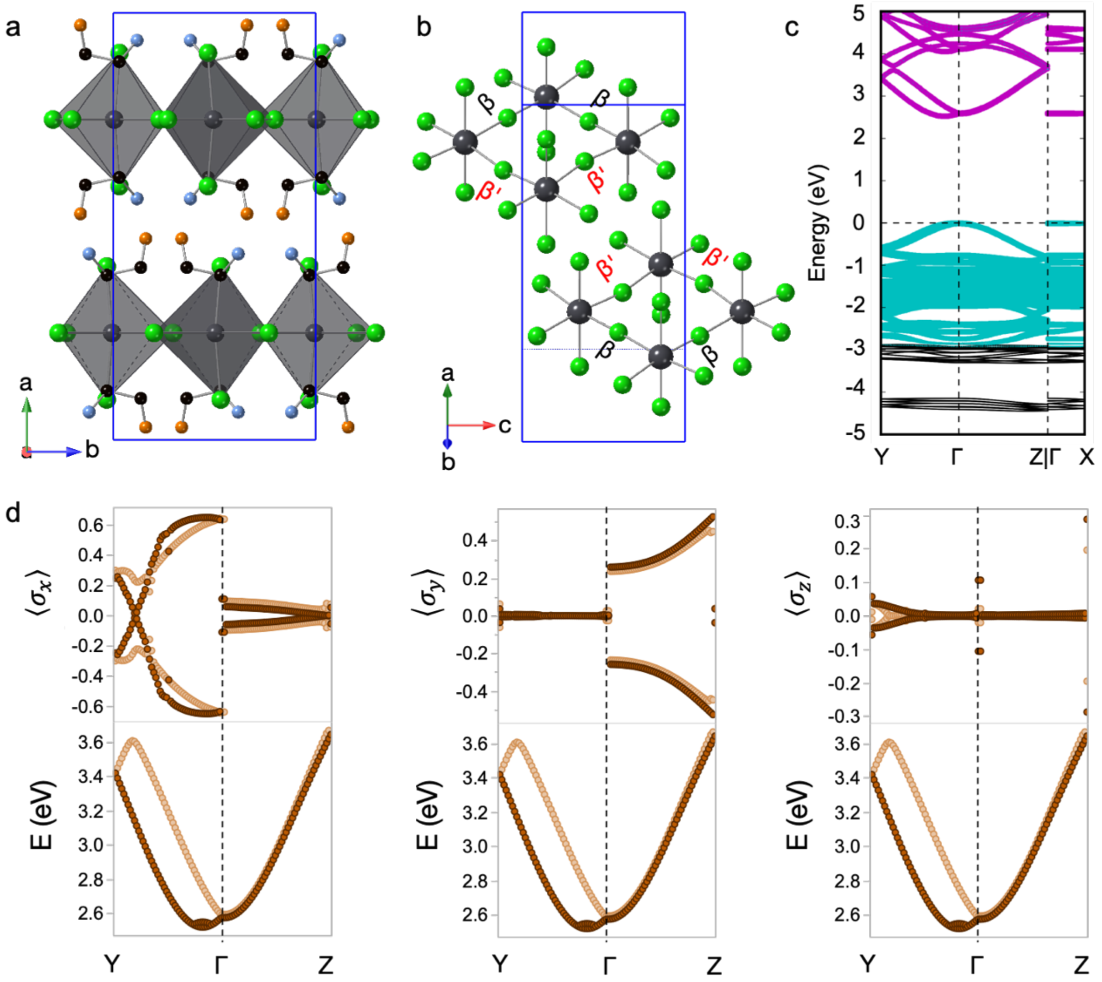

Supplementary Figure 24 | a, Schematic crystal structure of $(FC_2H_4NH_3)_2PbCl_4$ that crystallizes in a centrosymmetric P*nma* global space group. The inversion center is between the two inorganic layers. **b**, View of isolated inorganic framework; the two inorganic layers are nominally noncentrosymmetric (P*mc*2_1 layer group) owing to widely disparate Pb-Cl-Pb bond angles of β = 165.9º and β' = 177.6º (i.e., $\Delta\beta$ = 11.7º). **c**, DFT-PBE+SOC band structure corresponding to relaxed atomic coordinates. **d**, k-dependent spin polarization components $\langle\sigma_x\rangle$, $\langle\sigma_y\rangle$ and $\langle\sigma_z\rangle$ (top panels) for the frontier CBs (bottom panels), shown along the in-plane $\Gamma - Y$ and $\Gamma - Z$ paths. $\langle\sigma_y\rangle$ and $\langle\sigma_z\rangle$ point along the two in-plane directions of the perovskite layer, while $\langle\sigma_x\rangle$ points along the out-of-plane stacking direction. Note that despite a significant CB splitting arising from a local inversion asymmetry of inorganic layers, the net spin polarization of both upper and lower 2-fold degenerate CBs is zero owing to the global inversion symmetry.

Supplementary Note 6: Input geometry files

Supplementary Table 9 | Input geometry.in file of Cs_2PbBr_4 model with a pure in-plane tilting distortion leading to disparate bond angles (β_{in}) of 140º and 160º (i.e., $\Delta\beta_{in} = 20°$).

```
lattice_vector    20.0000000000    0.0000000000    0.0000000000
lattice_vector     0.0000000000    8.3563995361    0.0000000000
lattice_vector     0.0000000000    0.0000000000    8.3563995361

atom    10.000000000   0.000000000    0.000000000 Pb
atom    10.000000000   4.178199768    4.178199768 Pb
atom    10.000000000   1.328731418    2.849468470 Br
atom    10.000011444   6.635663509    5.898935795 Br
atom    10.000000000   2.849468470    7.027667999 Br
atom    10.000000000   5.898935795    1.720736027 Br
atom    13.000000000   0.000000000    0.000000000 Br
atom     7.000000000   0.000000000    0.000000000 Br
atom    13.000000000   4.178199768    4.178199768 Br
atom     7.000000000   4.178199768    4.178199768 Br
atom     7.000000000   0.000000000    4.178199768 Cs
atom     7.000000000   4.178199768    0.000000000 Cs
atom    13.000000000   0.000000000    4.178199768 Cs
atom    13.000000000   4.178199768    0.000000000 Cs
```

Supplementary Table 10 | Input geometry.in file of Cs_2PbBr_4 model with a pure in-plane tilting distortion leading to disparate bond angles (β_{in}) of 145º and 160º (i.e., $\Delta\beta_{in} = 15°$).

lattice_vector	20.0000000000	0.0000000000	0.0000000000
lattice_vector	0.0000000000	8.3563995361	0.0000000000
lattice_vector	0.0000000000	0.0000000000	8.3563995361

atom	10.000000000	0.000000000	0.000000000	Pb
atom	10.000000000	4.178199768	4.178199768	Pb
atom	10.000000000	1.430410743	2.747789145	Br
atom	10.000011444	6.635663509	5.898935795	Br
atom	10.000000000	2.747789145	6.925988674	Br
atom	10.000000000	5.898935795	1.720736027	Br
atom	13.000000000	0.000000000	0.000000000	Br
atom	7.000000000	0.000000000	0.000000000	Br
atom	13.000000000	4.178199768	4.178199768	Br
atom	7.000000000	4.178199768	4.178199768	Br
atom	7.000000000	0.000000000	4.178199768	Cs
atom	7.000000000	4.178199768	0.000000000	Cs
atom	13.000000000	0.000000000	4.178199768	Cs
atom	13.000000000	4.178199768	0.000000000	Cs

Supplementary Table 11 | Input geometry.in file of Cs_2PbBr_4 model with a pure in-plane tilting distortion leading to disparate bond angles (β_{in}) of 150º and 160º (i.e., $\Delta\beta_{in} = 10°$).

lattice_vector	20.0000000000	0.0000000000	0.0000000000	
lattice_vector	0.0000000000	8.3563995361	0.0000000000	
lattice_vector	0.0000000000	0.0000000000	8.3563995361	
atom	10.000000000	0.000000000	0.000000000	Pb
atom	10.000000000	4.178199768	4.178199768	Pb
atom	10.000000000	1.529328465	2.648871422	Br
atom	10.000011444	6.635663509	5.898935795	Br
atom	10.000000000	2.648871422	6.827071190	Br
atom	10.000000000	5.898935795	1.720736027	Br
atom	13.000000000	0.000000000	0.000000000	Br
atom	7.000000000	0.000000000	0.000000000	Br
atom	13.000000000	4.178199768	4.178199768	Br
atom	7.000000000	4.178199768	4.178199768	Br
atom	7.000000000	0.000000000	4.178199768	Cs
atom	7.000000000	4.178199768	0.000000000	Cs
atom	13.000000000	0.000000000	4.178199768	Cs
atom	13.000000000	4.178199768	0.000000000	Cs

Supplementary Table 12 | Input geometry.in file of Cs_2PbBr_4 model with a pure in-plane tilting distortion leading to disparate bond angles (β_{in}) of 155º and 160º (i.e., $\Delta\beta_{in} = 5°$).

```
lattice_vector    20.0000000000     0.0000000000     0.0000000000
lattice_vector     0.0000000000     8.3563995361     0.0000000000
lattice_vector     0.0000000000     0.0000000000     8.3563995361

atom    10.000000000   0.000000000     0.000000000 Pb
atom    10.000000000   4.178199768     4.178199768 Pb
atom    10.000000000   1.625958562     2.552241325 Br
atom    10.000011444   6.635663509     5.898935795 Br
atom    10.000000000   2.552241325     6.730441093 Br
atom    10.000000000   5.898935795     1.720736027 Br
atom    13.000000000   0.000000000     0.000000000 Br
atom     7.000000000   0.000000000     0.000000000 Br
atom    13.000000000   4.178199768     4.178199768 Br
atom     7.000000000   4.178199768     4.178199768 Br
atom     7.000000000   0.000000000     4.178199768 Cs
atom     7.000000000   4.178199768     0.000000000 Cs
atom    13.000000000   0.000000000     4.178199768 Cs
atom    13.000000000   4.178199768     0.000000000 Cs
```

Supplementary Table 13 | Input geometry.in file of Cs_2PbBr_4 model with a pure out-of-plane tilting distortion leading to disparate bond angles (β_{out}) of 140º and 160º (i.e., $\Delta\beta_{out} = 20°$) and no formal dipole on Pb site.

lattice_vector	20.0000000000	0.0000000000	0.0000000000	
lattice_vector	0.0000000000	8.3563699722	0.0000000000	
lattice_vector	0.0000000000	0.0000000000	8.3563699722	
atom	10.000000000	0.000000000	0.000000000	Pb
atom	10.000000000	4.178184986	4.178184986	Pb
atom	8.924650192	2.089092493	2.089092493	Br
atom	10.520957947	6.267277718	6.267277718	Br
atom	11.075349808	2.089092493	6.267277718	Br
atom	9.479042053	6.267278194	2.089092016	Br
atom	12.805204391	0.000000000	1.062981606	Br
atom	7.194795132	0.000000000	7.293388367	Br
atom	12.805204391	4.178184986	3.115199327	Br
atom	7.194795132	4.178184986	5.241170406	Br
atom	7.000000000	0.000000000	4.178184986	Cs
atom	7.000000000	4.178184986	0.000000000	Cs
atom	13.000000000	0.000000000	4.178184986	Cs
atom	13.000000000	4.178184986	0.000000000	Cs

Supplementary Table 14 | Input geometry.in file of Cs_2PbBr_4 model with a pure out-of-plane tilting distortion leading to disparate bond angles (β_{out}) of 145º and 160º (i.e., $\Delta\beta_{out} = 15°$) and no formal dipole on Pb site.

lattice_vector	20.0000000000	0.0000000000	0.0000000000	
lattice_vector	0.0000000000	8.3563699722	0.0000000000	
lattice_vector	0.0000000000	0.0000000000	8.3563699722	
atom	10.000000000	0.000000000	0.000000000	Pb
atom	10.000000000	4.178184986	4.178184986	Pb
atom	9.068450928	2.089092493	2.089092493	Br
atom	10.520958900	6.267277718	6.267277718	Br
atom	10.931550980	2.089092493	6.267277718	Br
atom	9.479043007	6.267666340	2.089092493	Br
atom	12.834528923	0.000000000	0.981287003	Br
atom	7.165473938	0.000000000	7.375082970	Br
atom	12.839260101	4.178184986	3.210602999	Br
atom	7.160741806	4.178184986	5.145766735	Br
atom	7.000000000	0.000000000	4.178184986	Cs
atom	7.000000000	4.178184986	0.000000000	Cs
atom	13.000000000	0.000000000	4.178184986	Cs
atom	13.000000000	4.178184986	0.000000000	Cs

Supplementary Table 15 | Input geometry.in file of Cs_2PbBr_4 model with a pure out-of-plane tilting distortion leading to disparate bond angles (β_{out}) of 150º and 160º (i.e., $\Delta\beta_{out} = 10°$) and no formal dipole on Pb site.

lattice_vector	20.0000000000	0.0000000000	0.0000000000
lattice_vector	0.0000000000	8.3563709259	0.0000000000
lattice_vector	0.0000000000	0.0000000000	8.3563709259

atom	10.000000000	0.000000000	0.000000000	Pb
atom	10.000000000	4.178185463	4.178185463	Pb
atom	9.208440781	2.089092731	2.089092731	Br
atom	10.521054268	6.267278194	6.267278194	Br
atom	10.791751862	2.089092731	6.267278194	Br
atom	9.479139328	6.267278194	2.089092731	Br
atom	12.855998993	0.000000000	0.918272316	Br
atom	7.144000053	0.000000000	7.438098431	Br
atom	12.855998993	4.178185463	3.259913206	Br
atom	7.144000053	4.178185463	5.096457958	Br
atom	7.000000000	0.000000000	4.178185463	Cs
atom	7.000000000	4.178185463	0.000000000	Cs
atom	13.000000000	0.000000000	4.178185463	Cs
atom	13.000000000	4.178185463	0.000000000	Cs

Supplementary Table 16 | Input geometry.in file of Cs_2PbBr_4 model with a pure out-of-plane tilting distortion leading to disparate bond angles (β_{out}) of 155° and 160° (i.e., $\Delta\beta_{out} = 5°$) and no formal dipole on Pb site.

lattice_vector	20.0000000000	0.0000000000	0.0000000000
lattice_vector	0.0000000000	8.3563699722	0.0000000000
lattice_vector	0.0000000000	0.0000000000	8.3563699722

atom	10.000000000	0.000000000	0.000000000	Pb
atom	10.000000000	4.178184986	4.178184986	Pb
atom	9.345004082	2.089092493	2.089092493	Br
atom	10.520958900	6.267277718	6.267277718	Br
atom	10.654997826	2.089092493	6.267277718	Br
atom	9.479043007	6.267277718	2.089092493	Br
atom	12.887493134	0.000000000	0.812237084	Br
atom	7.112507820	0.000000000	7.544132233	Br
atom	12.887493134	4.178184986	3.365947247	Br
atom	7.112507820	4.178184986	4.990421772	Br
atom	7.000000000	0.000000000	4.178184986	Cs
atom	7.000000000	4.178184986	0.000000000	Cs
atom	13.000000000	0.000000000	4.178184986	Cs
atom	13.000000000	4.178184986	0.000000000	Cs

Supplementary Table 17 | Input geometry.in file of Cs_2PbBr_4 model with a pure out-of-plane tilting distortion leading to disparate bond angles (β_{out}) of 140º and 160º (i.e., $\Delta\beta_{out} = 20°$) and formal dipole along the stacking direction on Pb site.

lattice_vector	20.0000000000	0.0000000000	0.0000000000
lattice_vector	0.0000000000	8.3563699722	0.0000000000
lattice_vector	0.0000000000	0.0000000000	8.3563699722

atom	10.000000000	0.000000000	0.000000000	Pb
atom	10.000000000	4.178184986	4.178184986	Pb
atom	8.924650192	2.089092493	2.089092493	Br
atom	10.520957947	6.267277718	6.267277718	Br
atom	10.520957947	2.089092493	6.267277718	Br
atom	8.924650192	6.267278194	2.089092016	Br
atom	12.805204391	0.000000000	1.062981606	Br
atom	7.194795132	0.000000000	7.293388367	Br
atom	12.805204391	4.178184986	3.115199327	Br
atom	7.194795132	4.178184986	5.241170406	Br
atom	7.000000000	0.000000000	4.178184986	Cs
atom	7.000000000	4.178184986	0.000000000	Cs
atom	13.000000000	0.000000000	4.178184986	Cs
atom	13.000000000	4.178184986	0.000000000	Cs

Supplementary Table 18 | Input geometry.in file of Cs_2PbBr_4 model with a pure out-of-plane tilting distortion leading to disparate bond angles (β_{out}) of 145° and 160° (i.e., $\Delta\beta_{out} = 15°$) and formal dipole along the stacking direction on Pb site.

lattice_vector	20.0000000000	0.0000000000	0.0000000000	
lattice_vector	0.0000000000	8.3563699722	0.0000000000	
lattice_vector	0.0000000000	0.0000000000	8.3563699722	
atom	10.000000000	0.000000000	0.000000000	Pb
atom	10.000000000	4.178184986	4.178184986	Pb
atom	9.068450928	2.089092493	2.089092493	Br
atom	10.520958900	6.267277718	6.267277718	Br
atom	10.520956990	2.089092493	6.267277718	Br
atom	9.068449020	6.267666340	2.089092493	Br
atom	12.834528923	0.000000000	0.981287003	Br
atom	7.165473938	0.000000000	7.375082970	Br
atom	12.839260101	4.178184986	3.210602999	Br
atom	7.160741806	4.178184986	5.145766735	Br
atom	7.000000000	0.000000000	4.178184986	Cs
atom	7.000000000	4.178184986	0.000000000	Cs
atom	13.000000000	0.000000000	4.178184986	Cs
atom	13.000000000	4.178184986	0.000000000	Cs

Supplementary Table 19 | Input geometry.in file of Cs_2PbBr_4 model with a pure out-of-plane tilting distortion leading to disparate bond angles (β_{out}) of 150º and 160º (i.e., $\Delta\beta_{out} = 10°$) and formal dipole along the stacking direction on Pb site.

lattice_vector	20.0000000000	0.0000000000	0.0000000000	
lattice_vector	0.0000000000	8.3563709259	0.0000000000	
lattice_vector	0.0000000000	0.0000000000	8.3563709259	
atom	10.000000000	0.000000000	0.000000000	Pb
atom	10.000000000	4.178185463	4.178185463	Pb
atom	9.208440781	2.089092731	2.089092731	Br
atom	10.521054268	6.267278194	6.267278194	Br
atom	10.520860670	2.089092731	6.267278194	Br
atom	9.208248138	6.267278194	2.089092731	Br
atom	12.855998993	0.000000000	0.918272316	Br
atom	7.144000053	0.000000000	7.438098431	Br
atom	12.855998993	4.178185463	3.259913206	Br
atom	7.144000053	4.178185463	5.096457958	Br
atom	7.000000000	0.000000000	4.178185463	Cs
atom	7.000000000	4.178185463	0.000000000	Cs
atom	13.000000000	0.000000000	4.178185463	Cs
atom	13.000000000	4.178185463	0.000000000	Cs

Supplementary Table 20 | Input geometry.in file of Cs_2PbBr_4 model with a pure out-of-plane tilting distortion leading to disparate bond angles (β_{out}) of 155º and 160º (i.e., $\Delta\beta_{out} = 5°$) and formal dipole along the stacking direction on Pb site.

lattice_vector	20.0000000000	0.0000000000	0.0000000000
lattice_vector	0.0000000000	8.3563699722	0.0000000000
lattice_vector	0.0000000000	0.0000000000	8.3563699722

atom	10.000000000	0.000000000	0.000000000	Pb
atom	10.000000000	4.178184986	4.178184986	Pb
atom	9.345004082	2.089092493	2.089092493	Br
atom	10.520958900	6.267277718	6.267277718	Br
atom	10.520956990	2.089092493	6.267277718	Br
atom	9.345002174	6.267277718	2.089092493	Br
atom	12.887493134	0.000000000	0.812237084	Br
atom	7.112507820	0.000000000	7.544132233	Br
atom	12.887493134	4.178184986	3.365947247	Br
atom	7.112507820	4.178184986	4.990421772	Br
atom	7.000000000	0.000000000	4.178184986	Cs
atom	7.000000000	4.178184986	0.000000000	Cs
atom	13.000000000	0.000000000	4.178184986	Cs
atom	13.000000000	4.178184986	0.000000000	Cs

Supplementary Table 21 | Input geometry.in file of [S-MHA]$_2$PbI$_4$ *experimental* structure

```
lattice_vector    33.7103996277    0.0000000000    0.0000000000
lattice_vector     0.0000000000    8.9499998093    0.0000000000
lattice_vector     0.0000000000    0.0000000000    8.9561996460

atom   8.514234543   2.238932133   3.436672688 Pb
atom  25.369434357   2.236067772   5.519526958 Pb
atom   8.340965271   6.711068153   7.914772511 Pb
atom  25.196165085   6.713931561   1.041427016 Pb
atom   8.441083908   4.998127460   5.175877571 I
atom  25.296283722   8.426872253   3.780322313 I
atom   8.414115906   3.951872587   0.697777033 I
atom  25.269315720   0.523127317   8.258421898 I
atom  11.709307671   2.374434948   3.155716896 I
atom  28.564506531   2.100564957   5.800482750 I
atom   5.145892620   6.575564384   7.633816719 I
atom  22.001092911   6.849435329   1.322382927 I
atom   8.385125160   8.443071365   1.746727347 I
atom  25.240325928   4.981928349   7.209472179 I
atom   8.470074654   0.506928265   6.224827290 I
atom  25.325273514   3.968071699   2.731372356 I
atom   5.347481251   2.120881557   3.714225531 I
atom  22.202680588   2.354118347   5.241974354 I
atom  11.507719040   6.829118252   8.192325592 I
atom  28.362918854   6.595881462   0.763874412 I
atom  11.057011604   5.932059765   2.747762203 N
atom  27.912210464   7.492939949   6.208437443 N
atom   5.798188210   3.017939806   7.225862026 N
atom  22.653388977   1.457060575   1.730337739 N
atom   5.747622490   7.598549843   4.075070858 N
atom  22.602821350   5.826449871   4.881128788 N
atom  11.107577324   1.351450205   8.553170204 N
atom  27.962778091   3.123549700   0.403029144 N
atom  10.219914436   6.190455437   2.901907444 H
atom  27.075113297   7.234544277   6.054292202 H
atom   6.635285378   2.759544373   7.380007267 H
atom  23.490486145   1.715455532   1.576192379 H
atom  11.207595825   5.154520035   3.154095650 H
atom  28.062795639   8.270479202   5.802103996 H
atom   5.647603512   3.795479774   7.632195473 H
atom  22.502803802   0.679520130   1.324004173 H
atom  11.182548523   5.837933064   1.871684432 H
atom  28.037748337   7.587066650   7.084515095 H
atom   5.672651768   3.112066984   6.349784374 H
atom  22.527851105   1.362932444   2.606415272 H
```

atom	4.968507767	7.740944386	2.149075985	H
atom	21.823707581	5.684055328	6.807123661	H
atom	11.886692047	1.209055424	6.627175808	H
atom	28.741891861	3.265944481	2.329023838	H
atom	5.299780369	7.632505894	4.842823029	H
atom	22.154979706	5.792493820	4.113376617	H
atom	11.555418968	1.317493796	0.364723027	H
atom	28.410619736	3.157505512	8.591476440	H
atom	6.091401100	8.399924278	3.895892859	H
atom	22.946599960	5.025075436	5.060306549	H
atom	10.763798714	0.550075710	8.373992920	H
atom	27.618999481	3.924924135	0.582206845	H
atom	6.406561375	7.002712727	4.142367363	H
atom	23.261760712	6.422286987	4.813832283	H
atom	10.448638916	1.947287321	8.620467186	H
atom	27.303838730	2.527712584	0.335732251	H
atom	5.955717087	5.615453243	2.362359047	H
atom	22.810916901	7.809546471	6.593840599	H
atom	10.899482727	3.334546566	6.840458870	H
atom	27.754682541	1.140453339	2.115740776	H
atom	4.501989365	5.525568485	1.782525539	H
atom	21.357189178	7.899431229	7.173674107	H
atom	12.353210449	3.424431086	6.260625362	H
atom	29.208410263	1.050569296	2.695574284	H
atom	4.768504620	5.177100182	3.287560940	H
atom	21.623704910	8.247899055	5.668638706	H
atom	12.086695671	3.772899389	7.765660763	H
atom	28.941894531	0.702100515	1.190538883	H
atom	11.746051788	7.800864220	2.878907919	H
atom	28.601251602	5.624135494	6.077291965	H
atom	5.109148502	1.149135470	7.357007504	H
atom	21.964347839	3.325864077	1.599191904	H
atom	11.104609489	7.562266827	5.023442268	H
atom	27.959810257	5.862732887	3.932757139	H
atom	5.750590324	1.387732983	0.545342624	H
atom	22.605789185	3.087266922	8.410857201	H
atom	12.646623611	7.320589542	5.169823170	H
atom	29.501823425	6.104410172	3.786376476	H
atom	4.208575726	1.629410267	0.691723406	H
atom	21.063776016	2.845589638	8.264475822	H
atom	11.662585258	6.100955486	5.127549648	H
atom	28.517784119	7.324044228	3.828649998	H
atom	5.192614555	2.849044323	0.649450362	H
atom	22.047815323	1.625955582	8.306749344	H
atom	13.647183418	5.663309097	3.230662584	H
atom	30.502382278	7.761690617	5.725537300	H

atom	3.208016396	3.286690712	7.708762169	H
atom	20.063217163	1.188309073	1.247437239	H
atom	13.407267570	6.341307640	1.845165253	H
atom	30.262466431	7.083692074	7.111034393	H
atom	3.447932482	2.608692169	6.323265076	H
atom	20.303133011	1.866307855	2.632934570	H
atom	14.769200325	7.461829662	4.025650978	H
atom	31.624401093	5.963170052	4.930548668	H
atom	2.085999250	1.488170028	8.503750801	H
atom	18.941198349	2.986829996	0.452449083	H
atom	14.284849167	8.369503021	2.842644215	H
atom	31.140048981	5.055496693	6.113555431	H
atom	2.570350170	0.580496848	7.320744038	H
atom	19.425550461	3.894503117	1.635455608	H
atom	3.158867121	6.891705513	4.153052330	H
atom	20.014066696	6.533294201	4.803147316	H
atom	13.696332932	2.058294296	8.631152153	H
atom	30.551532745	2.416705608	0.325047612	H
atom	3.434988737	8.412319183	3.868612528	H
atom	20.290187836	5.012680054	5.087586880	H
atom	13.420210838	0.537680149	8.346712112	H
atom	30.275411606	3.937319756	0.609487236	H
atom	2.464028358	6.639888287	1.930204034	H
atom	19.319227219	6.785111427	7.025995731	H
atom	14.391171455	2.310111523	6.408303738	H
atom	31.246372223	2.164888382	2.547895670	H
atom	2.659380198	8.181714058	1.717897892	H
atom	19.514579773	5.243286133	7.238301754	H
atom	14.195819855	0.768285990	6.195997715	H
atom	31.051019669	3.706713915	2.760201931	H
atom	32.032394409	8.549925804	3.058586836	H
atom	15.177196503	4.875073433	5.897612572	H
atom	18.533203125	0.400073767	7.536686897	H
atom	1.678003550	4.074926376	1.419512987	H
atom	31.689493179	7.098083973	2.576725245	H
atom	14.834293365	6.326915741	6.379474163	H
atom	18.876106262	1.851916075	7.054825306	H
atom	2.020906210	2.623083830	1.901374459	H
atom	31.571105957	8.302968979	1.580814242	H
atom	14.715906143	5.122031212	7.375385284	H
atom	18.994493484	0.647031248	6.058914185	H
atom	2.139293909	3.827968597	2.897285461	H
atom	33.584995270	6.877860069	1.435284972	H
atom	16.729795456	6.547139645	7.520914555	H
atom	16.980604172	2.072139740	5.913384914	H
atom	0.125404209	2.402860165	3.042814732	H

atom	0.028282857	8.410153389	1.222727299	H
atom	16.883481979	5.014845848	7.733472347	H
atom	16.826917648	0.539846003	5.700827122	H
atom	33.682117462	3.935153961	3.255372524	H
atom	17.151277542	8.215840340	3.472488642	H
atom	0.296077847	5.209159374	5.483711243	H
atom	33.414321899	0.734159410	7.950588226	H
atom	16.559122086	3.740840435	1.005611300	H
atom	16.626104355	0.095084794	2.234607935	H
atom	33.481304169	4.379915237	6.721591949	H
atom	0.229095951	8.854914665	6.712707520	H
atom	17.084295273	4.570084572	2.243491888	H
atom	0.817072332	7.089948654	3.558647394	H
atom	17.672271729	6.335051060	5.397552490	H
atom	16.038127899	1.860051394	8.036746979	H
atom	32.893325806	2.614948511	0.919452548	H
atom	0.998467624	8.618617058	3.313390970	H
atom	17.853668213	4.806382656	5.642808914	H
atom	15.856732368	0.331382573	7.791490555	H
atom	32.711933136	4.143617630	1.164708972	H
atom	18.760713577	7.292889595	2.280767679	H
atom	1.905512214	6.132110119	6.675431728	H
atom	31.804885864	1.657110333	6.758867741	H
atom	14.949686050	2.817888975	2.197332144	H
atom	18.481962204	8.412731171	1.219718099	H
atom	1.626762509	5.012268066	7.736481667	H
atom	32.083637238	0.537268341	5.697817802	H
atom	15.228437424	3.937731504	3.258381605	H
atom	17.795011520	7.010669231	1.078541160	H
atom	0.939812243	6.414330482	7.877658367	H
atom	32.770587921	1.939330697	5.556641102	H
atom	15.915387154	2.535668612	3.399558783	H
atom	16.177654266	6.252317905	2.559673071	H
atom	33.032855988	7.172681808	6.396526337	H
atom	0.677544773	2.697682142	7.037773132	H
atom	17.532745361	1.777317882	1.918426633	H
atom	15.647558212	7.083163738	1.336390615	H
atom	32.502758026	6.341835976	7.619809151	H
atom	1.207641363	1.866835952	5.814490318	H
atom	18.062841415	2.608164072	3.141709089	H
atom	4.786877632	7.179689884	2.928677559	C
atom	21.642076492	6.245309830	6.027522087	C
atom	12.068322182	1.770309806	7.406777382	C
atom	28.923522949	2.704690695	1.549422383	C
atom	5.026220798	5.745899677	2.561473131	C
atom	21.881420135	7.679100037	6.394726276	C

atom	11.828979492	3.204100370	7.039573193	C
atom	28.684179306	1.270899653	1.916626573	C
atom	11.970561981	6.927299976	3.260056973	C
atom	28.825761795	6.497699738	5.696142673	C
atom	4.884637833	2.022700071	7.738156796	C
atom	21.739837646	2.452299356	1.218042970	C
atom	11.832350731	6.980999470	4.791566372	C
atom	28.687549591	6.444000244	4.164633274	C
atom	5.022849083	1.969000220	0.313466698	C
atom	21.878049850	2.505999804	8.642732620	C
atom	13.430223465	6.506649971	2.803290606	C
atom	30.285423279	6.918349743	6.152908802	C
atom	3.424976349	2.443350077	7.281390667	C
atom	20.280176163	2.031649828	1.674809098	C
atom	14.569635391	7.473249435	3.080932617	C
atom	31.424835205	5.951750278	5.875267029	C
atom	2.285564661	1.476750135	7.559032440	C
atom	19.140764236	2.998250246	1.397167325	C
atom	3.414863586	7.526949883	3.466049433	C
atom	20.270063400	5.898049831	5.490149975	C
atom	13.440336227	1.423049808	7.944149494	C
atom	30.295536041	3.051950693	1.012050390	C
atom	2.427149773	7.500099659	2.373392820	C
atom	19.282348633	5.924900055	6.582807064	C
atom	14.428050041	1.449900031	6.851492405	C
atom	31.283250809	3.025100470	2.104707003	C
atom	32.068702698	7.938650131	2.319655657	C
atom	15.213503838	5.486349583	6.636543751	C
atom	18.496894836	1.011349797	6.797755718	C
atom	1.641695619	3.463650703	2.158444166	C
atom	33.548591614	7.732799530	1.889758348	C
atom	16.693391800	5.692200184	7.066441059	C
atom	17.017007828	1.217200279	6.367858410	C
atom	0.161808640	3.257799625	2.588341475	C
atom	16.919248581	8.171349525	2.534604549	C
atom	0.064048335	5.253650188	6.421595097	C
atom	33.646350861	0.778650105	7.012704372	C
atom	16.791151047	3.696349859	1.943495393	C
atom	1.004569888	7.750699997	2.874939919	C
atom	17.859769821	5.674299717	6.081259727	C
atom	15.850629807	1.199300051	7.353039742	C
atom	32.705829620	3.275699377	1.603159904	C
atom	18.099115372	7.679099560	1.701677918	C
atom	1.243915200	5.745900154	7.254521847	C
atom	32.466484070	1.270900130	6.179777622	C
atom	15.611284256	3.204100370	2.776422024	C

atom	15.850629807	7.124199867	2.283830881	C
atom	32.705829620	6.300799847	6.672369003	C
atom	1.004569888	1.825799942	6.761930466	C
atom	17.859769821	2.649199963	2.194268942	C

Supplementary Table 22 | Input geometry.in file of [S-MHA]$_2$PbI$_4$ *relaxed* structure

lattice_vector	33.09599441	-0.00027373	-0.00073953
lattice_vector	-0.00020541	8.81698237	0.00002157
lattice_vector	-0.00081933	0.00000876	8.91387711

atom	8.38690736	2.18398062	4.27541346	Pb
atom	24.93433044	2.22429691	4.63684732	Pb
atom	8.16159133	6.62434074	8.73216599	Pb
atom	24.70941088	6.60089164	0.17970555	Pb
atom	8.22870827	5.01859157	5.92053680	I
atom	24.77618084	8.20692613	2.99155865	I
atom	8.31941711	3.79096289	1.46464203	I
atom	24.86639119	0.61740099	7.44763398	I
atom	11.64132920	2.31796646	4.00524833	I
atom	28.18867701	2.09114338	4.90764082	I
atom	4.90699907	6.49239230	8.46291638	I
atom	21.45477933	6.73275570	0.44863855	I
atom	8.19277590	8.25197835	2.64416882	I
atom	24.73977543	4.97343088	6.26765185	I
atom	8.35473313	0.55678526	7.10144676	I
atom	24.90241694	3.85183129	1.81066391	I
atom	5.17930559	2.10667125	4.56855045	I
atom	21.72680113	2.30224211	4.34312775	I
atom	11.36944231	6.70233535	9.02608659	I
atom	27.91711648	6.52323718	-0.11379782	I
atom	10.82160303	5.80189616	3.49682650	N
atom	27.36891726	7.42401324	5.41533928	N
atom	5.72751416	3.00878403	7.95291490	N
atom	22.27580036	1.39919755	0.95867900	N
atom	5.44550719	7.48454628	5.03772433	N
atom	21.99237076	5.74125229	3.87448155	N
atom	11.10094443	1.32429668	9.49448328	N
atom	27.64940477	3.08417921	-0.58094546	N
atom	9.87743739	5.95719558	3.89160120	H
atom	26.42488711	7.26909089	5.02008152	H
atom	6.67143802	2.85285466	8.34798140	H
atom	23.21964465	1.55510721	0.56338511	H
atom	11.13315028	4.84972121	3.79681818	H
atom	27.68075788	8.37627146	5.11591979	H
atom	5.41640987	3.96122013	8.25255911	H
atom	21.96465595	0.44674324	0.65906487	H
atom	10.74485969	5.79213556	2.45612706	H
atom	27.29172567	7.43334824	6.45603080	H
atom	5.80458801	3.01825253	6.91222093	H
atom	22.35310159	1.38977365	1.99931935	H

atom	4.94128549	7.79325662	3.05017180	H
atom	21.48923049	5.43384734	5.86254686	H
atom	11.60527899	1.01543465	7.50698940	H
atom	28.15257066	3.39207083	1.40708262	H
atom	5.13637161	7.05376531	5.93902816	H
atom	21.68313720	6.17192035	2.97316551	H
atom	11.41040217	1.75596643	1.48165457	H
atom	27.95756713	2.65268093	7.43193891	H
atom	5.45292468	8.52686567	5.15395512	H
atom	21.99952100	4.69889964	3.75850240	H
atom	11.09483463	0.28204606	9.61119919	H
atom	27.64338759	4.12647966	-0.69753052	H
atom	6.42332505	7.18609873	4.88083879	H
atom	22.97030826	6.03946840	4.03105201	H
atom	10.12275017	1.62128091	9.33728940	H
atom	26.67111743	2.78720725	-0.42429033	H
atom	5.87947618	5.50629727	3.20698517	H
atom	22.42691424	7.72094747	5.70309371	H
atom	10.66501662	3.30168564	7.66303906	H
atom	27.21269733	1.10578966	1.24865354	H
atom	4.22668672	5.41094536	2.60101882	H
atom	20.77468845	7.81624893	6.31056306	H
atom	12.31778989	3.39838802	7.05717635	H
atom	28.86479361	1.00908337	1.85616181	H
atom	4.56687701	5.00365931	4.29622727	H
atom	21.11321069	8.22245760	4.61474753	H
atom	11.97707066	3.80575123	8.75223589	H
atom	28.52592929	0.60253399	0.16049783	H
atom	11.47879159	7.76268594	3.30002607	H
atom	28.02546068	5.46293075	5.61150748	H
atom	5.06856803	1.04858781	7.75590959	H
atom	21.61690894	3.35942168	1.15547994	H
atom	10.63172905	7.55941712	5.61208160	H
atom	27.17996964	5.66728892	3.29909035	H
atom	5.91618804	1.25068959	1.15429745	H
atom	22.46236120	3.15675844	7.75697604	H
atom	12.33846545	8.00371039	5.63979127	H
atom	28.88678086	5.22338598	3.27192019	H
atom	4.20894820	0.80842526	1.18171174	H
atom	20.75505889	3.59928461	7.72987235	H
atom	11.86081540	6.33011563	5.99684790	H
atom	28.40912924	6.89703419	2.91572728	H
atom	4.68835299	2.48142277	1.53889518	H
atom	21.23415997	1.92602662	7.37285434	H
atom	13.55632080	5.69004214	4.17804613	H
atom	30.10434228	7.53492461	4.73528502	H

atom	2.99291204	3.12299266	8.63406692	H
atom	19.54073376	1.28538594	0.27778935	H
atom	13.10425185	5.95133062	2.48973705	H
atom	29.65103003	7.27402857	6.42332877	H
atom	3.44489827	2.86196032	6.94568156	H
atom	19.99352514	1.54549074	1.96608852	H
atom	14.42907908	8.02759673	4.31101345	H
atom	30.97504253	5.19611323	4.60325042	H
atom	2.12003544	0.78444766	8.76580942	H
atom	18.66826183	3.62405036	0.14766563	H
atom	13.73413266	8.40363100	2.74074647	H
atom	30.28033768	4.82199210	6.17407949	H
atom	2.81223797	0.41120489	7.19364971	H
atom	19.36127319	3.99641832	1.71966350	H
atom	2.68195536	6.62661391	4.75231787	H
atom	19.22932104	6.59708101	4.15838444	H
atom	13.86342068	2.18529636	9.20831031	H
atom	30.41066361	2.22610093	-0.29767569	H
atom	3.05255364	8.35786664	4.78392433	H
atom	19.60000165	4.86569759	4.13247412	H
atom	13.49496306	0.45365989	9.24113362	H
atom	30.04258382	3.95805272	-0.32229963	H
atom	2.49153673	6.87312581	2.15948985	H
atom	19.03593009	6.36098679	6.75050612	H
atom	14.05132769	1.93581701	6.61442363	H
atom	30.60410759	2.46086831	2.29509953	H
atom	2.64271318	8.60634927	2.40790281	H
atom	19.19272701	4.62709062	6.51127634	H
atom	13.90743198	0.20254804	6.86676802	H
atom	30.45330958	4.19526967	2.05575104	H
atom	31.25827274	8.77141165	2.81824710	H
atom	14.71451396	4.44208046	6.10011544	H
atom	18.38934410	0.05822483	7.27374907	H
atom	1.83686116	4.36195051	1.64425935	H
atom	31.22164694	7.04562220	2.40608387	H
atom	14.66963434	6.17014019	6.50174177	H
atom	18.41761443	1.78464991	6.86385860	H
atom	1.87442635	2.63251160	2.04032606	H
atom	31.02779795	8.25773113	1.13446179	H
atom	14.48100893	4.96499094	7.78070270	H
atom	18.61720948	0.57524837	5.59061835	H
atom	2.07012419	3.83265446	3.32291245	H
atom	33.35598803	7.30818404	1.08759415	H
atom	16.80466843	5.92589307	7.82236787	H
atom	16.28449594	1.51280718	5.54452857	H
atom	-0.25774047	2.88238356	3.36475118	H

atom	0.29350428	9.02696070	1.45402121	H
atom	16.84624725	4.20531773	7.46780731	H
atom	16.25907705	-0.20596754	5.90966283	H
atom	32.80363276	4.60381911	3.01411507	H
atom	16.87563019	8.53899711	3.40291962	H
atom	0.32489067	4.68307827	5.51323608	H
atom	32.76881229	0.27469250	7.85956460	H
atom	16.22160381	4.13412013	1.05534212	H
atom	16.08766260	0.19580497	1.91047998	H
atom	32.63301352	4.21308359	7.00639439	H
atom	0.45789947	8.62227258	6.36510996	H
atom	17.00811970	4.60243327	2.55036967	H
atom	0.43789309	6.82145533	3.58355231	H
atom	16.98264900	6.39827102	5.32490042	H
atom	16.10464491	1.99862361	8.03948684	H
atom	32.65553328	2.41665224	0.86594397	H
atom	0.57733731	8.55369321	3.87654955	H
atom	17.12791690	4.66492680	5.04185984	H
atom	15.97359802	0.26584761	8.33270312	H
atom	32.51752265	4.15124267	0.58689818	H
atom	18.17802400	6.74575091	2.10654236	H
atom	1.62951942	6.47805285	6.80480548	H
atom	31.46353436	2.07102990	6.57089772	H
atom	14.91686468	2.33705654	2.34396023	H
atom	18.51509659	8.37952108	1.52812963	H
atom	1.96552781	4.84536951	7.38671572	H
atom	31.12568044	0.43889366	5.98857658	H
atom	14.57963841	3.96896042	2.92727368	H
atom	17.42738134	7.31997981	0.61826127	H
atom	0.87921737	5.90778386	8.29488132	H
atom	32.21123871	1.50128590	5.07941190	H
atom	15.66576215	2.90611568	3.83515475	H
atom	15.87666067	6.26198625	3.22919097	H
atom	32.42425630	6.96155679	5.68241111	H
atom	0.67152418	2.55289157	7.69010939	H
atom	17.21947395	1.85587235	1.22298149	H
atom	15.17979730	6.65910995	1.66574095	H
atom	31.72716626	6.56748006	7.24658381	H
atom	1.36575079	2.15892942	6.12466557	H
atom	17.91496688	2.24819497	2.78833142	H
atom	4.58056900	7.12996334	3.85085203	C
atom	21.12793268	6.09645958	5.06155538	C
atom	11.96540084	1.67933546	8.30742575	C
atom	28.51328255	2.72871478	0.60644242	C
atom	4.82953095	5.68231804	3.47440614	C
atom	21.37677887	7.54443218	5.43678083	C

atom	11.71508118	3.12665441	7.93057863	C
atom	28.26300610	1.28114239	0.98226367	C
atom	11.78681385	6.89049991	3.89551615	C
atom	28.33402341	6.33539431	5.01668225	C
atom	4.76137427	1.92108472	8.35140210	C
atom	21.30949850	2.48684376	0.56024447	C
atom	11.64070048	7.20314959	5.36923340	C
atom	28.18872960	6.02362373	3.54269884	C
atom	4.90769726	1.60820279	0.91125788	C
atom	21.45382334	2.79941841	8.00026812	C
atom	13.18278868	6.44726792	3.47035548	C
atom	29.72992016	6.77797619	5.44279046	C
atom	3.36587496	2.36572096	7.92613124	C
atom	19.91413333	2.04221966	0.98594002	C
atom	14.18764506	7.58885319	3.32963458	C
atom	30.73415452	5.63594966	5.58428557	C
atom	2.36012543	1.22505917	7.78492625	C
atom	18.90867801	3.18296465	1.12822615	C
atom	3.11354845	7.45113978	4.16074318	C
atom	19.66091562	5.77450074	4.75265588	C
atom	13.43276643	1.35986977	8.61732348	C
atom	29.98059107	3.04895511	0.29733256	C
atom	2.29316702	7.67672766	2.88644118	C
atom	18.84051539	5.55303030	6.02763469	C
atom	14.25336200	1.13466797	7.34304600	C
atom	30.80203525	3.26802516	1.57204981	C
atom	31.56833767	8.03211586	2.06419383	C
atom	15.02081481	5.18738964	6.84977372	C
atom	18.07562754	0.79697703	6.52066300	C
atom	1.52814332	3.61569067	2.39198248	C
atom	33.08220684	8.04800222	1.85840945	C
atom	16.53462768	5.17990919	7.05621938	C
atom	16.56183590	0.77402912	6.31506769	C
atom	0.01461585	3.62925700	2.60028019	C
atom	16.53306413	8.15054713	2.42971352	C
atom	-0.01681609	5.07400836	6.48576339	C
atom	33.10938888	0.66579088	6.88673501	C
atom	16.56275029	3.74219308	2.02763165	C
atom	0.78722948	7.76627036	3.13284308	C
atom	17.33501413	5.45725309	5.78111020	C
atom	15.75972848	1.05211926	7.58893756	C
atom	32.30773523	3.35818133	1.32460984	C
atom	17.72485509	7.62723057	1.63202341	C
atom	1.17584187	5.59806919	7.28163361	C
atom	31.91597071	1.19105081	6.09288064	C
atom	15.36974428	3.21675139	2.82202771	C

atom	15.45705432	7.09294242	2.64280353 C
atom	32.00403983	6.13186861	6.27019940 C
atom	1.08986709	1.72302957	7.10119865 C
atom	17.63864854	2.68497399	1.81232090 C

Supplementary Table 23 | Input geometry.in file of $[R\text{-}4\text{-}Cl\text{-}MBA]_2PbI_4$ *experimental* structure

lattice_vector	16.1831008392	2.2271317766	1.0047295651
lattice_vector	0.0000000000	8.9131002426	0.0000000000
lattice_vector	0.0000000000	0.0505045958	9.0710597756

atom	6.345555782	9.197631836	0.899313211	Pb
atom	6.389574051	4.735558033	5.433313370	Pb
atom	6.311409473	1.904080033	7.078939438	I
atom	6.385690212	3.069958448	2.710120678	I
atom	6.303479671	6.553143024	8.265122414	I
atom	6.355427265	7.474630833	3.663787365	I
atom	9.553046227	8.864001274	0.864598691	I
atom	3.226586580	4.591647148	5.159380913	I
atom	3.190660477	9.175396919	1.148285985	I
atom	9.566639900	5.015275478	5.227080822	I
atom	15.759104729	8.247529030	7.384387970	Cl
atom	13.185991287	3.881022930	5.034882545	Cl
atom	15.679807663	3.644351006	1.253778219	Cl
atom	13.132586479	8.368748665	2.665834188	Cl
atom	9.861982346	5.000369072	9.483778954	C
atom	2.702577829	4.448076248	1.321628690	C
atom	11.468964577	9.232036591	6.473082066	C
atom	1.280083537	4.950664997	1.475510240	C
atom	11.315225601	4.707625866	9.761775017	C
atom	1.252572536	0.925132632	5.288183212	C
atom	0.823719561	6.074328423	0.831251919	C
atom	2.673448324	0.707648695	5.793667316	C
atom	10.049706459	9.608740807	6.029381752	C
atom	11.710092545	3.486485958	1.298499107	C
atom	15.683043480	8.691570282	1.926144719	C
atom	0.707201242	0.148054793	4.352660656	C
atom	12.035372734	8.078289032	6.044716358	C
atom	14.072825432	8.623060226	7.070154190	C
atom	14.847995758	3.559174538	5.503632069	C
atom	15.548725128	2.589728594	4.929397106	C
atom	13.545256615	9.801801682	7.508187771	C
atom	13.639118195	5.306402683	9.582217216	C
atom	0.524332702	1.914878845	5.864337921	C
atom	12.253844261	10.079956055	7.183092117	C
atom	15.233154297	6.815598011	3.295156479	C
atom	15.393366814	4.357771397	6.461832047	C
atom	12.289447784	5.586107731	9.371428490	C
atom	13.050053596	3.164191246	1.463330150	C
atom	0.404577166	4.218871593	2.245713711	C

atom	14.828577042	7.905230522	2.625993013	C
atom	13.983818054	4.051214695	1.043258190	C
atom	13.336494446	7.766558170	6.343202591	C
atom	3.029476881	4.182368755	8.932587624	C
atom	9.907295227	9.879553795	4.551935673	C
atom	2.959889412	8.231128693	6.243233204	C
atom	9.090047836	3.790691137	8.955087662	C
atom	9.822964668	5.702673912	8.801062584	H
atom	2.816490889	3.636889935	1.859575748	H
atom	1.415406585	6.581123829	0.319922447	H
atom	2.826751232	1.312528729	6.549297333	H
atom	9.793704987	1.512081623	6.506409168	H
atom	11.060163498	2.895600557	1.603946090	H
atom	15.385080338	9.471977234	1.518361092	H
atom	1.205479383	8.366841316	3.979180813	H
atom	11.527773857	7.482556820	5.545361996	H
atom	15.171949387	10.978178978	4.260599136	H
atom	14.050427437	10.394389153	8.019656181	H
atom	14.291021347	5.931713104	9.363131523	H
atom	0.900200784	2.445525646	6.529560566	H
atom	11.891165733	1.977434397	7.458921909	H
atom	14.624491692	6.306509972	3.784251213	H
atom	14.891366005	5.043858528	6.837665558	H
atom	12.047007561	6.384294510	8.961830139	H
atom	13.299839020	2.357843399	1.854180574	H
atom	0.707557142	3.485585213	2.730767727	H
atom	13.708123207	6.969356060	6.040124893	H
atom	3.407044411	0.659868002	3.931550503	H
atom	4.465775490	0.835983694	4.911517143	H
atom	3.617699862	1.958939433	4.548225880	H
atom	3.384016037	6.301141262	1.541311264	H
atom	4.471859932	5.341635227	1.450823426	H
atom	3.718941212	5.472107887	2.687070847	H
atom	8.262153625	8.759543419	6.186106205	H
atom	9.117040634	8.411655426	7.308787346	H
atom	9.310333252	7.766414165	6.020941257	H
atom	2.487536430	3.448588848	8.634564400	H
atom	3.957075834	3.947489023	8.856361389	H
atom	2.849439383	4.954220295	8.390272141	H
atom	10.581952095	1.585939050	4.272080421	H
atom	9.038311005	1.332493782	4.374899387	H
atom	10.009328842	9.054402351	4.065430641	H
atom	2.368445635	7.995385647	6.962151051	H
atom	3.869233608	8.169031143	6.543702126	H
atom	2.825569630	7.621520042	5.509962559	H
atom	8.285068512	5.307963371	1.571021676	H

atom	9.297240257	6.340587616	1.717533946	H
atom	9.477260590	5.050038338	2.361217499	H
atom	9.476662636	3.503717899	8.122301102	H
atom	8.171834946	4.033699036	8.810888290	H
atom	9.136493683	3.075797081	9.591566086	H
atom	3.641198158	1.079410434	4.680861950	N
atom	3.667090654	5.497622013	1.798779368	N
atom	9.086812019	8.525960922	6.426781178	N
atom	9.158017159	5.464378834	1.641682863	N

Supplementary Table 24 | Input geometry.in file of $[R\text{-}4\text{-}Cl\text{-}MBA]_2PbI_4$ *relaxed* structure

lattice_vector	16.11377573	2.20892986	1.03548801	
lattice_vector	-0.13789862	8.80471966	-0.02135525	
lattice_vector	0.08343573	0.06814966	9.01450975	
atom	0.009402570	8.778249741	0.004470721	Pb
atom	16.094482422	6.348917007	5.724276543	Pb
atom	16.133068085	3.576680183	7.396850109	I
atom	3.188407421	8.845372200	8.793029785	I
atom	16.097749710	7.955562592	8.551532745	I
atom	0.061980218	2.724033356	1.699029207	I
atom	12.908586502	2.287695408	1.132809520	I
atom	0.031266760	7.269839287	2.940779924	I
atom	3.163183928	4.554043770	4.839527607	I
atom	12.898142815	6.081489086	5.936013699	I
atom	9.489064217	2.967112303	8.396280289	Cl
atom	6.643370152	3.085846901	3.644300938	Cl
atom	6.752621174	7.620211124	0.720984876	Cl
atom	9.400296211	7.467508793	6.078805447	Cl
atom	2.726526022	3.634151697	8.140599251	N
atom	13.354749680	2.654462814	4.574980259	N
atom	13.285193443	5.709723473	9.462243080	N
atom	2.742007732	7.976603985	5.307196140	N
atom	2.900397062	2.667635441	8.491484642	H
atom	2.785728931	3.621407509	7.093244553	H
atom	1.754505157	3.892442942	8.383969307	H
atom	13.222191811	3.568910122	5.059195518	H
atom	13.290668488	2.802701712	3.539217472	H
atom	14.317650795	2.316410542	4.767773628	H
atom	12.930866241	6.072994232	8.551904678	H
atom	13.221272469	4.662211418	9.426271439	H
atom	14.288291931	5.961548805	9.504900932	H
atom	2.930415154	8.411932945	6.237332821	H
atom	2.787934542	6.935156345	5.421890736	H
atom	1.771374226	8.210488319	5.031823635	H
atom	2.348729134	5.411528587	1.157737851	H
atom	4.068523407	5.754540920	1.421545863	H
atom	3.433145761	4.132450581	1.759386420	H
atom	3.545785427	5.542203903	8.014233589	H
atom	4.883628845	2.385795593	0.742606044	H
atom	7.230393410	1.701139688	0.612712860	H
atom	8.114748955	5.183637142	7.187771320	H
atom	5.720536709	5.823460102	7.224174976	H
atom	12.326442719	2.228780270	7.067606926	H

atom	13.499801636	9.745870590	6.707902431	H
atom	11.762713432	9.376205444	6.775801182	H
atom	12.640327454	9.515060425	4.409985065	H
atom	10.965150833	3.903362751	5.641025543	H
atom	8.650474548	4.470858097	5.086805344	H
atom	8.236339569	9.602252007	2.884556770	H
atom	10.588443756	9.037166595	3.399158478	H
atom	14.324728966	7.058570862	2.516187906	H
atom	12.808235168	7.601377487	3.236273289	H
atom	13.301570892	8.223995209	1.642845869	H
atom	12.567664146	5.322139740	2.373938322	H
atom	11.519276619	8.439001083	9.425106049	H
atom	9.169606209	9.020473480	9.059531212	H
atom	7.944552898	5.246823311	1.832782269	H
atom	10.325187683	4.659838200	2.217733383	H
atom	2.386409044	1.050472260	3.342778444	H
atom	4.103179932	1.249486446	2.942635298	H
atom	3.510658741	1.731654167	4.540103436	H
atom	3.529497147	7.674898148	3.408524036	H
atom	5.156662464	1.042536259	6.119852543	H
atom	7.465886593	9.463056564	6.871379375	H
atom	7.819441319	6.165654182	4.127040863	H
atom	5.480620861	6.505149841	3.409633160	H
atom	3.359891415	4.991203785	1.081163645	C
atom	3.703233719	4.668117046	8.661173820	C
atom	5.124866009	4.180929184	8.496247292	C
atom	5.569756985	3.033905745	9.163164139	C
atom	6.900026321	2.636315346	9.098617554	C
atom	7.805260181	3.415748358	8.382869720	C
atom	7.389688492	4.561812878	7.707207680	C
atom	6.045101643	4.926545620	7.752222061	C
atom	12.497939110	10.129245758	6.472062111	C
atom	12.347954750	1.599413872	4.982720852	C
atom	10.951562881	2.011739254	4.570693970	C
atom	10.390740395	3.208917618	5.026499748	C
atom	9.079184532	3.545582533	4.714775085	C
atom	8.308941841	2.671728849	3.953152657	C
atom	8.847266197	1.480385542	3.470083714	C
atom	10.170533180	9.970083237	3.776533365	C
atom	13.290126801	7.338782310	2.288324118	C
atom	12.539875031	6.170489311	1.675755739	C
atom	11.095779419	6.482845783	1.351301908	C
atom	10.751023293	7.730486393	9.736193657	C
atom	9.419629097	8.073358536	9.532047272	C
atom	8.419784546	7.154829979	0.939814210	C
atom	8.734775543	5.934414387	1.535633445	C

atom	10.075695992	5.608236313	1.740983844	C
atom	3.405390739	0.985213041	3.744925737	C
atom	3.720656395	8.387134552	4.223610878	C
atom	5.138319016	8.189115524	4.714997292	C
atom	5.716578960	9.018863678	5.687007427	C
atom	7.024324417	8.812005997	6.120466232	C
atom	7.763774395	7.770444870	5.561984062	C
atom	7.220318794	6.951593399	4.576953411	C
atom	5.908293724	7.159972191	4.167059898	C

Supplementary Table 25 | Input geometry.in file of [S-4-NH_3-MBA] PbI_4 *experimental* structure

lattice_vector	23.0755004883	0.0000000000	0.0000000000	
lattice_vector	0.0000000000	8.4849004745	0.0000000000	
lattice_vector	0.0000000000	0.0000000000	9.0443000793	
atom	5.641036987	6.341614723	7.775113106	Pb
atom	17.178787231	6.385735989	1.269186735	Pb
atom	5.896713734	2.143285990	3.252962828	Pb
atom	17.434463501	2.099164724	5.791337013	Pb
atom	5.218062878	3.684398413	6.083376884	I
atom	16.755813599	0.558051884	2.960922956	I
atom	6.319686890	4.800502300	1.561227560	I
atom	17.857437134	7.926848412	7.483073235	I
atom	5.209986210	7.988279343	5.060557365	I
atom	16.747737885	4.739071369	3.983742714	I
atom	6.327763557	0.496620983	0.538407385	I
atom	17.865512848	3.745829344	8.505892754	I
atom	8.659312248	6.169540882	7.042706013	I
atom	20.197061539	6.557809830	2.001594305	I
atom	2.878437996	2.315359831	2.520555735	I
atom	14.416188240	1.927089930	6.523744106	I
atom	2.488231182	6.523021698	8.581321716	I
atom	14.025980949	6.204329014	0.462977886	I
atom	9.049519539	1.961878777	4.059172153	I
atom	20.587268829	2.280571461	4.985127926	I
atom	8.600238800	6.843072414	3.641235113	N
atom	20.137989044	5.884278297	5.403064728	N
atom	2.937510967	1.641828060	8.163385391	N
atom	14.475261688	2.600621700	0.880914927	N
atom	14.581409454	6.733617306	6.036165714	N
atom	3.043660164	5.993733406	3.008134127	N
atom	20.031841278	1.751283288	1.514015913	N
atom	8.494091034	2.491167545	7.530284405	N
atom	8.507937431	6.487096310	4.451812267	H
atom	20.045688629	6.240254402	4.592487335	H
atom	3.029813290	1.997803926	8.973962784	H
atom	14.567563057	2.244645834	0.070337571	H
atom	7.858500004	6.701654434	3.169457197	H
atom	19.396251678	6.025696278	5.874842644	H
atom	3.679250240	1.783246040	7.691607475	H
atom	15.217000008	2.459204674	1.352692842	H
atom	8.741207123	7.718612194	3.716149330	H
atom	20.278957367	5.008738518	5.328150749	H
atom	2.796542883	0.766288340	8.238299370	H

atom	14.334293365	3.476161957	0.806000829	H
atom	14.461600304	6.389172554	6.848226547	H
atom	2.923851252	6.338178158	2.196073532	H
atom	20.151651382	2.095727682	2.326076508	H
atom	8.613900185	2.146722555	6.718223572	H
atom	14.798871994	7.593434334	6.107778549	H
atom	3.261122942	5.133916378	2.936521530	H
atom	19.814378738	0.891465962	1.585628629	H
atom	8.276628494	3.350984335	7.458671570	H
atom	15.235991478	6.295303822	5.621945858	H
atom	3.698241234	6.432046890	3.422354221	H
atom	19.377260208	2.189596653	1.099795818	H
atom	7.839509010	2.052853107	7.944504261	H
atom	9.587224007	5.243829727	2.843500853	H
atom	21.124975204	7.483520985	6.200799465	H
atom	1.950525880	3.241070986	7.365650654	H
atom	13.488275528	1.001379371	1.678649187	H
atom	13.531427383	8.348378181	4.310685158	H
atom	1.993677258	4.378972054	4.733614922	H
atom	21.081823349	0.136521846	8.832835197	H
atom	9.544073105	4.105928898	0.211464733	H
atom	9.098484993	6.571326256	1.046362281	H
atom	20.636236191	6.156024456	7.997938156	H
atom	2.439265013	1.913574100	5.568511963	H
atom	13.977015495	2.328875780	3.475787878	H
atom	10.660396576	6.455846786	1.115116954	H
atom	22.198146820	6.271503925	7.929183006	H
atom	0.877353549	2.029053688	5.637267113	H
atom	12.415102959	2.213397264	3.407032967	H
atom	9.947363853	7.755860806	1.624184489	H
atom	21.485115051	4.971489906	7.420115948	H
atom	1.590386510	0.729039669	6.146334171	H
atom	13.128135681	3.513410568	2.897965670	H
atom	12.811264038	4.815664291	6.013636589	H
atom	1.273514152	7.911686420	3.030663490	H
atom	21.801986694	3.669235945	1.491486549	H
atom	10.264236450	0.573214233	7.552813530	H
atom	10.883790016	4.597899437	4.725221634	H
atom	22.421541214	8.129450798	4.319078445	H
atom	0.653959930	3.887000799	0.203072295	H
atom	12.191709518	0.355449885	8.841228485	H
atom	11.588239670	8.152029037	3.091197014	H
atom	0.050488442	4.575321198	5.953103065	H
atom	23.025011063	0.332871139	7.613347054	H
atom	11.487260818	3.909578562	1.430953026	H
atom	13.339947701	6.604646683	5.261973858	C

atom	1.802195787	6.122704029	3.782326460	C
atom	21.273303986	1.880253911	0.739823580	C
atom	9.735552788	2.362195730	8.304476738	C
atom	9.774782181	6.200765133	2.938493013	C
atom	21.312530518	6.526585579	6.105806828	C
atom	1.762968540	2.284135342	7.460643291	C
atom	13.300719261	1.958314300	1.583656907	C
atom	11.032397270	6.356887341	3.787752867	C
atom	22.570146561	6.370463371	5.256547451	C
atom	0.505353391	2.128013134	8.309903145	C
atom	12.043103218	2.114437103	0.734397173	C
atom	12.993813515	7.595683098	4.403669834	C
atom	1.456063628	5.131667614	4.640630722	C
atom	21.619436264	0.889217436	8.925819397	C
atom	10.081686974	3.353232861	0.118480317	C
atom	9.880928993	6.800647736	1.554715276	C
atom	21.418680191	5.926702976	7.489584446	C
atom	1.656820774	1.684252501	6.076865673	C
atom	13.194571495	2.558198214	2.967434883	C
atom	12.566917419	5.482942581	5.413013935	C
atom	1.029167533	7.244408131	3.631286383	C
atom	22.046333313	3.001957893	0.890863657	C
atom	10.508583069	1.240491986	8.153436661	C
atom	11.408527374	5.362457275	4.648770332	C
atom	22.946277618	7.364893436	4.395529747	C
atom	0.129222929	3.122443199	0.126619652	C
atom	11.666972160	1.120006561	8.917679787	C
atom	11.835424423	7.470954895	3.673794508	C
atom	0.297673851	5.256395817	5.370505333	C
atom	22.777826309	1.013945460	8.195944786	C
atom	11.240076065	3.228505373	0.848355412	C

Supplementary Table 26 | Input geometry.in file of [S-4-NH_3-MBA] PbI_4 *relaxed* structure

lattice_vector	23.19104716	-0.00000072	-0.00001590	
lattice_vector	-0.00005571	8.39915710	-0.00000210	
lattice_vector	-0.00010385	-0.00000157	9.01922748	
atom	5.649615765	6.295403957	7.712908268	Pb
atom	17.244342804	6.303306103	1.305963516	Pb
atom	5.946035862	2.103985071	3.203572035	Pb
atom	17.541519165	2.095874310	5.815821171	Pb
atom	5.225178242	3.595787764	6.025808334	I
atom	16.820230484	0.603798270	2.993297338	I
atom	6.370659351	4.803175449	1.516174078	I
atom	17.965848923	7.795292377	7.502520084	I
atom	5.157329082	7.832911491	4.903037071	I
atom	16.752399445	4.765656471	4.115601063	I
atom	6.438534260	0.566413462	0.393775523	I
atom	18.033638000	3.633129835	8.625314713	I
atom	8.706032753	6.099038124	6.944530010	I
atom	20.300764084	6.499668121	2.074479103	I
atom	2.890034199	2.300090790	2.434824228	I
atom	14.485362053	1.899395466	6.584043026	I
atom	2.482058764	6.496784210	8.512528419	I
atom	14.076493263	6.101798534	0.506255209	I
atom	9.114139557	1.902238965	4.003043175	I
atom	20.709365845	2.297091246	5.015983582	I
atom	8.622753143	6.766093731	3.552861214	N
atom	20.218225479	5.832478523	5.466007710	N
atom	2.972840548	1.632977724	8.062551498	N
atom	14.568317413	2.566492081	0.956416786	N
atom	14.570774078	6.713675976	6.086416245	N
atom	2.975256920	5.884909630	2.932621956	N
atom	20.215789795	1.685256124	1.576689005	N
atom	8.620262146	2.514089823	7.442177296	N
atom	8.533645630	6.434957027	4.545525551	H
atom	20.129001617	6.163613796	4.473344803	H
atom	3.062060356	1.964126229	0.035974421	H
atom	14.657411575	2.235360146	8.982967377	H
atom	7.759588242	6.494475842	3.039859533	H
atom	19.355108261	6.104155064	5.979069233	H
atom	3.835952044	1.904627681	7.549478531	H
atom	15.431490898	2.294850349	1.469401956	H
atom	8.685597420	7.809414387	3.595930099	H
atom	20.281019211	4.789151669	5.422971725	H
atom	2.910045385	0.589645565	8.105599403	H

atom	14.505461693	3.609804869	0.913366556	H
atom	14.457107544	6.351112366	7.068832397	H
atom	2.861657858	6.247481823	1.950188398	H
atom	20.329391479	2.047829866	2.559109926	H
atom	8.733918190	2.151540995	6.459747314	H
atom	14.816763878	7.725045681	6.186286926	H
atom	3.221241713	4.873520374	2.832734346	H
atom	19.969795227	0.673865855	1.676575661	H
atom	8.374240875	3.525448561	7.342318535	H
atom	15.377356529	6.211309433	5.634465694	H
atom	3.781854630	6.387230396	3.384581566	H
atom	19.409196854	2.187605143	1.124726415	H
atom	7.813690186	2.011719704	7.894119740	H
atom	9.590755463	5.099396229	2.777116537	H
atom	21.186326981	7.499141216	6.241667271	H
atom	2.004750490	3.299672604	7.286896229	H
atom	13.600350380	0.899813592	1.732200384	H
atom	13.666730881	0.052035764	4.307972431	H
atom	2.071202278	4.147366047	4.711016655	H
atom	21.119852066	8.346850395	8.817533493	H
atom	9.524318695	4.251638412	0.201366901	H
atom	9.048230171	6.566802502	0.877223432	H
atom	20.643726349	6.031787395	8.141603470	H
atom	2.547350168	1.832305908	5.386977673	H
atom	14.142886162	2.367228746	3.632073641	H
atom	10.819658279	6.440884113	0.953617811	H
atom	22.415159225	6.157661438	8.065260887	H
atom	0.775921464	1.958218336	5.463297844	H
atom	12.371460915	2.241318226	3.555710316	H
atom	9.995160103	7.897577763	1.543450952	H
atom	21.590671539	4.700973988	7.475432873	H
atom	1.600403547	0.501511157	6.053140640	H
atom	13.195927620	3.698006630	2.965856075	H
atom	12.849674225	4.620830059	6.097355843	H
atom	1.254106879	7.977774143	2.921725035	H
atom	21.936971664	3.778072596	1.587625742	H
atom	10.341381073	0.421228647	7.431261063	H
atom	10.823328972	4.401626110	4.682179928	H
atom	22.418802261	8.196803093	4.336598873	H
atom	0.772264242	3.997374296	0.172738865	H
atom	12.367763519	0.202032387	8.846389771	H
atom	11.622361183	8.247200012	2.922101736	H
atom	0.026905328	4.351394653	6.096971035	H
atom	23.164165497	0.151674643	7.431585789	H
atom	11.568707466	4.047633171	1.587207794	H
atom	13.351099968	6.564424038	5.287695408	C

atom	1.755573750	6.034162045	3.731330633	C
atom	21.435482025	1.834483027	0.777993143	C
atom	9.839948654	2.364841700	8.240891457	C
atom	9.829980850	6.169972420	2.859922647	C
atom	21.425508499	6.428548336	6.158890724	C
atom	1.765561104	2.229085922	7.369675159	C
atom	13.361109734	1.970391035	1.649386525	C
atom	11.071439743	6.318298817	3.717963219	C
atom	22.666965485	6.280148506	5.300847530	C
atom	0.524099112	2.080722094	8.227713585	C
atom	12.119638443	2.118720770	0.791361213	C
atom	13.027189255	7.575900078	4.391970158	C
atom	1.431667924	5.022686481	4.627056122	C
atom	21.759391785	0.822995424	8.901495934	C
atom	10.163864136	3.376332760	0.117366433	C
atom	9.930478096	6.804010868	1.482272983	C
atom	21.525991440	5.794544697	7.536581516	C
atom	1.665080309	1.595081329	5.991988182	C
atom	13.260623932	2.604439020	3.027035713	C
atom	12.572450638	5.419394493	5.408575535	C
atom	0.976890445	7.179196358	3.610482693	C
atom	22.214168549	2.979498625	0.898847580	C
atom	10.618611336	1.219804764	8.120017052	C
atom	11.434477806	5.302499294	4.610887051	C
atom	23.029962540	7.295923233	4.407880306	C
atom	0.161096841	3.096511602	0.101450346	C
atom	11.756608009	1.102915049	8.917675972	C
atom	11.881977081	7.451903343	3.616425991	C
atom	0.286476672	5.146689415	5.402635098	C
atom	22.904586792	0.946974576	8.125922203	C
atom	11.309086800	3.252331495	0.892897785	C

Supplementary Table 27 | Input geometry.in file of [S-MBA]$_2$PbI$_4$ *experimental* structure

lattice_vector	-28.8650930286	-0.0123918327	-0.0172796207	
lattice_vector	0.0000000000	0.0009746838	9.3126601663	
lattice_vector	0.0000000000	8.9034404755	0.0000000000	
atom	0.039256528	5.360250473	0.061952688	Pb
atom	28.825836182	0.921363354	4.611657143	Pb
atom	14.471802711	8.002114296	9.259394646	Pb
atom	14.393290520	3.549885750	4.726875305	Pb
atom	28.855567932	3.661950350	2.973764181	I
atom	14.423021317	0.809324205	6.364803791	I
atom	14.442070961	5.261183739	7.621466160	I
atom	0.009525481	8.101156235	1.699845433	I
atom	0.000577302	2.635303736	7.409711838	I
atom	14.433124542	1.823587656	1.911589146	I
atom	14.431969643	6.275395393	2.762020826	I
atom	28.864515305	7.099327087	6.576558113	I
atom	3.202870607	5.482033253	9.048513412	I
atom	17.635417938	7.883048058	0.276619822	I
atom	11.229675293	3.429009199	4.396989822	I
atom	25.662221909	1.039522529	4.937755108	I
atom	25.726303101	5.533281803	9.052592278	I
atom	11.293756485	7.838746548	0.282229364	I
atom	17.571336746	3.390151739	4.391380787	I
atom	3.138790369	1.071434617	4.933677673	I
atom	21.692117691	8.554015160	9.185023308	C
atom	7.259571075	4.814550877	0.144966990	C
atom	21.605522156	0.369446903	4.528642654	C
atom	7.172976017	4.095602989	4.801245213	C
atom	21.262029648	0.640363157	8.342902184	C
atom	6.829481602	3.824390888	0.986573994	C
atom	22.035610199	8.282919884	3.687035799	C
atom	7.603065014	5.085937977	5.643366814	C
atom	23.926275253	0.544542968	9.141661644	C
atom	9.493728638	3.922499418	0.191005230	C
atom	19.371364594	8.378909111	4.482604504	C
atom	4.938817501	4.987663269	4.844608784	C
atom	23.005479813	8.488649368	0.266144961	C
atom	8.572932243	4.881042957	9.065418243	C
atom	20.292160034	0.433919311	4.920850754	C
atom	5.859613895	4.030002594	4.407464504	C
atom	22.151073456	1.574667692	7.886183262	C
atom	7.718525887	2.890849829	1.444357753	C
atom	21.146566391	7.348520756	3.229251862	C

atom	6.714020729	6.019575119	6.100086689	C
atom	23.473093033	1.528978467	8.277173996	C
atom	9.040547371	2.937675238	1.054948807	C
atom	19.824546814	7.394292355	3.618661165	C
atom	5.391999722	5.972667694	5.709094524	C
atom	23.773290634	5.457891941	5.382980347	C
atom	9.340744019	7.912459373	3.949503183	C
atom	19.524349213	3.464771986	0.724106431	C
atom	5.091802597	0.998490393	8.603289604	C
atom	25.513854980	5.895609856	3.579228878	C
atom	11.081309319	7.476235867	5.755338669	C
atom	17.783784866	3.027651787	8.230931282	C
atom	3.351237297	1.434116602	1.094381571	C
atom	25.204999924	5.741603851	5.070000648	C
atom	10.772452354	7.629977703	4.264196873	C
atom	18.092639923	3.180995226	0.409412861	C
atom	3.660093784	1.281038165	8.916269302	C
atom	23.068983078	6.331996441	6.229079247	C
atom	8.636435509	7.037749767	3.102560759	C
atom	20.228656769	2.590844870	1.571048856	C
atom	5.796110630	1.873022676	7.757190704	C
atom	23.094959259	4.389131069	4.828470230	C
atom	8.662414551	0.077197671	4.503200531	C
atom	20.202680588	4.533418179	0.170409128	C
atom	5.770132065	8.833867073	9.157799721	C
atom	21.726755142	6.112419605	6.461091995	C
atom	7.294209003	7.256174564	2.868940830	C
atom	21.570884705	2.810470343	1.804669142	C
atom	7.138337612	1.654549599	7.525177956	C
atom	21.761392593	4.207845688	5.087495804	C
atom	7.328846931	0.257337838	4.242578983	C
atom	21.536247253	4.714757442	0.431030780	C
atom	7.103699207	8.653673172	8.898774147	C
atom	21.068632126	5.033871651	5.893557549	C
atom	6.636085033	8.334156990	3.435687780	C
atom	22.229007721	3.888900518	1.237921953	C
atom	7.796461582	0.576685309	8.092712402	C
atom	25.334892273	0.504160762	0.338315606	C
atom	10.902345657	3.964090824	8.996039391	C
atom	17.962747574	8.418421745	4.990232468	C
atom	3.530201197	4.946939945	4.335294247	C
atom	25.825597763	1.783854127	0.895506442	C
atom	11.393052101	2.684819221	8.439434052	C
atom	17.472042084	7.138845444	5.546835899	C
atom	3.039494276	6.226094723	3.778103590	C
atom	21.088953018	7.917436600	0.180475950	H

atom	6.656405926	5.450610161	9.148793221	H
atom	22.208686829	1.005114317	4.837475777	H
atom	7.776140690	3.460453033	4.493133545	H
atom	20.371019363	0.676965058	8.082638741	H
atom	5.938473225	3.787024260	1.245770693	H
atom	22.926620483	8.246266365	3.427838802	H
atom	8.494073868	5.123359203	5.903630733	H
atom	23.284490585	7.802951336	0.828312397	H
atom	8.851943016	5.566980362	8.503583908	H
atom	20.013149261	1.119734883	5.482684135	H
atom	5.580604553	3.343947887	3.845297337	H
atom	21.862104416	2.244271755	7.309239864	H
atom	7.429557800	2.220998049	2.020955324	H
atom	21.435535431	6.678796291	2.652654409	H
atom	7.002989292	6.689548016	6.677030087	H
atom	24.067771912	2.167363167	7.956719398	H
atom	9.635225296	2.299800634	1.376116514	H
atom	19.229867935	6.755840302	3.297492743	H
atom	4.797320843	6.610610008	6.029551029	H
atom	25.940885544	3.874878645	5.255054951	H
atom	11.508339882	0.593893290	4.080023289	H
atom	17.356754303	5.047758102	0.593586445	H
atom	2.924207211	8.317082405	8.731215477	H
atom	25.969577789	4.577781677	6.526809216	H
atom	11.537031174	8.794453621	2.808303595	H
atom	17.328062057	4.345122337	1.865306020	H
atom	2.895515203	0.116254903	7.459460735	H
atom	26.967531204	4.871044159	5.511749268	H
atom	12.534983635	8.502049446	3.824558258	H
atom	16.330108643	4.051646233	0.849051476	H
atom	1.897562265	0.408873796	8.474520683	H
atom	26.446430206	6.096477032	3.464775562	H
atom	12.013882637	7.276169300	5.870909214	H
atom	16.851209641	2.826760292	8.115362167	H
atom	2.418663979	1.634207726	1.208834291	H
atom	24.986635208	6.610158443	3.214406729	H
atom	10.554087639	6.761234760	6.119529724	H
atom	18.311004639	2.313027143	7.866740227	H
atom	3.878458500	2.149193764	1.459203362	H
atom	25.305046082	5.077949524	3.122093678	H
atom	10.872499466	8.293716431	6.212223530	H
atom	17.992593765	3.845216513	7.774046421	H
atom	3.560047388	0.616730571	1.551515579	H
atom	25.439325333	6.586250782	5.508385181	H
atom	11.006779671	6.785531044	3.826092720	H
atom	17.858314514	2.336440086	0.847517014	H

atom	3.425766945	2.125392437	8.477884293	H
atom	23.503087997	7.050412178	6.628973007	H
atom	9.070537567	6.319706917	2.703186989	H
atom	19.794553757	1.872513294	1.970423102	H
atom	5.362008572	2.590980768	7.357296944	H
atom	23.546497345	3.790742159	4.277655125	H
atom	9.113950729	0.675974071	5.054556847	H
atom	19.751142502	5.132665634	8.931713104	H
atom	5.318595409	8.234231949	0.395954728	H
atom	21.257091522	6.698719025	7.008377075	H
atom	6.824545383	6.669471741	2.321093798	H
atom	22.040548325	2.224286318	2.352516174	H
atom	7.608001232	2.241136312	6.977892876	H
atom	21.320535660	3.489525795	4.693865299	H
atom	6.887989998	0.975279152	4.635681629	H
atom	21.977104187	5.432994366	0.037927970	H
atom	7.544556618	7.935813904	9.292405128	H
atom	20.165674210	4.883906364	6.065003395	H
atom	5.733127117	8.483347893	3.263161182	H
atom	23.131965637	4.038901806	1.410448551	H
atom	8.699419975	0.427458704	7.921266556	H
atom	25.389303207	8.724580765	1.038697720	H
atom	10.956756592	4.647156239	8.295722008	H
atom	17.908336639	0.198148414	5.690549374	H
atom	3.475790262	4.263726711	3.634912252	H
atom	26.042490005	0.552406311	7.793829918	H
atom	11.609944344	3.916453362	1.541369200	H
atom	17.255149841	8.370762825	3.132240534	H
atom	2.822602034	4.993992329	6.192440033	H
atom	26.216461182	8.144382477	8.395542145	H
atom	11.783914566	5.228065968	0.939866185	H
atom	17.081178665	0.778912008	3.733743191	H
atom	2.648632526	3.682253122	5.590727806	H
atom	27.103803635	0.332095563	8.761995316	H
atom	12.671257973	4.137673855	0.574475110	H
atom	16.193836212	8.591276169	4.099134445	H
atom	1.761290312	4.772567272	5.224274158	H
atom	25.638494492	2.494773149	0.277536750	H
atom	11.205947876	1.973738670	9.057179451	H
atom	17.659145355	6.427796841	4.929090500	H
atom	3.226597786	6.937304974	4.396072865	H
atom	26.772634506	1.727133393	1.040475488	H
atom	12.340087891	2.742352247	8.295598984	H
atom	16.525005341	7.195597172	5.690670967	H
atom	2.092459440	6.168531418	3.633134127	H
atom	25.383848190	1.960600734	1.729228020	H

atom	10.951300621	2.507693052	7.605183601	H
atom	17.913791656	6.962273121	6.381086349	H
atom	3.481245756	6.403047085	2.944381952	H
atom	26.117136002	4.651384830	5.652587891	N
atom	11.684589386	8.720978737	3.682701588	N
atom	17.180503845	4.271336079	0.990908027	N
atom	2.747956991	0.189914718	8.333682060	N
atom	26.270120621	0.118120588	8.536809921	N
atom	11.837574005	4.350934029	0.798662603	N
atom	17.027519226	8.805203438	3.874947548	N
atom	2.594971895	4.559355259	5.449459553	N

Supplementary Table 28 | Input geometry.in file of [S-MBA]$_2$PbI$_4$ *relaxed* structure

lattice_vector	-28.82929235	0.00114963	-0.01873775	
lattice_vector	-0.00126745	0.00055913	9.18552508	
lattice_vector	-0.00207826	8.80819805	-0.00043968	
atom	0.03964829	5.31907260	0.06237008	Pb
atom	14.45392082	7.89340709	9.11360039	Pb
atom	14.37615526	3.48918573	4.66497955	Pb
atom	28.79326708	0.91395803	4.53042484	Pb
atom	28.80628490	3.73065964	2.94992950	I
atom	14.38963528	0.67250853	6.24575994	I
atom	14.44175324	5.07682068	7.53316930	I
atom	0.02537187	8.13582409	1.64280388	I
atom	0.00765125	2.59450395	7.40943394	I
atom	14.42302549	1.81007891	1.76709793	I
atom	14.40842563	6.21432680	2.82643768	I
atom	28.82418036	6.99749753	6.36903489	I
atom	3.23363892	5.47200934	8.91382496	I
atom	17.64940543	7.74048247	0.26929687	I
atom	11.18161780	3.33617560	4.32364180	I
atom	25.59841359	1.06712216	4.86463418	I
atom	25.65810670	5.51377854	8.92348237	I
atom	11.24460167	7.69829372	0.26358135	I
atom	17.58635334	3.29449321	4.32902076	I
atom	3.17385677	1.10998590	4.85453368	I
atom	21.60644866	8.46602159	9.06474630	C
atom	7.19463300	4.74685038	0.12031824	C
atom	21.63906933	0.34258177	4.47258291	C
atom	7.22300822	4.06113635	4.71326140	C
atom	21.16037769	0.66140898	8.21118859	C
atom	6.74568034	3.74353171	0.97430759	C
atom	22.08569648	8.14716255	3.61839744	C
atom	7.67142300	5.06454889	5.56735199	C
atom	23.84425385	0.59151034	8.99389635	C
atom	9.42998546	3.81218160	0.19206006	C
atom	19.40140055	8.21672087	4.40026643	C
atom	4.98761986	4.99568353	4.78409999	C
atom	22.94796796	8.42359803	0.25417964	C
atom	8.53371624	4.78862292	8.93110430	C
atom	20.29866481	0.38426849	4.84712289	C
atom	5.88283727	4.01980402	4.33803518	C
atom	22.04669923	1.63086686	7.74482993	C
atom	7.63141848	2.77370162	1.44076298	C
atom	21.19972046	7.17750227	3.15194183	C

atom	6.78540771	6.03435221	6.03347344	C
atom	23.38109628	1.60190856	8.13982384	C
atom	8.96596472	2.80199728	1.04595732	C
atom	19.86514543	7.20626997	3.54654485	C
atom	5.45106924	6.00593358	5.63818558	C
atom	23.69531024	5.41531845	5.33610722	C
atom	9.27945905	7.79663332	3.85007573	C
atom	19.55113705	3.39264687	0.74302860	C
atom	5.13721388	1.01161877	8.44243847	C
atom	25.43231280	5.86299163	3.52133634	C
atom	11.01662555	7.34832830	5.66458250	C
atom	17.81369899	2.94512009	8.11330090	C
atom	3.40043302	1.45933441	1.07076637	C
atom	25.14479322	5.70048152	5.00631746	C
atom	10.72885930	7.51149709	4.17977886	C
atom	18.10184492	3.10751623	0.41279145	C
atom	3.68759768	1.29680911	8.77156800	C
atom	22.98873521	6.30138438	6.15804737	C
atom	8.57296462	6.91073071	3.02794629	C
atom	20.25737931	2.50647400	1.56505472	C
atom	5.84405647	1.89733857	7.62032783	C
atom	23.01733407	4.32731085	4.77075034	C
atom	8.60363566	0.07651005	4.41582505	C
atom	20.22932422	4.48070687	0.17796927	C
atom	5.81283511	8.73193511	9.00788913	C
atom	21.63193821	6.10637280	6.40679376	C
atom	7.21628799	7.10601463	2.77895779	C
atom	21.61411463	2.70134767	1.81408031	C
atom	7.20084699	1.70199889	7.37187358	C
atom	21.65916715	4.13633106	5.01736143	C
atom	7.24559849	0.26777409	4.16894832	C
atom	21.58743042	4.67153898	0.42484224	C
atom	7.17096184	8.54054549	8.76144334	C
atom	20.96613911	5.02367946	5.83887065	C
atom	6.55056730	8.18881815	3.34679965	C
atom	22.28016129	3.78401301	1.24634342	C
atom	7.86631331	0.61926935	7.94011783	C
atom	25.27559328	0.55983906	0.29641337	C
atom	10.85907369	3.84383755	8.89056018	C
atom	17.97107734	8.24783685	4.88760335	C
atom	3.55755754	4.96466063	4.29625366	C
atom	25.77004636	1.86754709	0.88565315	C
atom	11.35497538	2.53612835	8.30254777	C
atom	17.47597973	6.94018336	5.47648398	C
atom	3.06255367	6.27232292	3.70742973	C
atom	20.90326377	7.73298008	0.27063880	H

atom	6.48943918	5.48017391	8.91423371	H
atom	22.34322428	1.07522061	4.86412154	H
atom	7.92721975	3.32840070	4.32198148	H
atom	20.10781472	0.68896782	7.93394747	H
atom	5.69307920	3.71644884	1.25150613	H
atom	23.13835526	8.11971634	3.34141481	H
atom	8.72391307	5.09169345	5.84487534	H
atom	23.30954467	7.63043187	0.90754953	H
atom	8.89537372	5.58205803	8.27804769	H
atom	19.93684440	1.17763070	5.50016691	H
atom	5.52122011	3.22652391	3.68481640	H
atom	21.69745599	2.44096063	7.10709636	H
atom	7.28149929	1.96380482	2.07841751	H
atom	21.54940259	6.36741298	2.51441265	H
atom	7.13492317	6.84432112	6.67122456	H
atom	24.04929612	2.39427924	7.80058559	H
atom	9.63361368	2.00919836	1.38521999	H
atom	19.19724965	6.41367500	3.20727604	H
atom	4.78316649	6.79864146	5.97720353	H
atom	25.81630958	3.65808414	5.24922782	H
atom	11.40196267	0.74606455	3.93826933	H
atom	17.42993780	5.15002266	0.65442958	H
atom	3.01511161	8.06224059	8.52817006	H
atom	25.98055979	4.64524484	6.62279398	H
atom	11.56428252	8.56776368	2.56376738	H
atom	17.26596929	4.16361266	2.02860949	H
atom	2.85209950	0.24118375	7.15513067	H
atom	27.03350928	4.80848763	5.33098745	H
atom	12.61737290	8.40396684	3.85505253	H
atom	16.21299609	3.99909249	0.73732590	H
atom	1.79927961	0.40359948	8.44718502	H
atom	26.46430708	6.19331465	3.34690640	H
atom	12.04877580	7.01830349	5.83852228	H
atom	16.78174665	2.61486946	7.93859619	H
atom	2.36823742	1.78947093	1.24432232	H
atom	24.76272101	6.62444593	3.10866595	H
atom	10.34733920	6.58645521	6.07704571	H
atom	18.48346882	2.18350971	7.70115020	H
atom	4.06953472	2.22115048	1.48356918	H
atom	25.27106154	4.93305411	2.96218102	H
atom	10.85522762	8.27795468	6.22421913	H
atom	17.97529127	3.87489901	7.55396641	H
atom	3.56146810	0.52968438	1.63046012	H
atom	25.44941172	6.61554671	5.53303205	H
atom	11.03371738	6.59681867	3.65252284	H
atom	17.79688219	2.19267070	0.93971527	H

atom	3.38307065	2.21156967	8.24429132 H
atom	23.49372562	7.16963271	6.58252786 H
atom	9.07790257	6.04242008	2.60349828 H
atom	19.75215593	1.63825483	1.98936830 H
atom	5.33933092	2.76556435	7.19550286 H
atom	23.54325510	3.62823968	4.11949104 H
atom	9.12953283	0.77550128	5.06714259 H
atom	19.70229807	5.18049732	8.71235562 H
atom	5.28794602	8.03290371	0.47410139 H
atom	21.09262486	6.82773577	7.01712616 H
atom	6.67697788	6.38477823	2.16851206 H
atom	22.15314955	1.97988071	2.42447831 H
atom	7.74051589	2.42309790	6.76152643 H
atom	21.12748250	3.31719724	4.53694897 H
atom	6.71395224	1.08698921	4.64928637 H
atom	22.11799821	5.49129579	9.13014897 H
atom	7.70373998	7.72097047	0.05665552 H
atom	19.89960455	4.87707692	6.00346594 H
atom	5.48412475	8.33569150	3.18200835 H
atom	23.34667914	3.93050525	1.41102481 H
atom	8.93280894	0.47236315	7.77557672 H
atom	25.37631303	8.57229159	1.04743269 H
atom	10.96235152	4.63919816	8.13872542 H
atom	17.86994779	0.23533050	5.63952736 H
atom	3.45462032	4.16903144	3.54468635 H
atom	25.97882228	0.57014055	7.43871610 H
atom	11.56517415	3.83279017	1.74814126 H
atom	17.26634500	8.23697032	2.84448824 H
atom	2.85287448	4.97465451	6.33945098 H
atom	26.08538885	7.88767584	8.23218651 H
atom	11.66971446	5.32478596	0.95601293 H
atom	17.16194304	0.92041095	3.63741436 H
atom	2.74587987	3.48349604	5.54624481 H
atom	27.17859911	0.29919538	8.58514948 H
atom	12.76396107	4.10897400	0.60119978 H
atom	16.06716508	8.51112952	3.99135273 H
atom	1.65346513	4.70169617	5.19295743 H
atom	25.72791172	2.69605275	0.17007806 H
atom	11.31270145	1.70794602	9.01847942 H
atom	17.51827870	6.11168716	4.76090773 H
atom	3.10468445	7.10073490	4.42310377 H
atom	26.80080535	1.77417124	1.24805437 H
atom	12.38612662	2.63014592	7.94136639 H
atom	16.44498013	7.03382870	5.83820158 H
atom	2.03167592	6.17860212	3.34546093 H
atom	25.14145072	2.12603012	1.74552239 H

atom	10.72777124	2.27668123	7.44201025	H
atom	18.10381583	6.68144217	6.33677166	H
atom	3.69066695	6.53113995	2.84730578	H
atom	26.04641414	4.62284930	5.57650841	N
atom	11.63021058	8.58970243	3.61004807	N
atom	17.20006528	4.18539917	0.98232586	N
atom	2.78643485	0.21870477	8.20142333	N
atom	26.18796542	0.11480264	8.35344803	N
atom	11.77288115	4.28985898	0.83400967	N
atom	17.05802123	8.69352082	3.75875747	N
atom	2.64427730	4.51865052	5.42494699	N

Supplementary Table 29 | Input geometry.in file of [S-4-NO_2-MBA]$_2PbBr_4 \cdot H_2O$ *experimental* structure

lattice_vector	18.1396270419	-0.5412233354	0.0000000000
lattice_vector	0.0000000000	8.8284997940	0.0000000000
lattice_vector	0.0000000000	0.0000000000	8.0875997543

atom	8.975125313	6.479924202	6.762527466	Pb
atom	9.164502144	1.807352185	2.718727112	Pb
atom	11.918279648	6.246705055	6.678901672	Br
atom	6.221348286	2.040571213	2.635101557	Br
atom	9.004510880	3.882850409	0.231063172	Br
atom	9.135116577	4.404426098	4.274863243	Br
atom	5.955421448	6.405458450	7.144666195	Br
atom	12.184206009	1.881817937	3.100866318	Br
atom	9.129674911	7.910209179	1.202545047	Br
atom	9.009952545	0.377067357	5.246345043	Br
atom	14.294026375	7.458249092	3.694415331	C
atom	3.845601320	0.829027057	7.738215446	C
atom	13.766162872	2.967932224	7.251341820	C
atom	4.373464584	5.319344521	3.207542181	C
atom	14.312165260	3.659687519	0.238584250	C
atom	3.827461958	4.627588749	4.282383919	C
atom	15.605521202	4.164934158	0.155281648	C
atom	2.534105778	4.122342587	4.199081421	C
atom	16.349246979	3.978533506	7.083928585	C
atom	1.790380955	4.308743000	3.040128946	C
atom	15.803243637	3.287661076	6.009086609	C
atom	2.336383581	4.999615192	1.965286970	C
atom	14.509887695	2.782415152	6.093197823	C
atom	3.629739523	5.504861355	2.049398422	C
atom	16.213199615	-0.157091483	0.085728325	C
atom	1.926428080	8.444367409	4.129528046	C
atom	15.240915298	7.679843903	0.050142761	C
atom	2.898712397	0.607432544	4.093942642	C
atom	14.571562767	7.320189476	1.214757562	C
atom	3.568064213	0.967086732	5.258557320	C
atom	14.876308441	7.952928543	2.414148092	C
atom	3.263318777	0.334347636	6.457947731	C
atom	15.848592758	0.115993902	2.449733734	C
atom	2.291034460	8.171282768	6.493533611	C
atom	16.517944336	0.474765152	1.285928726	C
atom	1.621682525	7.812511444	5.329728603	C
atom	14.747517586	6.019798756	4.069680691	C
atom	3.392109871	2.267477751	0.025880758	C

atom	12.363969803	2.451807499	7.383169651	C
atom	5.775657654	5.835468769	3.339369774	C
atom	12.302295685	0.952803135	7.396918774	C
atom	5.837331772	7.334473133	3.353118896	C
atom	14.562075615	8.067071915	4.414139271	H
atom	3.577551842	0.220204324	0.370340496	H
atom	12.452509880	7.185894489	4.410467148	H
atom	5.687118053	1.101381779	0.366667211	H
atom	12.526229858	6.851204872	2.998089552	H
atom	5.613398075	1.436071515	7.041889668	H
atom	12.482112885	8.243177414	3.413703203	H
atom	5.657514572	0.044098966	7.457502842	H
atom	13.814051628	3.784022331	1.013877988	H
atom	4.325575829	4.503253937	5.057677746	H
atom	15.970871925	4.627125263	0.873946071	H
atom	2.168755531	3.660150766	4.917746067	H
atom	16.301521301	3.162730217	5.234173298	H
atom	1.838106513	5.124546528	1.190374017	H
atom	14.144682884	2.319609642	5.374072552	H
atom	3.994945049	5.967666626	1.330273271	H
atom	15.036572456	7.256433964	7.335064888	H
atom	3.103054762	1.030842423	3.291265011	H
atom	13.920060158	6.656717300	1.190688372	H
atom	4.219567776	1.630559087	5.234488487	H
atom	16.052808762	0.538745642	3.252629995	H
atom	2.086818457	7.748530865	7.296430111	H
atom	17.169338226	1.138471246	1.309414625	H
atom	0.970288336	7.148805141	5.353214264	H
atom	14.285573006	5.735301971	4.861625671	H
atom	3.854054213	2.551974535	0.817825735	H
atom	15.693697929	6.014045238	4.231447697	H
atom	2.445929289	2.273231030	0.187648028	H
atom	14.542757034	5.421226501	3.347417116	H
atom	3.596870899	2.866049767	7.391217232	H
atom	11.990583420	2.784075737	0.138557106	H
atom	6.149043560	5.503201008	4.182356834	H
atom	10.651407242	2.778512955	6.435157776	H
atom	7.488220215	5.508763313	2.391357183	H
atom	11.739858627	2.542258024	5.501508713	H
atom	6.399768829	5.745018482	1.457708240	H
atom	11.618123055	3.829580784	6.165007114	H
atom	6.521503925	4.457695484	2.121207952	H
atom	12.711398125	0.608451366	6.600379467	H
atom	5.428229809	7.678824902	2.556580067	H
atom	11.384339333	0.671564996	7.432544231	H
atom	6.755287647	7.615711212	3.388744116	H

atom	12.768392563	0.620523095	0.079371899	H
atom	5.371234894	7.666753769	4.123171806	H
atom	11.370282173	4.843750954	2.544552565	H
atom	6.769345760	3.443525791	6.588352680	H
atom	12.003154755	5.023412228	1.322646141	H
atom	6.136472702	3.263864279	5.366446018	H
atom	12.782995224	7.431822300	3.621627569	N
atom	5.356632233	0.855454266	7.665427208	N
atom	16.958738327	0.206470579	6.982833385	N
atom	1.180889964	8.080805779	2.939033508	N
atom	11.505965233	2.951498747	6.256567001	N
atom	6.633661747	5.335777760	2.212767124	N
atom	17.662555695	4.541452885	6.944013119	N
atom	0.477072448	3.745823860	2.900213480	N
atom	16.552410126	-0.265208125	5.930636883	O
atom	1.587216973	8.552484512	1.886836767	O
atom	17.898370743	0.995071173	7.098485947	O
atom	0.241257071	7.292205334	3.054686308	O
atom	11.778059959	5.402117252	2.049397469	O
atom	6.361567497	2.885159254	6.093197346	O
atom	17.976369858	5.375211239	7.731745243	O
atom	0.163256705	2.912065029	3.687945366	O
atom	0.206791475	4.815957069	6.089962959	O
atom	17.932836533	3.471319437	2.046162844	O

Supplementary Table 30 | Input geometry.in file of [S-4-NO_2-MBA]$_2$PbBr_4·H_2O *relaxed* structure

lattice_vector	18.18143862	-0.51477055	0.00020182
lattice_vector	0.11036920	8.82895089	-0.00151199
lattice_vector	-0.00187737	-0.00203195	7.90995217

atom	9.02681485	6.50760946	6.52354186 Pb
atom	9.26293772	1.80042617	2.57072988 Pb
atom	12.03998365	6.18337337	6.57571043 Br
atom	6.25183970	2.12317676	2.62293676 Br
atom	8.93989668	3.72707136	-0.00205316 Br
atom	9.35030392	4.58501372	3.95063647 Br
atom	6.02604755	6.52696539	6.97288821 Br
atom	12.26287900	1.78150324	3.02173365 Br
atom	9.28005712	7.90013317	1.20576084 Br
atom	9.00976834	0.40892141	5.16198976 Br
atom	14.45001788	7.44678198	3.65690629 C
atom	3.84525862	0.86605661	7.61174030 C
atom	13.88449488	2.90530532	7.10723553 C
atom	4.40027918	5.40014347	3.15258504 C
atom	14.40408402	3.62012977	0.28454756 C
atom	3.88981877	4.68497245	4.24179903 C
atom	15.66676278	4.19259371	0.21565343 C
atom	2.62637850	4.11361433	4.18081921 C
atom	16.40554672	4.02644245	6.95963239 C
atom	1.87627104	4.28276049	3.02168692 C
atom	15.94580543	3.26821178	5.88383574 C
atom	2.32858045	5.04294418	1.94424956 C
atom	14.67487874	2.71187727	5.96407993 C
atom	3.60183918	5.59617871	2.01542249 C
atom	16.31999649	-0.10194977	0.08373900 C
atom	1.97596406	8.42444987	4.03182673 C
atom	15.46097099	7.73245210	-0.01035053 C
atom	2.84114587	0.58398627	3.94244651 C
atom	14.78277241	7.35309725	1.14023854 C
atom	3.51887125	0.95988003	5.09441156 C
atom	15.07245706	7.95218483	2.37293266 C
atom	3.22268705	0.36343655	6.32677367 C
atom	15.91478492	0.14815656	2.42977051 C
atom	2.37429543	8.17303056	6.37891205 C
atom	16.59836268	0.54122355	1.28564829 C
atom	1.69100395	7.78353674	5.23336494 C
atom	14.98573024	6.07047421	4.05268242 C
atom	3.31416987	2.24536059	0.09838488 C

atom	12.47256620	2.37275246	7.20197190	C
atom	5.81243965	5.93365044	3.24181974	C
atom	12.37971064	0.85675395	7.20290256	C
atom	5.90509844	7.44960141	3.24012108	C
atom	14.68221644	8.16316932	4.45559374	H
atom	3.61260022	0.15167671	0.49973468	H
atom	12.55413512	7.12146025	4.49147257	H
atom	5.74317193	1.18818726	0.53763608	H
atom	12.58326147	6.69437150	2.86449690	H
atom	5.71343426	1.61483099	6.82079697	H
atom	12.57961412	8.35543921	3.33384059	H
atom	5.71359125	-0.04673022	7.28888110	H
atom	13.79402243	3.74602401	1.17812018	H
atom	4.50588534	4.55725843	5.13098770	H
atom	16.08863150	4.76362859	1.03706284	H
atom	2.21011005	3.54092796	5.00400286	H
atom	16.57391517	3.12776975	5.00738714	H
atom	1.69300482	5.18773885	1.07367220	H
atom	14.29176168	2.15101681	5.11015402	H
atom	3.97930869	6.15829900	1.15980812	H
atom	15.25366167	7.25031839	6.94774916	H
atom	3.05141337	1.06269881	2.99100382	H
atom	14.03277944	6.56889439	1.06222414	H
atom	4.27355274	1.73961421	5.01763312	H
atom	16.15583017	0.62308906	3.37983304	H
atom	2.12884854	7.69958351	7.32863437	H
atom	17.33306880	1.33929836	1.31931513	H
atom	0.95190645	6.98941176	5.26547329	H
atom	14.56574035	5.75422075	5.01552383	H
atom	3.73303145	2.55978878	1.06233193	H
atom	16.07701401	6.11956329	4.13812215	H
atom	2.22271217	2.19815379	0.18242326	H
atom	14.73092576	5.30921502	3.30377580	H
atom	3.57003844	3.00473629	7.26058165	H
atom	12.02639786	2.76924379	0.21292924	H
atom	6.26286167	5.54112419	4.16241461	H
atom	10.62927854	2.76760836	6.27989895	H
atom	7.65446395	5.53788962	2.31823633	H
atom	11.86048667	2.53554757	5.14676180	H
atom	6.42268353	5.76904681	1.18596627	H
atom	11.74754150	3.97690789	6.04655608	H
atom	6.53652514	4.32817788	2.08738188	H
atom	12.80352260	0.42690216	6.28718418	H
atom	5.47786665	7.87812355	2.32534740	H
atom	11.33922114	0.52369253	7.29193816	H
atom	6.94593571	7.78273053	3.32477767	H

atom	12.93957654	0.46372129	0.14627448	H
atom	5.35029700	7.84585660	4.09492727	H
atom	11.13955836	5.13077542	2.39856264	H
atom	7.15455263	3.18041549	6.35639294	H
atom	11.72047429	5.66654571	1.04751379	H
atom	6.57610417	2.64413573	5.00448593	H
atom	12.94634845	7.40432259	3.56740791	N
atom	5.34896658	0.90504084	7.52305775	N
atom	17.08612689	0.26240525	6.79644039	N
atom	1.20757991	8.06298174	2.83341369	N
atom	11.63241191	2.93805720	6.08614938	N
atom	6.65119652	5.36709552	2.12557530	N
atom	17.70074776	4.70338590	6.85262308	N
atom	0.57751942	3.61073636	2.92632924	N
atom	16.74142821	-0.23529071	5.71533375	O
atom	1.55722851	8.55850293	1.75268057	O
atom	18.03266865	1.03917590	6.92651558	O
atom	0.25593626	7.29246306	2.96175472	O
atom	11.97305577	5.41275010	1.96010844	O
atom	6.32190846	2.89761046	5.91665918	O
atom	17.97653855	5.56845162	7.68849687	O
atom	0.32291878	2.71982560	3.74142408	O
atom	0.25917856	4.90105266	5.91461879	O
atom	17.99459980	3.44425256	2.01999067	O

Supplementary Table 31 | Input geometry.in file of [R-4-Cl-MBA]$_2$PbBr$_4$ *experimental* structure

lattice_vector	35.5777015686	0.0000000000	0.0000000000	
lattice_vector	0.0000000000	7.9134998322	0.0000000000	
lattice_vector	0.0000000000	0.0000000000	8.8065996170	
atom	8.847106934	5.906240463	0.368380070	Pb
atom	26.635957718	5.964009285	8.438219070	Pb
atom	8.941744804	2.007259369	4.771679878	Pb
atom	26.730594635	1.949491024	4.034919739	Pb
atom	8.799432755	7.421042919	2.904944897	Br
atom	26.588283539	4.449206829	5.901654720	Br
atom	8.989417076	0.492457032	7.308244705	Br
atom	26.778268814	3.464293003	1.498354793	Br
atom	8.493820190	3.373920679	1.849738240	Br
atom	26.282670975	0.582829297	6.956861496	Br
atom	9.295030594	4.539579391	6.253037930	Br
atom	27.083881378	7.330670357	2.553561687	Br
atom	5.839724064	6.180047989	0.564062655	Br
atom	23.628574371	5.690201759	8.242536545	Br
atom	11.949127197	1.733452082	4.967362881	Br
atom	29.737977982	2.223298311	3.839237213	Br
atom	11.749535561	5.622146130	0.856529891	Br
atom	29.538387299	6.248103619	7.950069904	Br
atom	6.039315224	2.291353941	5.259829521	Br
atom	23.828165054	1.665395498	3.546769857	Br
atom	18.176649094	2.283044815	7.401066303	Cl
atom	0.387797683	1.673705101	1.405533314	Cl
atom	35.189903259	5.630455017	2.997766256	Cl
atom	17.401052475	6.239794731	5.808833122	Cl
atom	17.496402740	5.708798885	1.025968790	Cl
atom	35.285255432	6.161450863	7.780630589	Cl
atom	0.292448252	2.204700947	5.429268837	Cl
atom	18.081298828	1.752049088	3.377331018	Cl
atom	11.658812523	6.249290466	4.247423172	N
atom	29.447664261	5.620959282	4.559176445	N
atom	6.130038261	1.664209247	8.650723457	N
atom	23.918888092	2.292541265	0.155876696	N
atom	11.470251083	2.548938513	8.197182655	N
atom	29.259099960	1.407811522	0.609416723	N
atom	6.318599701	5.364561081	3.793883085	N
atom	24.107452393	6.505688667	5.012716293	N
atom	10.928082466	5.995528221	4.685912609	H
atom	28.716932297	5.874721527	4.120687008	H
atom	6.860768318	1.917971492	0.282612771	H
atom	24.649620056	2.038778543	8.523986816	H

atom	11.576308250	6.040493488	3.385890961	H
atom	29.365159988	5.829756260	5.420708656	H
atom	6.212542057	1.873006582	7.789190769	H
atom	24.001392365	2.083742857	1.017408729	H
atom	11.765260696	7.129034996	4.330143452	H
atom	29.554111481	4.741214752	4.476456165	H
atom	6.023589611	0.784465015	8.733443260	H
atom	23.812440872	3.172285318	0.073156513	H
atom	10.629727364	2.267186165	8.259022713	H
atom	28.418577194	1.689563751	0.547576785	H
atom	7.159123421	5.646313667	3.855722904	H
atom	24.947975159	6.223936081	4.950876713	H
atom	11.680622101	2.672626257	7.340591431	H
atom	29.469472885	1.284123659	1.466008186	H
atom	6.108229160	5.240873814	2.937291384	H
atom	23.897079468	6.629375935	5.869307995	H
atom	11.560404778	3.317339182	8.637574196	H
atom	29.349256516	0.639410734	0.169025064	H
atom	6.228445530	4.596160889	4.234274864	H
atom	24.017295837	7.274088860	4.572324753	H
atom	12.119580269	0.651099026	8.440385818	H
atom	29.908432007	3.305650949	0.366213650	H
atom	5.669270992	7.262400627	4.037086010	H
atom	23.458120346	4.607849121	4.769513607	H
atom	14.239654541	7.605767250	3.835582495	H
atom	32.028507233	4.264482498	4.971017361	H
atom	3.549196482	0.307732463	8.238882065	H
atom	21.338047028	3.649017572	0.567717314	H
atom	14.165048599	3.631085634	3.636887789	H
atom	31.953899384	0.325664371	5.169712067	H
atom	3.623802185	4.282414436	8.040187836	H
atom	21.412652969	7.587835312	0.766412079	H
atom	13.770171165	3.734784126	8.061217308	H
atom	31.559022903	0.221965700	0.745381773	H
atom	4.018679619	4.178715706	3.657917976	H
atom	21.807529449	7.691534042	5.148681641	H
atom	12.635741234	4.596485138	4.965134621	H
atom	30.424592972	7.273764610	3.841465235	H
atom	5.153109550	3.317014694	0.561834633	H
atom	22.941959381	0.639735758	8.244765282	H
atom	12.526623726	2.323458910	1.854846001	H
atom	30.315475464	1.633291006	6.951753616	H
atom	5.262226582	5.590040684	6.258145809	H
atom	23.051076889	6.280209064	2.548453808	H
atom	12.598774910	0.757456422	1.857849002	H
atom	30.387626648	3.199293375	6.948750496	H

atom	5.190075397	7.156043053	6.261148930	H
atom	22.978925705	4.714206696	2.545450926	H
atom	11.221065521	1.478265524	1.657877564	H
atom	29.009916306	2.478484631	7.148722172	H
atom	6.567785740	6.435234547	6.061177254	H
atom	24.356636047	5.435015202	2.745422125	H
atom	14.358875275	7.775116444	8.667957306	H
atom	32.147727966	4.095133305	0.138642296	H
atom	3.429975510	0.138383374	4.264657497	H
atom	21.218826294	3.818366528	4.541942120	H
atom	15.929559708	3.670573950	2.130686283	H
atom	33.718410492	0.286175907	6.675913334	H
atom	1.859291077	4.242925644	6.533986092	H
atom	19.648143768	7.627324104	2.272613525	H
atom	15.976380348	3.992170811	7.524120808	H
atom	33.765232086	7.878078938	1.282478690	H
atom	1.812470436	3.921329021	3.120820999	H
atom	19.601320267	0.035421383	5.685778618	H
atom	13.509778023	7.056958675	6.051384449	H
atom	31.298629761	4.813291073	2.755214930	H
atom	4.279072762	0.856541276	1.648084760	H
atom	22.067922592	3.100208759	7.158514977	H
atom	13.839833260	5.638962269	6.632699490	H
atom	31.628684998	6.231287479	2.173900366	H
atom	3.949017763	2.274537563	2.229398966	H
atom	21.737867355	1.682211876	6.577199936	H
atom	12.376450539	6.196056843	6.708664894	H
atom	30.165302277	5.674192905	2.097934723	H
atom	5.412399769	1.717443228	2.305364609	H
atom	23.201250076	2.239307165	6.501234531	H
atom	16.031633377	7.646569729	2.375958920	H
atom	33.820480347	4.223680019	6.430640221	H
atom	1.757218361	0.266930133	6.779259205	H
atom	19.546070099	3.689819813	2.027340889	H
atom	16.560388565	0.106705636	8.150428772	H
atom	34.349239349	3.850044250	0.656170964	H
atom	1.228462458	7.806794167	3.747128963	H
atom	19.017313004	4.063455582	5.059471130	H
atom	13.797033310	1.763127804	8.414706230	C
atom	31.585882187	2.193622112	0.391893685	C
atom	3.991817713	6.150372028	4.011406422	C
atom	21.780670166	5.719877720	4.795193195	C
atom	12.381039619	1.528888226	8.788986206	C
atom	30.169891357	2.427861929	0.017613200	C
atom	5.407811165	6.384611607	4.385686398	C
atom	23.196660995	5.485638142	4.420912743	C

atom	14.010499001	5.605132103	3.867858648	C
atom	31.799350739	6.265117645	4.938740730	C
atom	3.778352261	2.308367968	8.271159172	C
atom	21.567201614	1.648381948	0.535441160	C
atom	14.579741478	6.809566498	3.495339394	C
atom	32.368591309	5.060683250	5.311260223	C
atom	3.209108829	1.103933454	7.898639202	C
atom	20.997959137	2.852816582	0.907960415	C
atom	14.529932976	4.445012569	3.372927427	C
atom	32.318782806	7.425237179	5.433671951	C
atom	3.258917570	3.468487024	7.776227474	C
atom	21.047767639	0.488262832	1.030372262	C
atom	14.330698013	2.991302967	8.075652122	C
atom	32.119548798	0.965447068	0.730947733	C
atom	3.458152771	4.922196865	3.672352076	C
atom	21.247005463	6.948052883	5.134247780	C
atom	12.868454933	5.537867069	4.825136185	C
atom	30.657304764	6.332382679	3.981463432	C
atom	4.920396328	2.375632524	0.421835780	C
atom	22.709247589	1.581117392	8.384763718	C
atom	12.164015770	1.520974755	1.473344088	C
atom	29.952867508	2.435775280	7.333255768	C
atom	5.624835014	6.392525196	5.876643658	C
atom	23.413684845	5.477724552	2.929955721	C
atom	14.675801277	0.703510106	8.435841560	C
atom	32.464653015	3.253239870	0.370757848	C
atom	3.113049030	7.209990025	4.032541752	C
atom	20.901899338	4.660259724	4.774057865	C
atom	16.490264893	2.101034164	7.783273220	C
atom	34.279117584	1.855715632	1.023326874	C
atom	1.298586369	5.812465668	3.379972935	C
atom	19.087436676	6.057784081	5.426626205	C
atom	15.590148926	4.463213921	2.479938507	C
atom	33.378997803	7.407035828	6.326661110	C
atom	2.198702097	3.450285912	6.883238316	C
atom	19.987552643	0.506464064	1.923361421	C
atom	15.654188156	3.151155710	7.757733822	C
atom	33.443038940	0.805594265	1.048866034	C
atom	2.134662151	4.762343884	3.354433775	C
atom	19.923513412	7.107905865	5.452165604	C
atom	13.177981377	6.165407658	6.179590702	C
atom	30.966831207	5.704842091	2.627008915	C
atom	4.610869884	1.748092055	1.776290894	C
atom	22.399721146	2.208657265	7.030308723	C
atom	15.643515587	6.837264061	2.622605324	C
atom	33.432365417	5.032985687	6.183994293	C

atom	2.145335197	1.076235771	7.025905132	C
atom	19.934185028	2.880514622	1.780694366	C
atom	15.995735168	0.844370484	8.129371643	C
atom	33.784584045	3.112379551	0.677227497	C
atom	1.793115735	7.069129467	3.726072311	C
atom	19.581968307	4.801120281	5.080527306	C
atom	16.127372742	5.658152103	2.125913143	C
atom	33.916221619	6.212097645	6.680686474	C
atom	1.661478639	2.255347729	6.529212952	C
atom	19.450328827	1.701401830	2.277386665	C

Supplementary Table 32 | Input geometry.in file of $[R\text{-}4\text{-}Cl\text{-}MBA]_2PbBr_4$ *relaxed* structure

lattice_vector	34.78428368	-0.00361436	0.00441796	
lattice_vector	0.00184598	7.83897449	0.00005063	
lattice_vector	-0.00106749	0.00001633	8.85655343	
atom	8.61591393	5.82498182	-0.50777093	Pb
atom	26.00786154	5.93021001	9.36909117	Pb
atom	8.77693365	2.01439503	3.92071987	Pb
atom	26.16982785	1.90109201	4.94054396	Pb
atom	8.59165524	7.30556593	2.08133880	Br
atom	25.98313549	4.44937964	6.77983454	Br
atom	8.80067852	0.53410362	6.50982205	Br
atom	26.19395376	3.38200080	2.35222961	Br
atom	8.23182937	3.26000748	0.92356693	Br
atom	25.62251918	0.65618037	7.93784573	Br
atom	9.16112486	4.57930953	5.35263027	Br
atom	26.55496640	7.17529004	3.50856116	Br
atom	5.55019814	6.17940056	-0.29184433	Br
atom	22.94195146	5.57653840	9.15316831	Br
atom	11.84223478	1.65898661	4.13667348	Br
atom	29.23559755	2.25584483	4.72525194	Br
atom	11.54263641	5.47755767	0.02306366	Br
atom	28.93452113	6.27771458	8.83823877	Br
atom	5.85013445	2.36135148	4.45119043	Br
atom	23.24300115	1.55449731	4.40972142	Br
atom	18.16762845	2.03382405	7.79514494	Cl
atom	0.77598806	1.88346285	1.06244407	Cl
atom	34.00977707	5.80014605	3.36898949	Cl
atom	16.61677590	5.95429730	5.49249149	Cl
atom	17.69207414	5.64758597	0.52953011	Cl
atom	35.08413570	6.10468677	8.33300110	Cl
atom	-0.29854514	2.18959203	4.95551369	Cl
atom	17.09265462	1.72857272	3.90228306	Cl
atom	11.52688483	6.17108305	3.37283037	N
atom	28.91921947	5.58284422	5.48884850	N
atom	5.86567661	1.66703936	7.80101125	N
atom	23.25855265	2.24980072	1.06024610	N
atom	11.35966677	2.48014752	7.29571032	N
atom	28.75223082	1.43595052	1.56584816	N
atom	6.03348132	5.35824821	2.86722363	N
atom	23.42491838	6.39642236	5.99361662	N
atom	10.65498874	5.86589580	3.85409065	H
atom	28.04755324	5.88832412	5.00735061	H
atom	6.73842319	1.97244181	-0.57415962	H

atom	24.12927473	1.94429319	9.43565696	H
atom	11.41525482	5.98386084	2.35071537	H
atom	28.80743590	5.77038312	6.51089081	H
atom	5.97761617	1.85419285	6.77890829	H
atom	23.37005096	2.06223747	2.08234050	H
atom	11.61201575	7.20349762	3.51865645	H
atom	29.00392654	4.55034578	5.34331813	H
atom	5.78056662	0.63461332	7.94698536	H
atom	23.17381083	3.28231498	0.91455962	H
atom	10.36130316	2.18986535	7.31796854	H
atom	27.75402264	1.72672975	1.54406465	H
atom	7.03180798	5.64842225	2.88972303	H
atom	24.42302630	6.10534063	5.97190359	H
atom	11.61015887	2.51324284	6.28070446	H
atom	29.00305123	1.40215359	2.58074943	H
atom	5.78324799	5.32500518	1.85216012	H
atom	23.17415793	6.43045527	7.00853144	H
atom	11.43882245	3.42366944	7.73857956	H
atom	28.83109773	0.49256457	1.12256750	H
atom	5.95402250	4.41471147	3.31017012	H
atom	23.34630027	7.33983413	5.55023080	H
atom	11.87626758	0.48213767	7.53124995	H
atom	29.26949646	3.43388089	1.33091920	H
atom	5.51681425	7.35621647	3.10249480	H
atom	22.90714520	4.39867326	5.75894629	H
atom	14.06952994	7.68210155	3.06000344	H
atom	31.46146645	4.07072278	5.80228804	H
atom	3.32319242	0.15557855	7.48768816	H
atom	20.71632424	3.76204150	1.37247331	H
atom	14.08552191	3.37031252	2.86997487	H
atom	31.47693687	0.54361798	5.99142218	H
atom	3.30707183	4.46733337	7.29687130	H
atom	20.70053365	7.28923714	1.56213078	H
atom	13.65146051	3.79952901	7.66351059	H
atom	31.04338626	0.11580170	1.19809253	H
atom	3.74193679	4.03871023	3.23443288	H
atom	21.13408467	7.71707733	5.62588751	H
atom	12.37807077	4.36309726	3.95962830	H
atom	29.77125378	7.39034577	4.90189870	H
atom	5.01507850	3.47488273	-0.46912300	H
atom	22.40562087	0.44228235	9.32986158	H
atom	12.02642419	2.37951532	1.09020009	H
atom	29.41760154	1.53724316	7.77141104	H
atom	5.36703862	5.45907094	5.51817851	H
atom	22.75915505	6.29496286	3.34250921	H
atom	12.39074022	0.64336732	1.12006976	H

atom	29.78223490	3.27339106	7.74236561	H
atom	5.00289052	7.19527579	5.54770632	H
atom	22.39397469	4.55893293	3.31363637	H
atom	10.74023306	1.18494249	0.75765691	H
atom	28.13157382	2.73203594	8.10402382	H
atom	6.65341624	6.65352355	5.18574507	H
atom	24.04469898	5.09975628	3.67578284	H
atom	14.03237472	7.34786188	7.93301164	H
atom	31.42416151	4.40635094	0.92973298	H
atom	3.36038827	0.49029464	3.50370002	H
atom	20.75215229	3.42664757	5.35660795	H
atom	16.15416936	3.44524504	1.50450081	H
atom	33.54538175	0.46832854	7.35711567	H
atom	1.23893684	4.39207721	5.93068512	H
atom	18.63159948	7.36472898	2.92716649	H
atom	16.10015715	4.00611720	7.51919876	H
atom	33.49395584	7.74719958	1.34248999	H
atom	1.29327504	3.83180289	3.08935360	H
atom	18.68357694	0.08636125	5.76946010	H
atom	13.29690563	6.93638960	5.38492769	H
atom	30.68899950	4.81644716	3.47703297	H
atom	4.09629743	0.90150714	0.95608690	H
atom	21.48828725	3.01583086	7.90404425	H
atom	13.75378056	5.27257706	5.80801364	H
atom	31.14675353	6.47992359	3.05378030	H
atom	3.63874197	2.56516753	1.37871221	H
atom	21.03088408	1.35223771	7.48106338	H
atom	12.06684582	5.79746287	5.99304891	H
atom	29.45960193	5.95601886	2.86863413	H
atom	5.32574498	2.04088071	1.56461849	H
atom	22.71806457	1.87622061	7.29641476	H
atom	16.10351559	7.74827296	1.67581356	H
atom	33.49505170	4.00424759	7.18707865	H
atom	1.28964562	0.08909792	6.10276314	H
atom	18.68208419	3.82878756	2.75650996	H
atom	16.49846433	-0.26248967	7.85490960	H
atom	33.89166162	4.17690577	1.00814259	H
atom	0.89432197	8.10033966	3.42484235	H
atom	18.28475236	3.65687775	5.43388105	H
atom	13.65826802	1.63166786	7.78733951	C
atom	31.05116950	2.28369560	1.07487984	C
atom	3.73478536	6.20657205	3.35818470	C
atom	21.12579533	5.54922602	5.50216852	C
atom	12.17593820	1.43230026	7.99560032	C
atom	29.56896151	2.48372700	0.86640279	C
atom	5.21702993	6.40610540	3.56683922	C

atom	22.60802262	5.34879892	5.29417535	C
atom	13.91911652	5.51393192	3.04753462	C
atom	31.31172255	6.23900570	5.81427225	C
atom	3.47347649	2.32373665	7.47488638	C
atom	20.86592494	1.59380767	1.38487565	C
atom	14.50058846	6.74237559	2.70818693	C
atom	31.89272364	5.01041568	6.15393298	C
atom	2.89218071	1.09520995	7.13553500	C
atom	20.28484538	2.82245622	1.72416251	C
atom	14.52395735	4.33360497	2.60490607	C
atom	31.91688972	7.41920868	6.25676316	C
atom	2.86878862	3.50396989	7.03181124	C
atom	20.26053087	0.41366151	1.82724178	C
atom	14.25472393	2.89085294	7.66582878	C
atom	31.64708283	1.02419113	1.19613058	C
atom	3.13853869	4.94730448	3.23646862	C
atom	20.53014414	6.80886156	5.62347872	C
atom	12.70517133	5.41270468	3.94304959	C
atom	30.09791496	6.34059395	4.91865852	C
atom	4.68814078	2.42523535	-0.48573093	C
atom	22.07898258	1.49202636	9.34628177	C
atom	11.80328192	1.41613916	0.61535708	C
atom	29.19452405	2.50047783	8.24656040	C
atom	5.59039090	6.42235721	5.04324692	C
atom	22.98180322	5.33172217	3.81787967	C
atom	14.47853448	0.49832595	7.82945558	C
atom	31.87188364	3.41673416	1.03316780	C
atom	2.91436035	7.33979781	3.40004328	C
atom	20.30477236	4.41643495	5.45994325	C
atom	16.43310536	1.88342374	7.70601531	C
atom	33.82589418	2.03081963	1.15632665	C
atom	0.96000783	5.95444594	3.27590452	C
atom	18.35107108	5.80286579	5.58262433	C
atom	15.68670984	4.36709467	1.84024138	C
atom	33.07952404	7.38550464	7.02157565	C
atom	1.70633026	3.47030958	6.26673484	C
atom	19.09763374	0.44749652	2.59163702	C
atom	15.64140735	3.02403311	7.60622244	C
atom	33.03370470	0.89047213	1.25565750	C
atom	1.75190320	4.81393777	3.17640872	C
atom	19.14353289	6.94296406	5.68254688	C
atom	12.96341412	5.89061979	5.36686761	C
atom	30.35603968	5.86236806	3.49493323	C
atom	4.42951273	1.94734321	0.93802029	C
atom	21.82139531	1.96996289	7.92236323	C
atom	15.65644796	6.79188851	1.93362767	C

atom	33.04840971	4.96069073	6.92874367	C
atom	1.73661120	1.04553214	6.36056357	C
atom	19.12886852	2.87230717	2.49852881	C
atom	15.86313995	0.61689867	7.79550936	C
atom	33.25641718	3.29760340	1.06719842	C
atom	1.52978079	7.22103649	3.36559380	C
atom	18.92025428	4.53595712	5.49342470	C
atom	16.23980534	5.60024343	1.50546737	C
atom	33.63211788	6.15222831	7.35675085	C
atom	1.15337625	2.23709860	5.93196776	C
atom	18.54498475	1.68083623	2.92649055	C

Supplementary Table 33 | Input geometry.in file of [S-2-Me-BuA]$_2$PbBr$_4$ *experimental* structure

```
lattice_vector      15.6508998871       0.0000000000       0.0000000000
lattice_vector       0.0000000000       8.2846002579       0.0000000000
lattice_vector      -1.5775239343       0.0000000000       8.0757655110

atom    7.418079853       2.346198797       2.018133879 Pb
atom    6.655295849       6.488499165       6.057631969 Pb
atom    7.780080318       4.916910172       0.496659607 Br
atom    6.293295383       0.774610281       7.579105854 Br
atom    7.108809471       8.065887451       3.548491240 Br
atom    6.964566708       3.923586607       4.527274609 Br
atom    4.508862019       2.607163668       1.325233221 Br
atom    9.564513206       6.749464035       6.750532627 Br
atom    10.320226669      2.125828266       2.728801250 Br
atom    3.753148317       6.268128872       5.346963882 Br
atom    10.027739525      6.851364613       1.897804856 N
atom    4.045636654       2.709064484       6.177960396 N
atom    4.882758141       5.832358360       2.140885592 N
atom    9.190617561       1.690058470       5.934880257 N
atom    9.945204735       7.617110252       2.340833426 H
atom    4.128170967       3.474810123       5.734932423 H
atom    9.221228600       6.557037830       1.659392118 H
atom    4.852147579       2.414737225       6.416373253 H
atom    10.520330429      6.983056068       1.166326284 H
atom    3.553045273       2.840756178       6.909439087 H
atom    4.720666409       6.057782650       2.985820532 H
atom    9.352709770       1.915482402       5.089944839 H
atom    5.760635376       5.823941231       1.997855663 H
atom    8.312741280       1.681641340       6.077909946 H
atom    4.550125599       5.019747257       1.982988000 H
atom    9.523250580       0.877446949       6.092777252 H
atom    3.191000223       5.554965019       0.099186555 H
atom    10.882375717      1.412665129       7.976578712 H
atom    11.788506508      7.173353672       3.791265249 H
atom    2.284870148       3.031053543       4.284500122 H
atom    10.819477081      5.035926819       2.256401300 H
atom    3.253899097       0.893626630       5.819364071 H
atom    10.120545387      5.665009499       3.519313574 H
atom    3.952830315       1.522709608       4.556452274 H
atom    4.061411381       7.638823986       1.718022227 H
atom    10.011963844      3.496523857       6.357743263 H
atom    4.851793289       7.036044598       0.507174253 H
atom    9.221582413       2.893744469       7.568591118 H
atom    13.142057419      7.771336079       2.374081373 H
atom    0.931317568       3.629036188       5.701684475 H
```

atom	12.737717628	6.618558884	1.391632080	H
atom	1.335658193	2.476258755	6.684133053	H
atom	13.806630135	6.357867241	2.508348942	H
atom	0.266745895	2.215567350	5.567416668	H
atom	1.778174520	6.665474415	2.219688654	H
atom	12.295201302	2.523174524	5.856076717	H
atom	2.388965845	5.226605415	2.200944901	H
atom	11.684410095	1.084305048	5.874820709	H
atom	1.654438734	7.848142624	7.300087929	H
atom	12.418937683	3.705842495	0.775677323	H
atom	1.981193900	8.102927208	0.134396896	H
atom	12.092182159	3.960627079	7.941369057	H
atom	0.334157407	7.036864758	7.062862873	H
atom	13.739217758	2.894564629	1.012902856	H
atom	12.854879379	4.536945820	3.444903612	H
atom	1.218497157	0.394645244	4.630861759	H
atom	11.756744385	4.828753948	4.532095432	H
atom	2.316631556	0.686453760	3.543670416	H
atom	0.372311205	4.651745319	1.674332380	H
atom	13.701065063	0.509445012	6.401432991	H
atom	0.815555096	5.030235291	0.219038993	H
atom	13.257821083	0.887935042	7.856726646	H
atom	0.021961674	6.063838482	1.090510964	H
atom	14.051414490	1.921538591	6.985254765	H
atom	13.316196442	5.616163731	5.931746483	H
atom	0.757179022	1.473863602	2.144019127	H
atom	13.781694412	6.665209770	4.863847733	H
atom	0.291681498	2.522909164	3.211917877	H
atom	14.419481277	5.233307362	4.885894775	H
atom	-0.346105605	1.091007352	3.189870834	H
atom	2.971123457	6.346004009	0.637985528	C
atom	11.102252007	2.203703642	7.437780380	C
atom	11.977429390	6.428850174	3.181851625	C
atom	2.095945835	2.286549568	4.893914223	C
atom	10.669686317	5.857212543	2.745760441	C
atom	3.403689384	1.714912176	5.330005169	C
atom	4.233243465	6.818226337	1.227516294	C
atom	9.840132713	2.675925970	6.848248959	C
atom	12.996106148	6.826510429	2.293517590	C
atom	1.077269316	2.684210539	5.782248020	C
atom	1.978867412	5.906919956	1.647456169	C
atom	12.094509125	1.764619827	6.428309441	C
atom	0.898983061	7.439570904	7.728507996	C
atom	13.174391747	3.297271013	0.347257942	C
atom	12.484778404	5.219297886	4.029807091	C
atom	1.588597536	1.076997995	4.045958519	C

atom	0.679110706	5.360136509	1.106379867	C
atom	13.394265175	1.217836380	6.969386101	C
atom	13.621069908	5.732943535	5.023126125	C
atom	0.452305764	1.590643287	3.052639484	C

Supplementary Table 34 | Input geometry.in file of [S-2-Me-BuA]$_2$PbBr$_4$ *relaxed* structure

lattice_vector	15.38113645	-0.00000098	0.01624944	
lattice_vector	-0.00000081	8.19245362	0.00001693	
lattice_vector	-1.45316754	0.00001704	8.02970794	
atom	7.30120631	2.19810871	2.00814229	Pb
atom	6.62661503	6.29427417	6.03776402	Pb
atom	7.66084041	4.81226958	0.57344358	Br
atom	6.26704472	0.71597550	7.47254622	Br
atom	7.08160300	7.78038232	3.47743899	Br
atom	6.84632531	3.68406353	4.56855280	Br
atom	4.30791094	2.46827145	1.35272149	Br
atom	9.61990462	6.56439556	6.69318173	Br
atom	10.27282127	1.99088023	2.68381575	Br
atom	3.65498313	6.08704176	5.36208518	Br
atom	10.01744639	6.96641336	1.80081778	N
atom	3.91051795	2.87019335	6.24517683	N
atom	4.74920526	5.67248071	2.24813745	N
atom	9.17876171	1.57623787	5.79786908	N
atom	10.07752683	7.94981115	2.15844310	H
atom	3.85045133	3.85360350	5.88756083	H
atom	9.01873820	6.74637574	1.64805725	H
atom	4.90923093	2.65013963	6.39792539	H
atom	10.46990371	6.92912321	0.85596298	H
atom	3.45808566	2.83291138	7.19004350	H
atom	4.31401278	5.72069405	3.20010195	H
atom	9.61400060	1.62443389	4.84590979	H
atom	5.75581409	5.86565076	2.38033628	H
atom	8.17216045	1.76942447	5.66562950	H
atom	4.65000929	4.69227760	1.89235535	H
atom	9.27794744	0.59604073	6.15368290	H
atom	3.08907097	5.33667570	0.05792033	H
atom	10.83891972	1.24043884	7.98806670	H
atom	11.68978626	7.23692325	4.07315974	H
atom	2.23821716	3.14067629	3.97278640	H
atom	10.72341253	5.03054431	2.17636138	H
atom	3.20454314	0.93433719	5.86967301	H
atom	9.85523957	5.78256982	3.52594821	H
atom	4.07274375	1.68630989	4.52007886	H
atom	4.03457399	7.61370633	1.88616853	H
atom	9.89338057	3.51747462	6.15983174	H
atom	4.90921911	6.87456109	0.53455980	H
atom	9.01874743	2.77832000	7.51143253	H
atom	12.62431558	7.89351866	1.90668516	H
atom	1.30357381	3.79739809	6.13914606	H

atom	13.06324091	6.21540312	1.51529276	H
atom	0.86476820	2.11934525	6.53071900	H
atom	13.91341030	7.09548648	2.80055661	H
atom	0.01456354	2.99920569	5.24539797	H
atom	1.52613267	6.63398055	2.35507438	H
atom	12.40179448	2.53776556	5.69089551	H
atom	2.25614549	5.03741133	2.41429463	H
atom	11.67181507	0.94119329	5.63169683	H
atom	1.65855218	7.62057236	7.07616684	H
atom	12.26943069	3.52428124	0.96985824	H
atom	2.05465939	8.21760178	0.34778445	H
atom	11.87328982	4.12137331	7.69823352	H
atom	0.01383918	6.99611035	7.22411281	H
atom	13.91414892	2.89983149	0.82187592	H
atom	12.88886863	4.47777419	3.52536910	H
atom	1.03902178	0.38160964	4.52065483	H
atom	11.63315987	4.75558058	4.72446805	H
atom	2.29481737	0.65925723	3.32161824	H
atom	-0.10546707	4.75544613	1.89124934	H
atom	14.03344741	0.65926711	6.15467966	H
atom	0.84085007	4.29044272	0.46376816	H
atom	13.08718060	0.19425952	7.58217471	H
atom	-0.00958292	5.84476025	0.50223831	H
atom	13.93758523	1.74856613	7.54367388	H
atom	13.07615673	6.36323540	5.96172816	H
atom	0.85202464	2.26686057	2.08410517	H
atom	14.40117130	6.12021521	4.79881150	H
atom	-0.47308024	2.02417504	3.24703882	H
atom	13.86435550	4.78425258	5.82299735	H
atom	0.06360289	0.68797692	2.22301858	H
atom	2.85108093	6.21435932	0.68594350	C
atom	11.07689102	2.11812535	7.36005273	C
atom	11.92388881	6.41528835	3.37338865	C
atom	2.00408832	2.31907457	4.67258832	C
atom	10.60489533	5.96246727	2.74513103	C
atom	3.32307879	1.86624052	5.30087595	C
atom	4.16116633	6.68438434	1.31463808	C
atom	9.76680495	2.58815079	6.73135698	C
atom	12.93157436	6.93093523	2.33880059	C
atom	0.99638900	2.83477093	5.70714537	C
atom	1.80833693	5.77263934	1.72739350	C
atom	12.11962898	1.67642398	6.31858526	C
atom	0.88553155	7.32754305	7.79792250	C
atom	13.04244461	3.23126729	0.24808581	C
atom	12.47495659	5.24063407	4.20654328	C
atom	1.45302597	1.14439735	3.83946376	C

atom	0.56599085	5.13629682	1.11146322	C
atom	13.36200612	1.04009935	6.93447955	C
atom	13.51357648	5.65149834	5.24821657	C
atom	0.41449184	1.55526504	2.79770690	C

Supplementary Table 35 | Input geometry.in file of [S-1-1-NEA]$_2$PbBr$_4$ *experimental* structure

lattice_vector	19.3984513056	-1.2900429452	0.0000000000	
lattice_vector	0.0000000000	8.7239398956	0.0000000000	
lattice_vector	0.0000000000	0.0000000000	7.9297099113	
atom	9.641612053	1.510046244	0.295936763	Pb
atom	9.756838799	5.923850536	4.260791779	Pb
atom	12.553319931	1.652980328	0.329082936	Br
atom	6.845130920	5.780917168	4.293937683	Br
atom	6.730292320	1.139565945	7.533065796	Br
atom	12.668158531	6.294331074	3.568210840	Br
atom	9.955090523	3.384039164	5.833094597	Br
atom	9.443360329	4.049857616	1.868239641	Br
atom	9.896895409	7.383561134	6.883226395	Br
atom	9.501555443	0.050336108	2.918371201	Br
atom	12.413069725	5.002093315	0.352079123	N
atom	6.985381603	2.431803703	4.316934109	N
atom	12.457685471	0.614474058	4.868049145	N
atom	6.940765858	6.819422722	0.903194010	N
atom	11.635830879	5.282348633	0.020966154	H
atom	7.762619495	2.151548386	3.985821247	H
atom	12.524977684	5.335539341	1.169378519	H
atom	6.873472691	2.098357916	5.134232998	H
atom	12.415881157	4.113163471	0.390815735	H
atom	6.982569695	3.320733309	4.355670452	H
atom	11.653154373	0.851398170	4.567798138	H
atom	7.745296955	6.582499027	0.602943480	H
atom	12.541486740	0.865219116	5.717987061	H
atom	6.856964111	6.568677902	1.753131986	H
atom	12.547500610	-0.268595070	4.808750629	H
atom	6.850950241	7.702491760	0.843895674	H
atom	13.334262848	5.213638306	6.466440201	H
atom	6.064188480	2.220258713	2.501585484	H
atom	13.422060013	0.969273090	3.117462158	H
atom	5.976390839	6.464623928	7.082317352	H
atom	12.357297897	2.927244902	3.694483519	H
atom	7.041152954	4.506651878	7.659338474	H
atom	13.887176514	3.224275589	3.524605513	H
atom	5.511274338	4.209621429	7.489460468	H
atom	13.269335747	3.089986563	4.959121704	H
atom	6.129114628	4.343910217	0.994266570	H
atom	12.689961433	7.327785015	7.282922745	H
atom	6.708489418	0.106112249	3.318068266	H
atom	14.199840546	7.329068661	6.861213207	H
atom	5.198610306	0.104827777	2.896358252	H

atom	13.809408188	7.239424229	0.447124630	H
atom	5.589043140	0.194472909	4.411979675	H
atom	14.659137726	1.695068002	6.339025974	H
atom	4.739313602	5.738829136	2.374171019	H
atom	14.406143188	4.341986656	1.724664330	H
atom	4.992307663	3.091910362	5.689519405	H
atom	14.697604179	0.084051155	1.971944571	H
atom	4.700846672	7.349845886	5.936799526	H
atom	14.955508232	5.708095551	5.254931450	H
atom	4.442943096	1.725800872	1.290076613	H
atom	16.693937302	5.638426304	3.767666817	H
atom	2.704513073	1.795470357	7.732522011	H
atom	18.387210846	7.500414371	1.413336158	H
atom	1.011240959	-0.066517107	5.378190994	H
atom	16.451961517	3.457935095	2.306641817	H
atom	2.946489334	3.975961685	6.271496773	H
atom	18.174970627	3.469150066	0.855520546	H
atom	1.223479986	3.964746714	4.820375443	H
atom	16.797817230	1.268065333	7.116470814	H
atom	2.600634336	6.165831566	3.151615620	H
atom	19.007299423	-0.554931343	3.482546091	H
atom	0.391150624	7.988827705	7.447401047	H
atom	18.364183426	0.361758202	5.726788521	H
atom	1.034267426	7.072138786	1.761933804	H
atom	16.269054413	-0.912991583	0.667776763	H
atom	3.129396915	8.346887589	4.632631779	H
atom	19.059404373	3.981951714	6.518617630	H
atom	0.339045912	3.451945305	2.553763151	H
atom	18.768970490	4.810560226	4.417268276	H
atom	0.629480124	2.623336792	0.452413738	H
atom	13.536238670	5.469156742	7.390489578	C
atom	5.862212181	1.964740634	3.425634623	C
atom	13.518780708	1.283698797	4.040187359	C
atom	5.879670143	6.150197506	0.075332217	C
atom	13.231683731	2.766668558	4.060011387	C
atom	6.166767597	4.667228222	0.095156476	C
atom	14.892190933	0.941114247	4.499317169	C
atom	4.506259918	6.492782593	0.534462512	C
atom	14.861152649	4.851503372	7.793318748	C
atom	4.537297726	2.582393646	3.828464031	C
atom	13.561457634	6.979338169	7.485646248	C
atom	5.836993694	0.454558641	3.520791292	C
atom	15.916429520	4.845009804	6.836996078	C
atom	3.482021570	2.588887215	2.872141123	C
atom	17.171508789	0.131749138	4.083800316	C
atom	2.226941586	7.302147388	0.118945532	C

atom	15.844655037	0.331654549	3.619912624	C
atom	3.553796291	7.102242470	7.584767342	C
atom	15.276280403	1.289828420	5.774414539	C
atom	4.122170448	6.144068718	1.809559703	C
atom	15.093935013	4.340503693	1.098264813	C
atom	4.304515839	3.093393564	5.063119411	C
atom	15.551737785	-0.057146166	2.311510324	C
atom	3.846712828	7.491043091	6.276365280	C
atom	15.770940781	5.350205421	5.520664215	C
atom	3.627509832	2.083691835	1.555809140	C
atom	17.192848206	4.337014198	7.216035843	C
atom	2.205603361	3.096882820	3.251181126	C
atom	16.814577103	5.320057869	4.630950451	C
atom	2.583873510	2.113838673	0.666095555	C
atom	17.761220932	-0.823481798	1.974497914	C
atom	1.637229204	8.257378578	5.939352989	C
atom	16.333496094	3.814893484	1.459066629	C
atom	3.064954996	3.619003773	5.423921585	C
atom	17.351915359	3.818702221	0.594728291	C
atom	2.046536207	3.615194559	4.559583187	C
atom	16.570156097	1.052858353	6.240681648	C
atom	2.828294039	6.381038666	2.275826693	C
atom	18.127851486	-0.446562380	3.203602791	C
atom	1.270598888	7.880459309	7.168457508	C
atom	17.499343872	0.502524674	5.415991783	C
atom	1.899107814	6.931372643	1.451136947	C
atom	16.496442795	-0.634683728	1.530434132	C
atom	2.902008772	8.068580627	5.495289326	C
atom	18.230665207	4.327319622	6.272400379	C
atom	1.167786241	3.106577635	2.307545662	C
atom	18.059957504	4.818488121	5.019506454	C
atom	1.338493228	2.615408421	1.054651260	C

Supplementary Table 36 | Input geometry.in file of [S-1-1-NEA]$_2$PbBr$_4$ *relaxed* structure

lattice_vector	-19.22478634	1.30473266	-0.00799337	
lattice_vector	-0.22263015	8.76022728	-0.05995493	
lattice_vector	-0.00001155	-0.05336867	-7.78810274	
atom	9.50593812	1.50003325	0.36546718	Pb
atom	9.49653022	5.98829082	4.22849770	Pb
atom	12.47230089	1.70258061	0.36940363	Br
atom	6.53016465	5.78608346	4.23592853	Br
atom	6.58963852	1.10624420	7.39071586	Br
atom	12.41264112	6.42557760	3.45928448	Br
atom	9.81905105	3.43526691	5.74185315	Br
atom	9.18340664	4.07386910	1.84319306	Br
atom	9.59941380	7.33296797	6.94308129	Br
atom	9.40260747	0.19309142	3.09795210	Br
atom	12.22331687	5.03200861	0.34333408	N
atom	6.77895308	2.45658563	4.25592292	N
atom	11.30093523	5.34445073	-0.00988284	H
atom	7.70128140	2.13908767	3.90708419	H
atom	12.30137611	5.40913843	1.31505001	H
atom	6.70082186	2.09290384	5.23275105	H
atom	12.21558358	3.98408315	0.38328051	H
atom	6.78686365	3.50495152	4.28141400	H
atom	12.42260609	0.68726629	4.81846245	N
atom	6.57986929	6.80924040	0.88264800	N
atom	11.47885961	0.91314068	4.44734249	H
atom	7.52362101	6.57843367	0.51459224	H
atom	12.43646584	0.92632956	5.83260533	H
atom	6.56603435	6.58401363	1.89994597	H
atom	12.53851040	-0.34147674	4.67411702	H
atom	6.46383867	7.83591682	0.72429009	H
atom	13.31848564	5.59312937	7.23176407	C
atom	5.68373058	1.93669952	3.36328652	C
atom	13.01671771	5.27536003	6.22335128	H
atom	5.98553029	2.24063640	2.35062753	H
atom	13.48955528	1.42992922	4.04093780	C
atom	5.51298806	6.05595191	0.11532695	C
atom	13.32488176	1.12091105	3.00387203	H
atom	5.67764392	6.40418578	6.86224350	H
atom	13.22114294	2.92265567	4.14779348	C
atom	5.78149925	4.56484014	0.24255557	C
atom	12.26827173	3.19018311	3.67630208	H
atom	6.73435749	4.34431109	7.56278830	H
atom	14.02746998	3.47193342	3.65602370	H
atom	4.97515777	4.06222253	7.54652910	H

atom	13.19179794	3.25033771	5.19344461	H
atom	5.81097289	4.25148767	1.29258907	H
atom	14.88713693	1.04265465	4.49451704	C
atom	4.11539615	6.44927394	0.56362637	C
atom	14.66692203	4.98541123	7.57150000	C
atom	4.33533790	2.54910175	3.69466070	C
atom	13.31146772	7.11591642	7.30421951	C
atom	5.69060639	0.41504449	3.45654897	C
atom	12.32768514	7.50418629	7.01889348	H
atom	6.67434443	0.02281268	3.17654266	H
atom	14.07304690	7.53753865	6.64067878	H
atom	4.92898602	-0.01555436	2.79884065	H
atom	13.51702978	7.39282846	0.53914908	H
atom	5.48501775	0.09880193	4.48399755	H
atom	15.69822610	4.99516638	6.57671527	C
atom	3.30407913	2.52581261	2.70005496	C
atom	17.16418731	0.20163116	4.03136896	C
atom	1.83836016	7.28407596	0.08931857	C
atom	15.80992015	0.41885423	3.59177915	C
atom	3.19254958	7.11409621	7.44065826	C
atom	15.30962006	1.37421989	5.77066347	C
atom	3.69300984	6.13506072	1.84419058	C
atom	14.62287814	1.85230515	6.47027908	H
atom	4.37980470	5.66649962	2.55016817	H
atom	14.93652668	4.39468229	1.02643953	C
atom	4.06568291	3.10339059	4.93023298	C
atom	14.17240825	4.39025741	1.80065729	H
atom	4.82977645	3.11838002	5.70434423	H
atom	15.46827344	0.00163517	2.27824309	C
atom	3.53402688	7.51319260	6.12145556	C
atom	14.45766999	0.14904141	1.90569908	H
atom	4.54453837	7.36051298	5.75079074	H
atom	15.51367497	5.52238408	5.27281420	C
atom	3.48870243	1.98086666	1.40347870	C
atom	14.56170805	5.95995543	4.97750782	H
atom	4.44068721	1.53931974	1.11421503	H
atom	16.98174134	4.44103212	6.90226982	C
atom	2.02055080	3.08433822	3.01794456	C
atom	16.52076586	5.47868784	4.33483330	C
atom	2.48166478	2.01174579	0.46493423	C
atom	16.34443092	5.89377385	3.34521933	H
atom	2.65804863	1.63653614	7.26919298	H
atom	17.72906488	-0.75993503	1.87209216	C
atom	1.27327004	8.26945386	5.70528467	C
atom	18.46443617	-1.18505995	1.19396897	H
atom	0.53785392	8.68536895	5.02152443	H

atom	16.19905009	3.84510278	1.34114748	C
atom	2.80314559	3.65723306	5.23734144	C
atom	16.36151536	3.40970731	2.32624144	H
atom	2.64064912	4.10605463	6.21638574	H
atom	17.20842975	3.85889282	0.40916957	C
atom	1.79379663	3.63073344	4.30560414	C
atom	18.19262358	3.45154459	0.64139410	H
atom	0.80959948	4.04122116	4.53221982	H
atom	16.62460799	1.11596913	6.20665378	C
atom	2.37808214	6.39929701	2.27676236	C
atom	16.90410319	1.38596125	7.22209735	H
atom	2.09866879	6.14312635	3.29580212	H
atom	18.10064142	-0.37282786	3.13711228	C
atom	0.90184719	7.89975210	6.97554315	C
atom	19.13246427	-0.49383920	3.46298149	H
atom	-0.12989882	8.02535523	7.29991102	H
atom	17.54084733	0.55868015	5.34852084	C
atom	1.46181570	6.94502718	1.41125636	C
atom	18.56967211	0.38583866	5.66682407	H
atom	0.43304802	7.12230969	1.72729226	H
atom	16.39712249	-0.58884033	1.44872904	C
atom	2.60510660	8.09234339	5.28406867	C
atom	16.10720905	-0.91688469	0.45332341	H
atom	2.89487993	8.40662478	4.28419311	H
atom	17.99127130	4.39722299	5.90867968	C
atom	1.01107388	3.11458592	2.02379430	C
atom	18.95575892	3.96411666	6.17522146	H
atom	0.04657407	3.55128897	2.28433982	H
atom	17.76937198	4.90200716	4.64762409	C
atom	1.23303592	2.59263159	0.76975868	C
atom	18.55675819	4.87026043	3.89410491	H
atom	0.44569902	2.61408678	0.01582451	H

Supplementary Table 37 | Input geometry.in file of [4AMP]$PbBr_4$ *experimental* structure

lattice_vector	0.0000000000	-10.2349996567	0.0000000000	
lattice_vector	17.7450008392	0.0000000000	0.0000000000	
lattice_vector	0.0000000000	0.0000000000	7.8298997879	
atom	2.176601887	5.192112923	1.264763713	Pb
atom	15.568399429	5.042886734	5.179713726	Pb
atom	11.049101830	5.042886734	1.264763713	Pb
atom	6.695898533	5.192112923	5.179713726	Pb
atom	2.699192047	2.117519140	1.106364727	Br
atom	15.045808792	8.117480278	5.021314621	Br
atom	11.571692467	8.117480278	1.106364727	Br
atom	6.173308849	2.117519140	5.021314621	Br
atom	2.005362511	8.041639328	1.455578446	Br
atom	15.739638329	2.193360090	5.370528221	Br
atom	10.877862930	2.193360090	1.455578446	Br
atom	6.867137909	8.041639328	5.370528221	Br
atom	4.134407520	5.317798615	3.632290602	Br
atom	13.610592842	4.917201042	7.547240257	Br
atom	13.006908417	4.917201042	3.632290602	Br
atom	4.738092899	5.317798615	7.547240257	Br
atom	17.085950851	5.089865208	2.626931429	Br
atom	0.659049332	5.145133972	6.541881084	Br
atom	8.213450432	5.145133972	2.626931429	Br
atom	9.531550407	5.089865208	6.541881084	Br
atom	2.090361118	7.811351776	4.846708298	N
atom	15.654640198	2.423648119	0.931757748	N
atom	10.962861061	2.423648119	4.846708298	N
atom	6.782139301	7.811351776	0.931757748	N
atom	2.965189695	2.689757586	5.650838852	N
atom	14.779810905	7.545241833	1.735888362	N
atom	11.837690353	7.545241833	5.650838852	N
atom	5.907310486	2.689757586	1.735888362	N
atom	2.290879488	7.816469193	3.898507118	H
atom	15.454121590	2.418530226	7.813457012	H
atom	11.163379669	2.418530226	3.898507118	H
atom	6.581620693	7.816469193	7.813457012	H
atom	2.037126064	6.887131691	5.137197018	H
atom	15.707875252	3.347868204	1.222247601	H
atom	10.909626007	3.347868204	5.137197018	H
atom	6.835374355	6.887131691	1.222247601	H
atom	3.781459808	2.461517096	5.376791954	H
atom	13.963541031	7.773482323	1.461842179	H
atom	12.653960228	7.773482323	5.376791954	H
atom	5.091040611	2.461517096	1.461842179	H

atom	2.895984173	2.548514366	6.526221752	H
atom	14.849016190	7.686485291	2.611271858	H
atom	11.768485069	7.686485291	6.526221752	H
atom	5.976515770	2.548514366	2.611271858	H
atom	2.825004101	3.551544905	5.477797508	H
atom	14.919996262	6.683454514	1.562847853	H
atom	11.697504997	6.683454514	5.477797508	H
atom	6.047495842	3.551544905	1.562847853	H
atom	3.971331358	0.166830122	5.647706985	H
atom	13.773669243	10.068169594	1.732756853	H
atom	12.843832016	10.068169594	5.647706985	H
atom	4.901169300	0.166830122	1.732756853	H
atom	3.488667250	10.005735397	4.207787991	H
atom	14.256333351	0.229264215	0.292838305	H
atom	12.361167908	0.229264215	4.207787991	H
atom	5.383832932	10.005735397	0.292838305	H
atom	1.754980564	0.422706097	6.354746819	H
atom	15.990019798	9.812294006	2.439796448	H
atom	10.627481461	9.812294006	6.354746819	H
atom	7.117519855	0.422706097	2.439796448	H
atom	0.548320532	8.408052444	6.014928818	H
atom	17.196680069	1.826947451	2.099978447	H
atom	9.420821190	1.826947451	6.014928818	H
atom	8.324179649	8.408052444	2.099978447	H
atom	0.099372000	8.025262833	4.559350491	H
atom	17.645629883	2.209736347	0.644400358	H
atom	8.971872330	2.209736347	4.559350491	H
atom	8.773128510	8.025262833	0.644400358	H
atom	2.140047073	1.898592710	3.983070135	H
atom	15.604953766	8.336406708	0.068120234	H
atom	11.012547493	8.336406708	3.983070135	H
atom	6.732453346	1.898592710	0.068120234	H
atom	1.075347066	2.255793571	5.078473091	H
atom	16.669654846	7.979206085	1.163522959	H
atom	9.947847366	7.979206085	5.078473091	H
atom	7.797152996	2.255793571	1.163522959	H
atom	4.026340485	8.048804283	5.400281906	H
atom	13.718660355	2.186195612	1.485332012	H
atom	12.898840904	2.186195612	5.400281906	H
atom	4.846159935	8.048804283	1.485332012	H
atom	3.014875650	8.441827774	6.537183285	H
atom	14.730125427	1.793171763	2.622233629	H
atom	11.887375832	1.793171763	6.537183285	H
atom	5.857625008	8.441827774	2.622233629	H
atom	1.022112012	9.982194901	3.716070414	H
atom	16.722888947	0.252804339	7.631020069	H

atom	9.894613266	0.252804339	3.716070414	H
atom	7.850388527	9.982194901	7.631020069	H
atom	0.008872501	0.116678834	4.854537964	H
atom	17.736127853	10.118320465	0.939588010	H
atom	8.881373405	10.118320465	4.854537964	H
atom	8.863628387	0.116678834	0.939588010	H
atom	3.265080214	9.958654404	5.152073860	C
atom	14.479920387	0.276345104	1.237124085	C
atom	12.137580872	0.276345104	5.152073860	C
atom	5.607419968	9.958654404	1.237124085	C
atom	1.948401213	0.441128492	5.394801140	C
atom	15.796600342	9.793870926	1.479851127	C
atom	10.820900917	9.793870926	5.394801140	C
atom	6.924098969	0.441128492	1.479851127	C
atom	0.786103547	8.472532272	5.073775291	C
atom	16.958896637	1.762467146	1.158825159	C
atom	9.658604622	1.762467146	5.073775291	C
atom	8.086397171	8.472532272	1.158825159	C
atom	1.955499172	1.870958447	4.932837009	C
atom	15.789502144	8.364041328	1.017886877	C
atom	10.827999115	8.364041328	4.932837009	C
atom	6.917001724	1.870958447	1.017886877	C
atom	3.181678534	8.491979599	5.582718372	C
atom	14.563322067	1.743020535	1.667769074	C
atom	12.054179192	1.743020535	5.582718372	C
atom	5.690821648	8.491979599	1.667769074	C
atom	0.862407029	9.927949905	4.674450397	C
atom	16.882593155	0.307049692	0.759500325	C
atom	9.734908104	0.307049692	4.674450397	C
atom	8.010093689	9.927949905	0.759500325	C

Supplementary Table 38 | Input geometry.in file of [4AMP]$PbBr_4$ *relaxed* structure

lattice_vector	0.00002408	10.40421661	-0.00000050	
lattice_vector	17.86913709	0.00000987	0.00000480	
lattice_vector	0.00005356	-0.00000112	7.69700138	
atom	2.18851307	5.27147499	1.24812003	Pb
atom	15.68094935	5.13225687	5.09703283	Pb
atom	11.12286815	5.13209523	1.24844512	Pb
atom	6.74651865	5.27157940	5.09713172	Pb
atom	2.72479633	2.15958309	1.10850786	Br
atom	15.14442065	8.24452439	4.95698359	Br
atom	11.65935360	8.24455946	1.10853594	Br
atom	6.20976690	2.15966920	4.95698647	Br
atom	2.01638992	8.18429072	1.42822054	Br
atom	15.85282647	2.22026205	5.27665740	Br
atom	10.95099645	2.22042183	1.42820904	Br
atom	6.91832171	8.18427236	5.27658776	Br
atom	4.10891710	5.39690151	3.62714214	Br
atom	13.76053481	5.00742984	7.47559864	Br
atom	13.04341393	5.00737573	3.62709019	Br
atom	4.82597936	5.39684922	7.47554460	Br
atom	17.15455515	5.16460596	2.47626169	Br
atom	0.71477029	5.23967781	6.32476058	Br
atom	8.22009061	5.23971866	2.47616183	Br
atom	9.64926139	5.16458748	6.32465846	Br
atom	2.05609854	7.97244150	4.78916850	N
atom	15.81308484	2.43175413	0.94060668	N
atom	10.99067159	2.43176666	4.78915587	N
atom	6.87850978	7.97247931	0.94056956	N
atom	3.02783615	2.72670165	5.55354439	N
atom	14.84131870	7.67751378	1.70500171	N
atom	11.96240019	7.67752128	5.55357107	N
atom	5.90675978	2.72672871	1.70499797	N
atom	2.21928057	7.92155126	3.75516169	H
atom	15.64992757	2.48262566	7.60361031	H
atom	11.15386328	2.48265058	3.75514810	H
atom	6.71533094	7.92160357	7.60357697	H
atom	1.96365279	6.99463069	5.13325390	H
atom	15.90554921	3.40956245	1.28468017	H
atom	10.89823501	3.40957329	5.13324180	H
atom	6.97096462	6.99467397	1.28464129	H
atom	4.01584472	2.46311295	5.31368499	H
atom	13.85332222	7.94113041	1.46510879	H
atom	12.95040994	7.94110284	5.31369741	H
atom	4.91876179	2.46314141	1.46507247	H

atom	2.92484980	2.63393913	6.59294901	H
atom	14.94430073	7.77025846	2.74441565	H
atom	11.85942248	7.77031251	6.59297394	H
atom	6.00970699	2.63395540	2.74441351	H
atom	2.93683612	3.72724370	5.30650322	H
atom	14.93235893	6.67696467	1.45799794	H
atom	11.87138242	6.67697290	5.30655951	H
atom	5.99783922	3.72728281	1.45803213	H
atom	4.15764332	0.14253548	5.55710602	H
atom	13.71154932	10.26169831	1.70862335	H
atom	13.09222360	10.26167726	5.55713267	H
atom	4.77697199	0.14255487	1.70863186	H
atom	3.54824732	10.16120220	3.95331792	H
atom	14.32090481	0.24300319	0.10482492	H
atom	12.48279947	0.24299916	3.95333701	H
atom	5.38634503	10.16125738	0.10482192	H
atom	1.80555078	0.43839463	6.38930637	H
atom	16.06364398	9.96580497	2.54080764	H
atom	10.74011533	9.96583309	6.38930807	H
atom	7.12908616	0.43842420	2.54077982	H
atom	0.52206730	8.56263592	6.08950665	H
atom	17.34712697	1.84159001	2.24095923	H
atom	9.45662280	1.84158057	6.08948514	H
atom	8.41259213	8.56263751	2.24088246	H
atom	-0.01045195	8.12929017	4.45367173	H
atom	17.87963127	2.27490833	0.60511983	H
atom	8.92412188	2.27492795	4.45364714	H
atom	8.94504517	8.12932678	0.60502856	H
atom	2.22111838	1.98395917	3.76105159	H
atom	15.64805318	8.42026192	-0.08746411	H
atom	11.15569688	8.42024419	3.76106970	H
atom	6.71348805	1.98399199	-0.08747234	H
atom	1.03141764	2.37438445	5.02739036	H
atom	16.83773575	8.02979718	1.17888945	H
atom	9.96598748	8.02982719	5.02740281	H
atom	7.90317833	2.37443759	1.17888316	H
atom	4.11919114	8.08755558	5.19586745	H
atom	13.74999645	2.31664538	1.34736890	H
atom	13.05375928	2.31664491	5.19587467	H
atom	4.81543354	8.08760273	1.34735239	H
atom	3.04975831	8.55314266	6.54172754	H
atom	14.81949277	1.85108789	2.69320508	H
atom	11.98431144	1.85107562	6.54173412	H
atom	5.88494805	8.55314795	2.69318179	H
atom	0.97170246	10.18637850	3.50895698	H
atom	16.89753269	0.21776929	7.35745617	H

atom	9.90626924	0.21783396	3.50894329	H
atom	7.96295272	10.18647918	7.35740304	H
atom	-0.12610319	0.20318203	4.83178514	H
atom	17.99528445	10.20100762	0.98331047	H
atom	8.80848452	10.20104292	4.83175511	H
atom	9.06069196	0.20322380	0.98323833	H
atom	3.30395827	10.10198947	5.02750600	C
atom	14.56522704	0.30222465	1.17900910	C
atom	12.23849949	0.30222144	5.02752173	C
atom	5.63068363	10.10202360	1.17900150	C
atom	1.99184197	0.45220810	5.30021943	C
atom	15.87734158	9.95199669	1.45172044	C
atom	10.92642265	9.95200826	5.30022440	C
atom	6.94276229	0.45224319	1.45169688	C
atom	0.75211664	8.67181560	5.02139298	C
atom	17.11707489	1.73238280	1.17284586	C
atom	9.68668116	1.73239948	5.02137297	C
atom	8.18250912	8.67185362	1.17277722	C
atom	2.00637637	1.90621845	4.83660446	C
atom	15.86279219	8.49799153	0.98809399	C
atom	10.94094721	8.49799555	4.83661989	C
atom	6.92822808	1.90625250	0.98808549	C
atom	3.21436742	8.64637713	5.45868230	C
atom	14.65483827	1.75783842	1.61016500	C
atom	12.14892612	1.75783472	5.45868960	C
atom	5.72028150	8.64640581	1.61014519	C
atom	0.83535915	10.12750896	4.60126665	C
atom	17.03382369	0.27667712	0.75276372	C
atom	9.76990888	0.27670590	4.60125036	C
atom	8.09926863	10.12756181	0.75270681	C

Supplementary Table 39 | Input geometry.in file of $[NMA]_2PbBr_4$ *experimental* structure

lattice_vector	41.3314018250	0.0000000000	0.0000000000
lattice_vector	0.0000000000	8.0801000595	0.0000000000
lattice_vector	0.0000000000	0.0000000000	8.0836000443

atom	20.665700912	6.165681839	2.375955820	Pb
atom	20.665700912	1.914418221	6.417756081	Pb
atom	0.000000000	2.125631809	2.375955820	Pb
atom	0.000000000	5.954468250	6.417756081	Pb
atom	20.665700912	3.551446199	3.913189888	Br
atom	20.665700912	4.528653622	7.954989910	Br
atom	0.000000000	7.591496468	3.913189888	Br
atom	0.000000000	0.488603771	7.954989910	Br
atom	17.658262253	5.988162041	2.493790627	Br
atom	23.673141479	2.091937780	6.535590649	Br
atom	17.658262253	2.091937780	6.535590649	Br
atom	23.673141479	5.988162041	2.493790627	Br
atom	38.323963165	1.948112726	2.493790627	Br
atom	3.007438421	6.131988049	6.535590649	Br
atom	38.323963165	6.131988049	6.535590649	Br
atom	3.007438421	1.948112726	2.493790627	Br
atom	20.665700912	7.400240421	5.074560642	Br
atom	20.665700912	0.679859638	1.032760262	Br
atom	0.000000000	3.360189915	5.074560642	Br
atom	0.000000000	4.719909668	1.032760262	Br
atom	15.765863419	6.030986786	6.331883907	C
atom	25.565538406	2.049113274	2.290083170	C
atom	15.765863419	2.049113274	2.290083170	C
atom	25.565538406	6.030986786	6.331883907	C
atom	36.431564331	1.990936637	6.331883907	C
atom	4.899837971	6.089163303	2.290083170	C
atom	36.431564331	6.089163303	2.290083170	C
atom	4.899837971	1.990936637	6.331883907	C
atom	15.170277596	6.783244133	5.376402378	C
atom	26.161123276	1.296855927	1.334602237	C
atom	15.170277596	1.296855927	1.334602237	C
atom	26.161123276	6.783244133	5.376402378	C
atom	35.835979462	2.743193626	5.376402378	C
atom	5.495422840	5.336905956	1.334602237	C
atom	35.835979462	5.336905956	1.334602237	C
atom	5.495422840	2.743193626	5.376402378	C
atom	13.846019745	6.598209858	4.984347820	C
atom	27.485380173	1.481890202	0.942548037	C
atom	13.846019745	1.481890202	0.942548037	C
atom	27.485380173	6.598209858	4.984347820	C

atom	34.511722565	2.558160305	4.984347820	C
atom	6.819681168	5.521940231	0.942548037	C
atom	34.511722565	5.521940231	0.942548037	C
atom	6.819681168	2.558160305	4.984347820	C
atom	13.705492973	4.814931393	6.560649872	C
atom	27.625907898	3.265168428	2.518849611	C
atom	13.705492973	3.265168428	2.518849611	C
atom	27.625907898	4.814931393	6.560649872	C
atom	34.371192932	0.774882078	6.560649872	C
atom	6.960207462	7.305218697	2.518849611	C
atom	34.371192932	7.305218697	2.518849611	C
atom	6.960207462	0.774882078	6.560649872	C
atom	13.089655876	5.580116749	5.604360104	C
atom	28.241746902	2.499983072	1.562559962	C
atom	13.089655876	2.499983072	1.562559962	C
atom	28.241746902	5.580116749	5.604360104	C
atom	33.755355835	1.540066361	5.604360104	C
atom	7.576045513	6.540033340	1.562559962	C
atom	33.755355835	6.540033340	1.562559962	C
atom	7.576045513	1.540066361	5.604360104	C
atom	11.746384621	5.363570213	5.182395935	C
atom	29.585016251	2.716529608	1.140595436	C
atom	11.746384621	2.716529608	1.140595436	C
atom	29.585016251	5.363570213	5.182395935	C
atom	32.412086487	1.323520422	5.182395935	C
atom	8.919316292	6.756579876	1.140595436	C
atom	32.412086487	6.756579876	1.140595436	C
atom	8.919316292	1.323520422	5.182395935	C
atom	13.217782021	7.383595467	3.994915009	C
atom	28.113618851	0.696504593	8.036715508	C
atom	13.217782021	0.696504593	8.036715508	C
atom	28.113618851	7.383595467	3.994915009	C
atom	33.883483887	3.343545437	3.994915009	C
atom	7.447918892	4.736554623	8.036715508	C
atom	33.883483887	4.736554623	8.036715508	C
atom	7.447918892	3.343545437	3.994915009	C
atom	11.936509132	7.221185684	3.636811733	C
atom	29.394893646	0.858914614	7.678611755	C
atom	11.936509132	0.858914614	7.678611755	C
atom	29.394893646	7.221185684	3.636811733	C
atom	32.602210999	3.181135416	3.636811733	C
atom	8.729191780	4.898964405	7.678611755	C
atom	32.602210999	4.898964405	7.678611755	C
atom	8.729191780	3.181135416	3.636811733	C
atom	11.188410759	6.165924549	4.228531361	C
atom	30.142992020	1.914175510	0.186731309	C

atom	11.188410759	1.914175510	0.186731309	C
atom	30.142992020	6.165924549	4.228531361	C
atom	31.854110718	2.125874519	4.228531361	C
atom	9.477290154	5.954225540	0.186731309	C
atom	31.854110718	5.954225540	0.186731309	C
atom	9.477290154	2.125874519	4.228531361	C
atom	17.183530807	6.216021061	6.673820496	C
atom	24.147871017	1.864079237	2.632020473	C
atom	17.183530807	1.864079237	2.632020473	C
atom	24.147871017	6.216021061	6.673820496	C
atom	37.849231720	2.175970793	6.673820496	C
atom	3.482170820	5.904129028	2.632020473	C
atom	37.849231720	5.904129028	2.632020473	C
atom	3.482170820	2.175970793	6.673820496	C
atom	15.005366325	4.917072296	6.931364059	C
atom	26.326036453	3.163027763	2.889563799	C
atom	15.005366325	3.163027763	2.889563799	C
atom	26.326036453	4.917072296	6.931364059	C
atom	35.671066284	0.877022207	6.931364059	C
atom	5.660335064	7.203077793	2.889563799	C
atom	35.671066284	7.203077793	2.889563799	C
atom	5.660335064	0.877022207	6.931364059	C
atom	15.667080879	7.452276230	4.963330746	H
atom	25.664319992	0.627823949	0.921531081	H
atom	15.667080879	0.627823949	0.921531081	H
atom	25.664319992	7.452276230	4.963330746	H
atom	36.332782745	3.412226200	4.963330746	H
atom	4.998619556	4.667873859	0.921531081	H
atom	36.332782745	4.667873859	0.921531081	H
atom	4.998619556	3.412226200	4.963330746	H
atom	13.188850403	4.172563553	6.991505623	H
atom	28.142551422	3.907536507	2.949705601	H
atom	13.188850403	3.907536507	2.949705601	H
atom	28.142551422	4.172563553	6.991505623	H
atom	33.854553223	0.132513478	6.991505623	H
atom	7.476850986	7.947586536	2.949705601	H
atom	33.854553223	7.947586536	2.949705601	H
atom	7.476850986	0.132513478	6.991505623	H
atom	11.246274948	4.674337864	5.556666374	H
atom	30.085128784	3.405762434	1.514866471	H
atom	11.246274948	3.405762434	1.514866471	H
atom	30.085128784	4.674337864	5.556666374	H
atom	31.911973953	0.634288132	5.556666374	H
atom	9.419425964	7.445812225	1.514866471	H
atom	31.911973953	7.445812225	1.514866471	H
atom	9.419425964	0.634288132	5.556666374	H

atom	13.717892647	8.045355797	3.571334600	H
atom	27.613510132	0.034744415	7.613134384	H
atom	13.717892647	0.034744415	7.613134384	H
atom	27.613510132	8.045355797	3.571334600	H
atom	34.383594513	4.005305767	3.571334600	H
atom	6.947808743	4.074794292	7.613134384	H
atom	34.383594513	4.074794292	7.613134384	H
atom	6.947808743	4.005305767	3.571334600	H
atom	11.543860435	7.787600517	3.012757778	H
atom	29.787542343	0.292499512	7.054557800	H
atom	11.543860435	0.292499512	7.054557800	H
atom	29.787542343	7.787600517	3.012757778	H
atom	32.209560394	3.747550011	3.012757778	H
atom	9.121840477	4.332549572	7.054557800	H
atom	32.209560394	4.332549572	7.054557800	H
atom	9.121840477	3.747550011	3.012757778	H
atom	10.312185287	6.020482540	3.962580919	H
atom	31.019214630	2.059617281	8.004381180	H
atom	10.312185287	2.059617281	8.004381180	H
atom	31.019214630	6.020482540	3.962580919	H
atom	30.977888107	1.980432749	3.962580919	H
atom	10.353515625	6.099667549	8.004381180	H
atom	30.977888107	6.099667549	8.004381180	H
atom	10.353515625	1.980432749	3.962580919	H
atom	17.429039001	7.146040440	6.546099663	H
atom	23.902362823	0.934059501	2.504299641	H
atom	17.429039001	0.934059501	2.504299641	H
atom	23.902362823	7.146040440	6.546099663	H
atom	38.094738007	3.105990648	6.546099663	H
atom	3.236662626	4.974109650	2.504299641	H
atom	38.094738007	4.974109650	2.504299641	H
atom	3.236662626	3.105990648	6.546099663	H
atom	17.324056625	5.993818283	7.607476234	H
atom	24.007345200	2.086281776	3.565675974	H
atom	17.324056625	2.086281776	3.565675974	H
atom	24.007345200	5.993818283	7.607476234	H
atom	37.989757538	1.953768730	7.607476234	H
atom	3.341644049	6.126331806	3.565675974	H
atom	37.989757538	6.126331806	3.565675974	H
atom	3.341644049	1.953768730	7.607476234	H
atom	17.904764175	4.508696079	6.046533108	H
atom	23.426637650	3.571403980	2.004733086	H
atom	17.904764175	3.571403980	2.004733086	H
atom	23.426637650	4.508696079	6.046533108	H
atom	38.570465088	0.468646526	6.046533108	H
atom	2.760937452	7.611454010	2.004733086	H

atom	38.570465088	7.611454010	2.004733086	H
atom	2.760937452	0.468646526	6.046533108	H
atom	17.830366135	5.486388206	4.974647522	H
atom	23.501035690	2.593711853	0.932848036	H
atom	17.830366135	2.593711853	0.932848036	H
atom	23.501035690	5.486388206	4.974647522	H
atom	38.496067047	1.446338058	4.974647522	H
atom	2.835334063	6.633761883	0.932848036	H
atom	38.496067047	6.633761883	0.932848036	H
atom	2.835334063	1.446338058	4.974647522	H
atom	18.890930176	5.575269222	5.965696812	H
atom	22.440471649	2.504831076	1.923896074	H
atom	18.890930176	2.504831076	1.923896074	H
atom	22.440471649	5.575269222	5.965696812	H
atom	39.556629181	1.535219431	5.965696812	H
atom	1.774770021	6.544880867	1.923896074	H
atom	39.556629181	6.544880867	1.923896074	H
atom	1.774770021	1.535219431	5.965696812	H
atom	15.395255089	4.319993019	7.528341770	H
atom	25.936147690	3.760107040	3.486541748	H
atom	15.395255089	3.760107040	3.486541748	H
atom	25.936147690	4.319993019	7.528341770	H
atom	36.060955048	0.279943436	7.528341770	H
atom	5.270445824	7.800157070	3.486541748	H
atom	36.060955048	7.800157070	3.486541748	H
atom	5.270445824	0.279943436	7.528341770	H
atom	18.036611557	5.361954689	5.830700874	N
atom	23.294790268	2.718145370	1.788900733	N
atom	18.036611557	2.718145370	1.788900733	N
atom	23.294790268	5.361954689	5.830700874	N
atom	38.702312469	1.321904063	5.830700874	N
atom	2.629090071	6.758195400	1.788900733	N
atom	38.702312469	6.758195400	1.788900733	N
atom	2.629090071	1.321904063	5.830700874	N

Supplementary Table 40 | Input geometry.in file of $[NMA]_2PbBr_4$ *relaxed* structure

lattice_vector	40.84784012	0.00324207	-0.00101373	
lattice_vector	-0.00057219	8.02401877	-0.00001719	
lattice_vector	0.00040172	-0.00002018	8.02784977	
atom	20.42363401	6.13078160	2.53691696	Pb
atom	20.42446475	1.89603802	6.55102000	Pb
atom	0.00075479	2.11733140	2.53756408	Pb
atom	0.00057631	5.90650611	6.55163729	Pb
atom	20.42429257	3.47177372	4.00717217	Br
atom	20.42416227	4.55515655	8.02092462	Br
atom	0.00036715	7.48233331	4.00791875	Br
atom	0.00098767	0.54160202	8.02180880	Br
atom	17.36796552	5.99636482	2.62484778	Br
atom	23.48058593	2.03088477	6.63830388	Br
atom	17.36842645	2.03054737	6.63871134	Br
atom	23.48014247	5.99639488	2.62421295	Br
atom	37.79249759	1.98591249	2.62412831	Br
atom	3.05669838	6.04125119	6.63916367	Br
atom	37.79239737	6.04419002	6.63811032	Br
atom	3.05678120	1.98310121	2.62519105	Br
atom	20.42499282	7.28328451	5.28140903	Br
atom	20.42520041	0.74369694	1.26768804	Br
atom	0.00067899	3.26968539	5.28217759	Br
atom	0.00045509	4.75426654	1.26844653	Br
atom	15.41851066	5.92223599	6.33596184	C
atom	25.43065000	2.10511731	2.32157490	C
atom	15.41869410	2.10448135	2.32218595	C
atom	25.43040481	5.92246086	6.33555690	C
atom	35.84269323	1.91161524	6.33523129	C
atom	5.00656621	6.11569728	2.32241986	C
atom	35.84239069	6.11821845	2.32146758	C
atom	5.00682060	1.90900265	6.33645443	C
atom	14.87614803	6.79026955	5.40647166	C
atom	25.97289247	1.23711354	1.39194666	C
atom	14.87641244	1.23644583	1.39264921	C
atom	25.97253748	6.79056071	5.40591721	C
atom	35.30037357	2.77970064	5.40573163	C
atom	5.54884881	5.24761188	1.39292089	C
atom	35.30020315	5.25012968	1.39189659	C
atom	5.54899245	2.77713724	5.40690291	C
atom	13.55316114	6.62382490	4.93198109	C
atom	27.29587438	1.40358327	0.91744766	C
atom	13.55340722	1.40278983	0.91820522	C
atom	27.29557270	6.62432182	4.93151921	C

atom	33.97737411	2.61327301	4.93127263	C
atom	6.87182220	5.41405054	0.91840038	C
atom	33.97716683	5.41635167	0.91747641	C
atom	6.87201690	2.61092968	4.93245722	C
atom	13.34302834	4.65924702	6.39205001	C
atom	27.50606146	3.36818431	2.37737973	C
atom	13.34317102	3.36728183	2.37833826	C
atom	27.50596481	4.65974888	6.39150540	C
atom	33.76727793	0.64857051	6.39116296	C
atom	7.08198544	7.37874206	2.37824264	C
atom	33.76677204	7.38088354	2.37751532	C
atom	7.08245622	0.64635401	6.39245334	C
atom	12.76908538	5.53144135	5.43094695	C
atom	28.07995785	2.49596312	1.41635821	C
atom	12.76925628	2.49503964	1.41726952	C
atom	28.07978414	5.53209774	5.43053003	C
atom	33.19333688	1.52084226	5.43016557	C
atom	7.65589752	6.50647184	1.41724230	C
atom	33.19295486	6.50858688	1.41647062	C
atom	7.65625856	1.51868496	5.43141359	C
atom	11.44470273	5.36798603	4.94822622	C
atom	29.40430415	2.65948283	0.93361334	C
atom	11.44481053	2.65834161	0.93472232	C
atom	29.40420400	5.36880934	4.94796369	C
atom	31.86897371	1.35732026	4.94744019	C
atom	8.98022860	6.66997916	0.93446104	C
atom	31.86853298	6.67185798	0.93383546	C
atom	8.98065527	1.35544424	4.94874148	C
atom	12.98646278	7.50258549	3.97469262	C
atom	27.86286902	0.52488509	7.98788801	C
atom	12.98714651	0.52402136	7.98873769	C
atom	27.86210842	7.50306989	3.97410857	C
atom	33.41068522	3.49203310	3.97398145	C
atom	7.43885198	4.53528451	7.98892498	C
atom	33.41098681	4.53752144	7.98801662	C
atom	7.43855234	3.48976295	3.97512343	C
atom	11.70125839	7.30956329	3.51944112	C
atom	29.14804869	0.71797138	7.53257950	C
atom	11.70187426	0.71689246	7.53364310	C
atom	29.14736668	7.31021336	3.51895886	C
atom	32.12550394	3.29893782	3.51868916	C
atom	8.72400499	4.72837525	7.53356332	C
atom	32.12572228	4.73035273	7.53284947	C
atom	8.72379543	3.29695524	3.51990555	C
atom	10.91938005	6.24058914	4.01815694	C
atom	29.92955907	1.78691591	0.00347136	C

atom	10.91949626	1.78571006	0.00467648	C
atom	29.92941906	6.24146377	4.01784362	C
atom	31.34365533	2.22992640	4.01735256	C
atom	9.50549758	5.79738427	0.00436069	C
atom	31.34330807	5.79918566	0.00376574	C
atom	9.50584598	2.22814203	4.01866304	C
atom	16.84988570	6.09395492	6.76250783	C
atom	23.99944901	1.93324906	2.74823391	C
atom	16.85005975	1.93290275	2.74860810	C
atom	23.99918882	6.09408208	6.76205844	C
atom	37.27395308	2.08343940	6.76182294	C
atom	3.57530195	5.94393309	2.74901122	C
atom	37.27369159	5.94663599	2.74801660	C
atom	3.57550328	2.08050486	6.76296402	C
atom	14.63165609	4.85117091	6.83877247	C
atom	26.21753054	3.17622395	2.82428348	C
atom	14.63180142	3.17550180	2.82505246	C
atom	26.21739419	4.85144157	6.83835264	C
atom	35.05586356	0.84047298	6.83796001	C
atom	5.79342644	7.18684146	2.82510307	C
atom	35.05540113	7.18917779	2.82427972	C
atom	5.79384104	0.83803065	6.83924890	C
atom	15.47035126	7.62393531	5.02824598	H
atom	25.37866592	0.40347930	1.01373319	H
atom	15.47071242	0.40287694	1.01437826	H
atom	25.37816075	7.62406661	5.02763864	H
atom	35.89459230	3.61339211	5.02757538	H
atom	4.95462036	4.41392845	1.01479562	H
atom	35.89457530	4.41661835	1.01363980	H
atom	4.95461435	3.61064924	5.02865960	H
atom	12.74909665	3.82963887	6.77618832	H
atom	28.09999137	4.19786957	2.76142509	H
atom	12.74918932	4.19683326	2.76251475	H
atom	28.10000429	3.83015764	6.77560766	H
atom	33.17334685	-0.18109267	6.77525545	H
atom	7.67592495	8.20842875	2.76227174	H
atom	33.17270978	8.21039949	2.76168129	H
atom	7.67652803	-0.18319113	6.77657458	H
atom	10.85080591	4.53030307	5.31334908	H
atom	29.99820669	3.49713685	1.29873064	H
atom	10.85083590	3.49591394	1.29994498	H
atom	29.99821391	4.53127178	5.31317347	H
atom	31.27510054	0.51960216	5.31249601	H
atom	9.57413272	7.50767564	1.29950210	H
atom	31.27451801	7.50942066	1.29898354	H
atom	9.57469182	0.51788683	5.31388113	H

atom	13.58970952	8.32799809	3.59947830	H
atom	27.25959140	-0.30048280	7.61264129	H
atom	13.59048710	-0.30125204	7.61337684	H
atom	27.25870993	8.32829660	3.59872354	H
atom	34.01391131	4.31748343	3.59880558	H
atom	6.83558832	3.70985732	7.61376760	H
atom	34.01435702	3.71224071	7.61273946	H
atom	6.83514986	4.31503298	3.59985428	H
atom	11.28785134	7.98255966	2.76906375	H
atom	29.56142316	0.04502790	6.78215116	H
atom	11.28847731	0.04389483	6.78327322	H
atom	29.56070128	7.98321347	2.76854436	H
atom	31.71208833	3.97193748	2.76832614	H
atom	9.13736175	4.05538507	6.78316500	H
atom	31.71237812	4.05729576	6.78250762	H
atom	9.13709045	3.97002852	2.76953105	H
atom	9.89804919	6.10608667	3.66322359	H
atom	30.95134062	1.92131438	7.67646083	H
atom	9.89847052	1.92001921	7.67777832	H
atom	30.95089325	6.10724972	3.66315493	H
atom	30.32230448	2.09545347	3.66244523	H
atom	10.52723206	5.93186507	7.67725290	H
atom	30.32229477	5.93342609	7.67680863	H
atom	10.52724572	2.09385998	3.66381505	H
atom	17.17785919	7.13545264	6.65080803	H
atom	23.67143596	0.89175792	2.63651446	H
atom	17.17817323	0.89148544	2.63681341	H
atom	23.67097564	7.13547555	6.65026774	H
atom	37.60192023	3.12494031	6.65007563	H
atom	3.24728636	4.90245002	2.63733073	H
atom	37.60179984	4.90518938	2.63631924	H
atom	3.24732880	3.12192416	6.65127091	H
atom	17.01916672	5.79128302	7.80423043	H
atom	23.83015115	2.23588164	3.78997510	H
atom	17.01934941	2.23550062	3.79035261	H
atom	23.82985509	5.79142641	7.80380017	H
atom	37.44322165	1.78082636	7.80357709	H
atom	3.40604486	6.24660591	3.79074335	H
atom	37.44295813	6.24931681	3.78974344	H
atom	3.40623462	1.77777926	7.80468414	H
atom	17.59343652	4.23202466	6.06186064	H
atom	23.25568895	3.79509389	2.04779194	H
atom	17.59363002	3.79498835	2.04810570	H
atom	23.25548508	4.23210729	6.06176587	H
atom	38.01792525	0.22158094	6.06145101	H
atom	2.83152883	7.80580865	2.04846139	H

atom	38.01728787	7.80858711	2.04747151	H
atom	2.83212144	0.21851244	6.06234609	H
atom	17.61429128	5.46754716	4.88751677	H
atom	23.23464031	2.55978636	0.87344262	H
atom	17.61398261	2.55964800	0.87360991	H
atom	23.23489594	5.46726140	4.88723258	H
atom	38.03839119	1.45684523	4.88694828	H
atom	2.81081050	6.57040799	0.87411733	H
atom	38.03797593	6.57326230	0.87307489	H
atom	2.81121267	1.45394426	4.88802371	H
atom	18.74982119	5.45228252	6.12608087	H
atom	22.09930682	2.57496433	2.11217250	H
atom	18.74980967	2.57466643	2.11187334	H
atom	22.09926432	5.45227821	6.12568819	H
atom	39.17399481	1.44203560	6.12540647	H
atom	1.67529365	6.58553932	2.11266428	H
atom	39.17360017	6.58839970	2.11152605	H
atom	1.67564797	1.43853349	6.12653662	H
atom	15.05209991	4.17144576	7.58122012	H
atom	25.79714544	3.85590843	3.56673484	H
atom	15.05223807	3.85527455	3.56739543	H
atom	25.79704829	4.17164708	7.58068160	H
atom	35.47630445	0.16071258	7.58031681	H
atom	5.37301971	7.86662210	3.56745174	H
atom	35.47574430	7.86894752	3.56667465	H
atom	5.37352008	0.15821349	7.58163020	H
atom	17.75642657	5.25644525	5.90366836	N
atom	23.09269588	2.77079545	1.88954834	N
atom	17.75641910	2.77058796	1.88976243	N
atom	23.09265055	5.25638364	5.90334162	N
atom	38.18063353	1.24594278	5.90310185	N
atom	2.66866790	6.78144312	1.89024443	N
atom	38.18019894	6.78425165	1.88920526	N
atom	2.66906099	1.24284986	5.90413831	N

Supplementary Table 41 | Input geometry.in file of $[PMA]_2PbCl_4$ *experimental* structure

lattice_vector	33.6349983215	0.0000000000	0.0000000000	
lattice_vector	0.0000000000	7.8169999123	0.0000000000	
lattice_vector	0.0000000000	0.0000000000	7.7369999886	
atom	16.817499161	1.865839720	0.020116201	Pb
atom	16.817499161	5.951159954	3.888616323	Pb
atom	0.000000000	5.774339676	0.020116201	Pb
atom	0.000000000	2.042659998	3.888616323	Pb
atom	16.817499161	0.665226698	5.180695057	Cl
atom	16.817499161	7.151773453	1.312195301	Cl
atom	0.000000000	4.573726654	5.180695057	Cl
atom	0.000000000	3.243273258	1.312195301	Cl
atom	19.676137924	2.112935066	7.583807468	Cl
atom	13.958859444	5.704064369	3.715307236	Cl
atom	19.676137924	5.704064369	3.715307236	Cl
atom	13.958859444	2.112935066	7.583807468	Cl
atom	2.858639240	6.021435261	7.583807468	Cl
atom	30.776359558	1.795564890	3.715307236	Cl
atom	2.858639240	1.795564890	3.715307236	Cl
atom	30.776359558	6.021435261	7.583807468	Cl
atom	16.817499161	4.346251965	6.292502403	Cl
atom	16.817499161	3.470747948	2.424002171	Cl
atom	0.000000000	0.437751949	6.292502403	Cl
atom	0.000000000	7.379248142	2.424002171	Cl
atom	20.123819351	1.670492887	3.572946548	C
atom	13.511179924	6.146507263	7.441446304	C
atom	20.123819351	6.146507263	7.441446304	C
atom	13.511179924	1.670492887	3.572946548	C
atom	3.306319714	5.578992844	3.572946548	C
atom	30.328678131	2.238007069	7.441446304	C
atom	3.306319714	2.238007069	7.441446304	C
atom	30.328678131	5.578992844	3.572946548	C
atom	21.600395203	1.786184430	3.930396080	C
atom	12.034602165	6.030815601	0.061896127	C
atom	21.600395203	6.030815601	0.061896127	C
atom	12.034602165	1.786184430	3.930396080	C
atom	4.782896519	5.694684505	3.930396080	C
atom	28.852102280	2.122315407	0.061896127	C
atom	4.782896519	2.122315407	0.061896127	C
atom	28.852102280	5.694684505	3.930396080	C
atom	22.185646057	0.973216474	4.803129673	C
atom	11.449353218	6.843783379	0.934629738	C
atom	22.185646057	6.843783379	0.934629738	C
atom	11.449353218	0.973216474	4.803129673	C

atom	5.368146420	4.881716251	4.803129673	C
atom	28.266851425	2.935283422	0.934629738	C
atom	5.368146420	2.935283422	0.934629738	C
atom	28.266851425	4.881716251	4.803129673	C
atom	23.541133881	1.080309391	5.114157200	C
atom	10.093863487	6.736690521	1.245657086	C
atom	23.541133881	6.736690521	1.245657086	C
atom	10.093863487	1.080309391	5.114157200	C
atom	6.723633289	4.988809109	5.114157200	C
atom	26.911363602	2.828190804	1.245657086	C
atom	6.723633289	2.828190804	1.245657086	C
atom	26.911363602	4.988809109	5.114157200	C
atom	24.301286697	2.045708895	4.487459660	C
atom	9.333711624	5.771290779	0.618959427	C
atom	24.301286697	5.771290779	0.618959427	C
atom	9.333711624	2.045708895	4.487459660	C
atom	7.483789921	5.954209328	4.487459660	C
atom	26.151210785	1.862791061	0.618959427	C
atom	7.483789921	1.862791061	0.618959427	C
atom	26.151210785	5.954209328	4.487459660	C
atom	23.739582062	2.914177418	3.636389971	C
atom	9.895416260	4.902822495	7.504890442	C
atom	23.739582062	4.902822495	7.504890442	C
atom	9.895416260	2.914177418	3.636389971	C
atom	6.922084332	6.822677612	3.636389971	C
atom	26.712915421	0.994322419	7.504890442	C
atom	6.922084332	0.994322419	7.504890442	C
atom	26.712915421	6.822677612	3.636389971	C
atom	22.380729675	2.761746168	3.306020260	C
atom	11.254269600	5.055253983	7.174520016	C
atom	22.380729675	5.055253983	7.174520016	C
atom	11.254269600	2.761746168	3.306020260	C
atom	5.563229561	6.670245647	3.306020260	C
atom	28.071767807	1.146753788	7.174520016	C
atom	5.563229561	1.146753788	7.174520016	C
atom	28.071767807	6.670245647	3.306020260	C
atom	19.992643356	1.898749232	2.639090776	H
atom	13.642355919	5.918250561	6.507590294	H
atom	19.992643356	5.918250561	6.507590294	H
atom	13.642355919	1.898749232	2.639090776	H
atom	3.175141573	5.807249546	2.639090776	H
atom	30.459854126	2.009750605	6.507590294	H
atom	3.175141573	2.009750605	6.507590294	H
atom	30.459854126	5.807249546	2.639090776	H
atom	19.824468613	0.758248985	3.706796646	H
atom	13.810530663	7.058751106	7.575296402	H

atom	19.824468613	7.058751106	7.575296402	H
atom	13.810530663	0.758248985	3.706796646	H
atom	3.006970644	4.666749001	3.706796646	H
atom	30.628028870	3.150250912	7.575296402	H
atom	3.006970644	3.150250912	7.575296402	H
atom	30.628028870	4.666749001	3.706796646	H
atom	18.475704193	2.511602163	4.220533371	H
atom	15.159293175	5.305397511	0.352033824	H
atom	18.475704193	5.305397511	0.352033824	H
atom	15.159293175	2.511602163	4.220533371	H
atom	1.658203959	6.420102119	4.220533371	H
atom	31.976793289	1.396897912	0.352033824	H
atom	1.658203959	1.396897912	0.352033824	H
atom	31.976793289	6.420102119	4.220533371	H
atom	19.609203339	3.421500921	4.286298275	H
atom	14.025794029	4.395498753	0.417798162	H
atom	19.609203339	4.395498753	0.417798162	H
atom	14.025794029	3.421500921	4.286298275	H
atom	2.791702986	7.330000877	4.286298275	H
atom	30.843294144	0.486999065	0.417798162	H
atom	2.791702986	0.486999065	0.417798162	H
atom	30.843294144	7.330000877	4.286298275	H
atom	19.461210251	2.370896101	5.279728889	H
atom	14.173789024	5.446103573	1.411228657	H
atom	19.461210251	5.446103573	1.411228657	H
atom	14.173789024	2.370896101	5.279728889	H
atom	2.643708467	6.279396057	5.279728889	H
atom	30.991287231	1.537603974	1.411228657	H
atom	2.643708467	1.537603974	1.411228657	H
atom	30.991287231	6.279396057	5.279728889	H
atom	21.681119919	0.315025091	5.220927238	H
atom	11.953879356	7.501974583	1.352427006	H
atom	21.681119919	7.501974583	1.352427006	H
atom	11.953879356	0.315025091	5.220927238	H
atom	4.863617897	4.223525047	5.220927238	H
atom	28.771377563	3.593474865	1.352427006	H
atom	4.863617897	3.593474865	1.352427006	H
atom	28.771377563	4.223525047	5.220927238	H
atom	23.927936554	0.505759895	5.730795860	H
atom	9.707061768	7.311240196	1.862295985	H
atom	23.927936554	7.311240196	1.862295985	H
atom	9.707061768	0.505759895	5.730795860	H
atom	7.110439777	4.414259911	5.730795860	H
atom	26.524560928	3.402740002	1.862295985	H
atom	7.110439777	3.402740002	1.862295985	H
atom	26.524560928	4.414259911	5.730795860	H

atom	25.212795258	2.095737696	4.660768986	H
atom	8.422204018	5.721261978	0.792269289	H
atom	25.212795258	5.721261978	0.792269289	H
atom	8.422204018	2.095737696	4.660768986	H
atom	8.395293236	6.004237652	4.660768986	H
atom	25.239702225	1.812762380	0.792269289	H
atom	8.395293236	1.812762380	0.792269289	H
atom	25.239702225	6.004237652	4.660768986	H
atom	24.240743637	3.605982065	3.265787840	H
atom	9.394253731	4.211017609	7.134287834	H
atom	24.240743637	4.211017609	7.134287834	H
atom	9.394253731	3.605982065	3.265787840	H
atom	7.423244953	7.514482021	3.265787840	H
atom	26.211753845	0.302518010	7.134287834	H
atom	7.423244953	0.302518010	7.134287834	H
atom	26.211753845	7.514482021	3.265787840	H
atom	21.997287750	3.316752911	2.665396452	H
atom	11.637709618	4.500247002	6.533896446	H
atom	21.997287750	4.500247002	6.533896446	H
atom	11.637709618	3.316752911	2.665396452	H
atom	5.179791451	7.225252628	2.665396452	H
atom	28.455209732	0.591746986	6.533896446	H
atom	5.179791451	0.591746986	6.533896446	H
atom	28.455209732	7.225252628	2.665396452	H
atom	19.340124130	2.584300041	4.424016476	N
atom	14.294875145	5.232699394	0.555516541	N
atom	19.340124130	5.232699394	0.555516541	N
atom	14.294875145	2.584300041	4.424016476	N
atom	2.522626400	6.492800236	4.424016476	N
atom	31.112373352	1.324199796	0.555516541	N
atom	2.522626400	1.324199796	0.555516541	N
atom	31.112373352	6.492800236	4.424016476	N

Supplementary Table 42 | Input geometry.in file of $[PMA]_2PbCl_4$ *relaxed* structure

lattice_vector	33.29337475	0.01716731	0.02982197	
lattice_vector	-0.00373463	7.75217677	0.07776102	
lattice_vector	-0.00561572	-0.07587350	7.60582442	
atom	16.64049508	5.90463660	3.02857467	Pb
atom	16.64297545	1.77241332	6.79037032	Pb
atom	-0.00323312	2.01863699	2.96938559	Pb
atom	-0.00771034	5.63789967	6.80884448	Pb
atom	19.55231128	5.68978911	2.88306979	Cl
atom	13.73115547	1.99169678	6.64416719	Cl
atom	19.55390405	1.99615800	6.64973573	Cl
atom	13.72934378	5.68428244	2.87800035	Cl
atom	2.90760154	1.80236047	2.82344460	Cl
atom	30.37499183	5.87483199	6.69360607	Cl
atom	2.90274603	5.86038249	6.66751575	Cl
atom	30.37964383	1.81703931	2.84945293	Cl
atom	16.64529819	0.69824026	4.14863602	Cl
atom	-0.00204246	3.14524560	0.34931951	Cl
atom	-0.00559344	4.56295812	4.16774908	Cl
atom	16.64205083	4.32802008	5.41121559	Cl
atom	-0.00450454	0.44119749	5.35155008	Cl
atom	19.21934991	5.07166024	7.36316823	N
atom	14.06333681	2.59548400	3.53012227	N
atom	19.22392778	2.59997128	3.53527716	N
atom	14.06006000	5.06729306	7.35848951	N
atom	2.57088477	1.18333390	7.30367590	N
atom	30.71022754	6.48010558	3.57981581	N
atom	2.57130087	6.46512133	3.55332270	N
atom	30.70995020	1.19816847	7.32998938	N
atom	18.22359914	5.24636031	7.14132520	H
atom	15.05955087	2.42538712	3.30678232	H
atom	18.22845910	2.42881952	3.30933830	H
atom	15.05609771	5.24302462	7.13871262	H
atom	1.57506954	1.35785858	7.08210349	H
atom	31.70610033	6.30991649	3.35512078	H
atom	1.57580886	6.29419892	3.32751771	H
atom	31.70581934	1.37383971	7.10954072	H
atom	13.92919674	2.39849311	4.55040609	H
atom	19.35624444	2.40297241	4.55581517	H
atom	30.57676067	6.28268885	4.60007262	H
atom	2.70375804	6.26785273	4.57374169	H
atom	19.40777560	4.05902573	7.16977684	H
atom	13.87498288	3.61182900	3.35661972	H
atom	19.41102811	3.61658166	3.36259891	H

atom	13.87320343	4.05435237	7.16472539	H
atom	2.75961460	0.17079729	7.10997735	H
atom	30.52196395	7.49649068	3.40697452	H
atom	2.75902097	7.48163751	3.38064717	H
atom	30.52262461	0.18540598	7.13608965	H
atom	20.10074378	5.96614969	6.54067035	C
atom	13.18382493	1.71719381	2.68835601	C
atom	20.10663003	1.72325558	2.69522944	C
atom	13.17978222	5.96118075	6.53417011	C
atom	3.45169562	2.07845906	6.48124790	C
atom	29.82934494	5.60259722	2.73868013	C
atom	3.45372947	5.58819906	2.71320225	C
atom	29.82899026	2.09230708	6.50663692	C
atom	19.96180550	5.68057462	5.49023870	H
atom	13.32525718	2.02293734	1.64395368	H
atom	19.96768330	2.02970495	1.65070543	H
atom	13.32144390	5.67617538	5.48396177	H
atom	3.31283667	1.79311440	5.43074845	H
atom	29.96938322	5.90900096	1.69429063	H
atom	3.31435280	5.89420850	1.66860718	H
atom	29.96901848	1.80690001	5.45631086	H
atom	19.73073516	6.99052889	6.67205689	H
atom	13.55348225	0.69045297	2.80089695	H
atom	19.73772379	0.69603173	2.80596142	H
atom	13.54857350	6.98581959	6.66694834	H
atom	3.08122070	3.10260651	6.61301832	H
atom	30.19880757	4.57565734	2.85006954	H
atom	3.08513118	4.56094307	2.82451753	H
atom	30.19841650	3.11681906	6.63854242	H
atom	21.53340578	5.84670325	6.98368463	C
atom	11.74999639	1.82818796	3.12986995	C
atom	21.53910473	1.83544519	3.14079556	C
atom	11.74604013	5.84033157	6.97335818	C
atom	4.88435022	1.95941414	6.92423024	C
atom	28.39642292	5.71413040	3.18277565	C
atom	4.88616887	5.70117337	3.15848287	C
atom	28.39607368	1.97204439	6.94845716	C
atom	11.24333778	0.92058041	4.06849587	C
atom	22.04423909	0.92745192	4.07988716	C
atom	27.89095534	4.80622993	4.12173897	C
atom	5.39187427	4.79373077	4.09775366	C
atom	11.87796089	0.11074180	4.42523409	H
atom	21.40943311	0.11659966	4.43396561	H
atom	28.52517648	3.99504059	4.47609777	H
atom	4.75826202	3.98184906	4.45162821	H
atom	9.94251095	1.05163880	4.54962838	C

atom	23.34346024	1.05971419	4.56498367	C
atom	26.59131608	4.93812153	4.60585720	C
atom	6.69107404	4.92687668	4.58274714	C
atom	9.57331845	0.34886672	5.29532670	H
atom	23.71139464	0.35680139	5.31116777	H
atom	26.22313915	4.23508965	5.35181312	H
atom	7.05944421	4.22411708	5.32886507	H
atom	24.15276056	5.57013951	7.94214574	C
atom	9.12760250	2.08601287	4.08538117	C
atom	24.15835066	2.09561870	4.10412773	C
atom	9.12373759	5.56190193	7.92331283	C
atom	7.50382038	1.68371727	7.88254962	C
atom	25.77643258	5.97364637	4.14423919	C
atom	7.50522756	5.96328138	4.12182253	C
atom	25.77610666	1.69414614	7.90477410	C
atom	8.10692462	2.18176813	4.45494392	H
atom	25.17786157	2.19230447	4.47665215	H
atom	24.75657433	6.07005195	4.51584398	H
atom	8.52474248	6.06070649	4.49411482	H
atom	23.65974660	4.68334377	6.98213356	C
atom	9.62320976	2.99080410	3.14369629	C
atom	23.66428744	3.00077968	3.16199081	C
atom	9.62023897	4.67592292	6.96433934	C
atom	7.01065344	0.79614741	6.92332048	C
atom	26.27086817	6.87874421	3.20224934	C
atom	7.01049412	6.86800507	3.17961936	C
atom	26.27073097	0.80702774	6.94588077	C
atom	24.28582117	3.87419141	6.61026394	H
atom	8.99804105	3.80651574	2.78483702	H
atom	24.28948517	3.81762457	2.80580043	H
atom	8.99568880	3.86681586	6.58980103	H
atom	7.63674527	-0.01329168	6.55213679	H
atom	25.64565056	7.69543132	2.84575653	H
atom	7.63506233	7.68543008	2.82367255	H
atom	25.64554487	-0.00288753	6.57421921	H
atom	22.35741087	4.82052764	6.50143701	C
atom	10.92704676	2.86296978	2.66446997	C
atom	22.36199122	2.87182043	2.67890631	C
atom	10.92404203	4.81404622	6.48789661	C
atom	5.70828872	0.93288781	6.44265106	C
atom	27.57355205	6.75018563	2.72014382	C
atom	5.70831594	6.73810925	2.69652483	C
atom	27.57336960	0.94482931	6.46624104	C
atom	21.98121368	4.11881605	5.75530932	H
atom	11.30522756	3.57852433	1.93262280	H
atom	21.98492337	3.58773420	1.94683572	H

atom	11.30283724	4.11315290	5.74230614	H
atom	5.33229515	0.23075140	5.69681736	H
atom	27.95072237	7.46608724	1.98812256	H
atom	5.33093323	7.45366096	1.96427225	H
atom	27.95052468	0.24301105	5.72069360	H
atom	16.64343658	-0.79630537	7.93645629	Cl
atom	16.63947427	3.30118081	9.20448010	Cl
atom	-0.00123021	-0.50960172	1.53922410	Cl
atom	22.03753163	6.73647871	7.94060139	C
atom	11.23846820	6.72919465	7.92930190	C
atom	5.38847791	2.84977129	7.88058663	C
atom	27.89044704	2.86198841	7.90441114	C
atom	23.33682526	6.59596911	8.42320177	C
atom	25.17231968	5.46700422	8.31279063	H
atom	8.10307451	5.45808397	8.29072745	H
atom	9.93769389	6.58778254	8.40763207	C
atom	6.68790003	2.70979984	8.36302942	C
atom	8.52350793	1.58092014	8.25291961	H
atom	24.75622768	1.59050170	8.27437374	H
atom	26.59079453	2.72090447	8.38589321	C
atom	21.40226096	7.53998891	8.31023567	H
atom	11.87219154	7.53270390	8.30160884	H
atom	4.75321154	3.65349582	8.24975656	H
atom	28.52474579	3.66626630	8.27403115	H
atom	23.70395265	7.28450519	9.18306927	H
atom	9.56778366	7.27566784	9.16673931	H
atom	7.05499783	3.39894318	9.12237030	H
atom	26.22254001	3.40973770	9.14495194	H
atom	19.35163286	5.24790226	8.38753409	H
atom	13.92525271	5.24382288	8.38240877	H
atom	2.70366958	1.35961118	8.32790800	H
atom	30.57582406	1.37429856	8.35406269	H

Supplementary Table 43 | Input geometry.in file of $(FC_2H_4NH_3)_2PbCl_4$ *relaxed* structure

```
lattice_vector    18.4573993683     0.0000000000     0.0000000000
lattice_vector     0.0000000000     8.7524995804     0.0000000000
lattice_vector     0.0000000000     0.0000000000     7.7059001923

#   C   H  Cl   F   N  Pb
#  16  56  16   8   8   4

#Cartesian
atom    4.614349842    4.420721054    2.705179453 Pb
atom   13.843050003    4.331778526    5.000720978 Pb
atom   13.843050003    0.044470910    1.147770762 Pb
atom    4.614349842    8.708027840    6.558129311 Pb
atom    4.614349842    6.389061928    4.553493500 Cl
atom   13.843050003    2.363437653    3.152406693 Cl
atom   13.843050003    2.012812138    7.005356789 Cl
atom    4.614349842    6.739687443    0.700543046 Cl
atom    4.614349842    2.603518486    4.751843452 Cl
atom   13.843050003    6.148981571    2.954056978 Cl
atom   13.843050003    6.979767799    6.807006836 Cl
atom    4.614349842    1.772731304    0.898893118 Cl
atom    7.492965698    4.652390957    2.715096951 Cl
atom   10.964434624    4.100108624    4.990803242 Cl
atom   10.964434624    0.276140749    1.137853146 Cl
atom    7.492965698    8.476358414    6.568047047 Cl
atom   16.721664429    4.100108624    4.990803242 Cl
atom    1.735733986    4.652390957    2.715096951 Cl
atom    1.735733986    8.476358414    6.568047047 Cl
atom   16.721664429    0.276140749    1.137853146 Cl
atom    7.118280411    4.640575409    6.654815674 C
atom   11.339118958    4.111924171    1.051084638 C
atom   11.339118958    0.264325529    4.904034615 C
atom    7.118280411    8.488174438    2.801865339 C
atom   16.346981049    4.111924171    1.051084638 C
atom    2.110419273    4.640575409    6.654815674 C
atom    2.110419273    8.488174438    2.801865339 C
atom   16.346981049    0.264325529    4.904034615 C
atom    7.446269035    3.179783106    6.620909214 C
atom   11.011131287    5.572716713    1.084990740 C
atom   11.011131287    7.556032658    4.937941074 C
atom    7.446269035    1.196466684    2.767959356 C
atom   16.674968719    5.572716713    1.084990740 C
atom    1.782430768    3.179783106    6.620909214 C
atom    1.782430768    1.196466684    2.767959356 C
atom   16.674968719    7.556032658    4.937941074 C
```

atom	6.185074329	4.776238918	6.354285240	H
atom	12.272324562	3.976260424	1.351615071	H
atom	12.272324562	0.399989873	5.204565048	H
atom	6.185074329	8.352510452	2.501335144	H
atom	15.413774490	3.976260424	1.351615071	H
atom	3.043625355	4.776238918	6.354285240	H
atom	3.043625355	8.352510452	2.501335144	H
atom	15.413774490	0.399989873	5.204565048	H
atom	7.192848682	4.973170280	7.584146976	H
atom	11.264551163	3.779329300	0.121753216	H
atom	11.264551163	0.596920431	3.974703312	H
atom	7.192848682	8.155579567	3.731196880	H
atom	16.421548843	3.779329300	0.121753216	H
atom	2.035851240	4.973170280	7.584146976	H
atom	2.035851240	8.155579567	3.731196880	H
atom	16.421548843	0.596920431	3.974703312	H
atom	6.738796234	2.666011333	7.086345673	H
atom	11.718602180	6.086488247	0.619554281	H
atom	11.718602180	7.042261124	4.472504616	H
atom	6.738796234	1.710238457	3.233395815	H
atom	15.967496872	6.086488247	0.619554281	H
atom	2.489903212	2.666011333	7.086345673	H
atom	2.489903212	1.710238457	3.233395815	H
atom	15.967496872	7.042261124	4.472504616	H
atom	7.490013123	2.867318869	5.682330608	H
atom	10.967387199	5.885180473	2.023569345	H
atom	10.967387199	7.243568897	5.876519680	H
atom	7.490013123	1.508930922	1.829381108	H
atom	16.718711853	5.885180473	2.023569345	H
atom	1.738686800	2.867318869	5.682330608	H
atom	1.738686800	1.508930922	1.829381108	H
atom	16.718711853	7.243568897	5.876519680	H
atom	7.844395161	5.225242138	4.885540962	H
atom	10.613004684	3.527257204	2.820359468	H
atom	10.613004684	0.848992467	6.673309326	H
atom	7.844395161	7.903507233	1.032591105	H
atom	17.073095322	3.527257204	2.820359468	H
atom	1.384304762	5.225242138	4.885540962	H
atom	1.384304762	7.903507233	1.032591105	H
atom	17.073095322	0.848992467	6.673309326	H
atom	8.854014397	5.207737446	5.964366436	H
atom	9.603385925	3.544762135	1.741533518	H
atom	9.603385925	0.831487715	5.594483852	H
atom	8.854014397	7.921011925	2.111416101	H
atom	18.082714081	3.544762135	1.741533518	H
atom	0.374685228	5.207737446	5.964366436	H

atom	0.374685228	7.921011925	2.111416101	H
atom	18.082714081	0.831487715	5.594483852	H
atom	7.920070171	6.301799774	5.956660748	H
atom	10.537328720	2.450699568	1.749239326	H
atom	10.537328720	1.925550103	5.602189541	H
atom	7.920070171	6.826949596	2.103710651	H
atom	17.148771286	2.450699568	1.749239326	H
atom	1.308629513	6.301799774	5.956660748	H
atom	1.308629513	6.826949596	2.103710651	H
atom	17.148771286	1.925550103	5.602189541	H
atom	8.689189911	2.964909315	7.253872395	F
atom	9.768209457	5.787590504	0.452027887	F
atom	9.768209457	7.341158867	4.304977894	F
atom	8.689189911	1.411340475	3.400921822	F
atom	17.917890549	5.787590504	0.452027887	F
atom	0.539509773	2.964909315	7.253872395	F
atom	0.539509773	1.411340475	3.400921822	F
atom	17.917890549	7.341158867	4.304977894	F
atom	8.036352158	5.411670685	5.778654575	N
atom	10.421047211	3.340828896	1.927245736	N
atom	10.421047211	1.035420418	5.780195713	N
atom	8.036352158	7.717078686	1.925704360	N
atom	17.265050888	3.340828896	1.927245736	N
atom	1.192347884	5.411670685	5.778654575	N
atom	1.192347884	7.717078686	1.925704360	N
atom	17.265050888	1.035420418	5.780195713	N

Supplementary References

1 Mitzi, D. B. Synthesis, Structure, and Properties of Organic-Inorganic Perovskites and Related Materials. *Prog. Inorg. Chem.* **48**, 1-121, (1999).

2 Saparov, B. & Mitzi, D. B. Organic–Inorganic Perovskites: Structural Versatility for Functional Materials Design. *Chem. Rev.* **116**, 4558-4596, (2016).

3 Smith, M. D., Jaffe, A., Dohner, E. R., Lindenberg, A. M. & Karunadasa, H. I. Structural origins of broadband emission from layered Pb–Br hybrid perovskites. *Chem. Sci.* **8**, 4497-4504, (2017).

4 Du, K.-z. *et al.* Two-Dimensional Lead(II) Halide-Based Hybrid Perovskites Templated by Acene Alkylamines: Crystal Structures, Optical Properties, and Piezoelectricity. *Inorg. Chem.* **56**, 9291-9302, (2017).

5 Braun, M. & Frey, W. Crystal structure of bis(2-naphthylmethylammonium) lead tetra-chloride, $(C_{11}H_9NH_3)_2PbCl_4$. *Z. Kristallogr. NCS* **214**, 333-334, (1999).

6 Lermer, C. *et al.* Toward Fluorinated Spacers for MAPI-Derived Hybrid Perovskites: Synthesis, Characterization, and Phase Transitions of $(FC_2H_4NH_3)_2PbCl_4$. *Chem. Mater.* **28**, 6560– 6566, (2016).

7 Billing, D. G. & Lemmerer, A. Inorganic–organic hybrid materials incorporating primary cyclic ammonium cations: the lead bromide and chloride series. *CrystEngComm* **11**, 1549– 1562, (2009).

8 Shibuya, K., Koshimizu, M., Nishikido, F., Saito, H. & Kishimoto, S. Poly[bis(phenethylammonium) [dibromidoplumbate(II)]-di-[mu]-bromido]]. *Acta Cryst.* **65**, m1323-m1324, (2009).

9 Mao, L., Wu, Y., Stoumpos, C. C., Wasielewski, M. R. & Kanatzidis, M. G. White-Light Emission and Structural Distortion in New Corrugated Two-Dimensional Lead Bromide Perovskites. *J. Am. Chem. Soc.* **139**, 5210-5215, (2017).

10 Jana, M. K. *et al.* Organic-to-inorganic structural chirality transfer in a 2D hybrid perovskite and impact on Rashba-Dresselhaus spin-orbit coupling *Nat. Commun.* **11**, 4699, (2020).

11 Dohner, E. R., Hoke, E. T. & Karunadasa, H. I. Self-assembly of broadband white-light emitters. *J. Am. Chem. Soc.* **136**, 1718-1721, (2014).

12 Solis-Ibarra, D., Smith, I. C. & Karunadasa, H. I. Post-Synthetic Halide Conversion and Selective Halogen Capture in Hybrid Perovskites. *Chem. Sci.* **6**, 4054−4059, (2015).

13 Solis-Ibarra, D. & Karunadasa, H. I. Reversible and Irreversible Chemisorption in Nonporous-Crystalline Hybrids. *Angew. Chem., Int. Ed.* **53**, 1039−1042, (2014).

14 Hautzinger, M. P. *et al.* Two-Dimensional Lead Halide Perovskites Templated by a Conjugated Asymmetric Diammonium. *Inorg. Chem.* **56**, 14991– 14998, (2017).

15 Papavassiliou, G. C., Mousdis, G. A., Raptopoulou, C. P. & Terzis, A. Preparation and Characterization of $[C_6H_5CH_2NH_3]_2PbI_4$, $[C_6H_5CH_2CH_2SC(NH_2)_2]_3PbI_5$ and $[C_{10}H_7CH_2NH_3]PbI_3$ Organic-Inorganic Hybrid Compounds. *Z. Naturforsch. B* **54**, 1405-1409, (1999).

16 Tremblay, M.-H. *et al.* Structures of $(4\text{-}Y\text{-}C_6H_4CH_2NH_3)_2PbI_4$ {Y = H, F, Cl, Br, I}: Tuning of Hybrid Organic Inorganic Perovskite Structures from Ruddlesden–Popper to Dion–Jacobson Limits. *Chem. Mater.* **31**, 6145– 6153, (2019).

17 Billing, D. G. & Lemmerer, A. Poly[bis-[2-(1-cyclo-hexen-yl)ethyl-ammonium] di-[mu]-iodo-diodo-plumbate(II)]. *Acta Cryst.* **C62**, m269-m271, (2006).

18 Rayner, M. K. & Billing, D. G. Poly[1,4-bis-(ammonio-meth-yl)cyclo-hexane [di-[m]-iodido-diiodido-plumbate(II)]]. *Acta Cryst.* **E66**, m660, (2010).

19 Lemmerer, A. & Billing, D. G. Effect of heteroatoms in the inorganic–organic layered perovskite-type hybrids $[(ZC_nH_{2n}NH_3)_2PbI_4]$, n = 2, 3, 4, 5, 6; Z = OH, Br and I; and $[(H_3NC_2H_4S_2C_2H_4NH_3)PbI_4]$. *CrystEngComm* **12**, 1290– 1301, (2010).

20 Zhu, X.-H. *et al.* Effect of Mono- versus Di-ammonium Cation of 2,2‘-Bithiophene Derivatives on the Structure of Organic−Inorganic Hybrid Materials Based on Iodo Metallates. *Inorg. Chem.* **42**, 5330-5339, (2003).

21 Li, T., Dunlap-Shohl, W. A., Reinheimer, E. W., Le Magueres, P. & Mitzi, D. B. Melting temperature suppression of layered hybrid lead halide perovskites via organic ammonium cation branching. *Chem. Sci.* **10**, 1168– 1175, (2019).

22 Billing, D. G. & Lemmerer, A. Inorganic–Organic Hybrid Materials Incorporating Primary Cyclic Ammonium Cations: The Lead Iodide Series. *CrystEngComm* **9**, 236– 244, (2007).

23 Booker, E. P. *et al.* Formation of Long-Lived Color Centers for Broadband Visible Light Emission in Low-Dimensional Layered Perovskites. *J. Am. Chem. Soc.* **139**, 18632–18639, (2017).

24 Safdari, M. *et al.* Layered 2D Alkyldiammonium Lead Iodide Perov- skites: Synthesis, Characterization, and Use in Solar Cells. *J. Mater. Chem. A.* **4**, 15638−15646, (2016).

25 Zimmermann, I., Aghazada, S. & Nazeeruddin, M. K. Lead and HTM Free Stable Two-Dimensional Tin Perovskites with Suitable Band Gap for Solar Cell Applications. *Angew. Chem. Int. Ed.* **58**, 1072-1076, (2019).

26 Passarelli, J. V. *et al.* Enhanced Out-of-Plane Conductivity and Photovoltaic Performance in n = 1 Layered Perovskites through Organic Cation Design. *J. Am. Chem. Soc.* **140**, 7313-7323, (2018).

27 Passarelli, J. V. *et al.* Tunable exciton binding energy in 2D hybrid layered perovskites through donor–acceptor interactions within the organic layer. *Nat. Chem.* **12**, 672–682, (2020).

28 Wood, E. A. Vocabulary of Surface Crystallography. *J. Appl. Phys.* **35**, 1306-1312, (1964).

29 Hermann, K. *Crystallography and Surface Structure: An Introduction for Surface Scientists and Nanoscientists*. (Wiley-VCH Verlag GmbH & Co. KGaA, 2017).

30 Knutson, J. L., Martin, J. D. & Mitzi, D. B. Tuning the Band Gap in Hybrid Tin Iodide Perovskite Semiconductors Using Structural Templating. *Inorg. Chem.* **44**, 4699-4705, (2005).

31 Even, J., Pedesseau, L., Dupertuis, M. A., Jancu, J. M. & Katan, C. Electronic model for self-assembled hybrid organic/perovskite semiconductors: Reverse band edge electronic states ordering and spin-orbit coupling. *Phys. Rev. B* **86**, 205301, (2012).

32 Sercel, P. C., Lyons, J. L., Bernstein, N. & Efros, A. L. Quasicubic model for metal halide perovskite nanocrystals. *J. Chem. Phys.* **151**, 234106, (2019).

33 Nagamune, Y., Takeyama, S. & Miura, N. Exciton spectra and anisotropic Zeeman effect in PbI_2 at high magnetic fields up to 40 T. *Phys. Rev. B* **43**, 12401-12405, (1991).

34 Tanaka, K. *et al.* Electronic and Excitonic Structures of Inorganic–Organic Perovskite-Type Quantum-Well Crystal $(C_4H_9NH_3)_2PbBr_4$. *Jpn. J. Appl. Phys.* **44**, 5923-5932, (2005).

35 Luttinger, J. M. Quantum Theory of Cyclotron Resonance in Semiconductors: General Theory. *Phys. Rev.* **102**, 1030-1041, (1956).

36 Ema, K. *et al.* Huge exchange energy and fine structure of excitons in an organic-inorganic quantum well material. *Phys. Rev. B* **73**, 241310, (2006).
